# The color appearance of curved transparent objects

**DOI:** 10.1167/jov.21.5.20

**Published:** 2021-05-19

**Authors:** Robert Ennis, Katja Doerschner

**Affiliations:** 1 Justus-Liebig-Universitaet Giessen, Department of General Psychology, Giessen, Germany; 1 National Magnetic Resonance Research Center, Bilkent University, Ankara, Turkey

**Keywords:** transparency, cone ratios, glass color, image statistics, color matching

## Abstract

Studies on colored transparent objects have elucidated potential mechanisms, but these studies have mainly focused on flat filters overlaying flat backgrounds. While they have provided valuable insight, these studies have not captured all aspects of transparency, like caustics, specular reflections/highlights, and shadows. Here, we investigate color-matching experiments with curved transparent objects for different matching stimuli: a uniform patch and a flat filter. Two instructions were tested: simply match the color of the glass object and the test element (patch and flat filter) or match the color of the dye that was used to tint the transparent object (patch). Observers’ matches differed from the mean, the most frequent, and the most saturated color of the transparent stimuli, whereas the brightest regions captured the chromaticity, but not the lightness, of patch matches. We applied four models from flat filter studies: the convergence model, the ratios of either the means (RMC) or standard deviations (RSD) of cone excitations, and a robust ratio model. The original convergence model does not fully generalize but does not perform poorly, and with modifications, we find that curved transparent objects cause a convergence of filtered colors toward a point in color space, similar to flat filters. Considering that, the RMC and robust ratio models generalized more than the RSD, with the RMC performing best across the stimuli we tested. We conclude that the RMC is probably the strongest factor for determining the color. The RSD seems instead to be related to the perceived “clarity” of glass objects.

## Introduction

Transparent objects allow some light to pass through their bodies, as opposed to opaque objects, which only absorb and reflect light. The conditions that lead to the perception of flat transparent filters and the factors that determine the perceived color of a flat filter have been extensively studied. Helmholtz (1867) and Koffka (1935) wrote about the perception of transparency in their books, where Helmholtz described the percept of transparency as “a transparent colored veil … spread over the field” and Koffka referred to it as “color scission,” in which the visual system can assign more than one color to the same image region, effectively splitting that region into layers and perceiving a colored filter overlaying another colored object. However, it was Metelli's work (1970, 1974) that has had a more lasting impact on the study of perceived transparency, resulting in the classical Metelli or episcotister model of transparency, whose original equation is coincidentally used in computer graphics in a form of alpha blending known as Porter and Duff's “over” operator (Porter & Duff, 1984; Foley et al., 1990), although the “over” operator actually models the effect of an opaque surface with partial coverage, whose optical effects are physically different from the optical effects of transparent objects (McGuire, 2019). We consider this in more detail later.

The episcotister is a device that Metelli used that rotates a sliced disk in front of a complete and stationary disk. In some of his experiments, the stationary disk was painted such that the left half had one gray value and the right half had another, creating a vertical split in luminance down the middle (a bipartite field) that the sliced rotating disk overlaid. The sliced rotating disk was painted with one uniform gray value. When the sliced disk is spun fast enough, optical mixing of the reflected light leads to the appearance of a solid, non rotating transparent filter in front of the bipartite field. Metelli determined a relationship between the reflectances of the fields of the two disks that predicts the amount of perceived transparency. Building on this, Beck et al. (1984) suggested that it is rather the relationships among the lightness percepts, instead of luminance or reflectance, that determine perceived transparency, which Metelli later adopted (Metelli, 1985). In fact, for the episcotister stimulus, whether a surface will appear transparent or not can be predicted by the relative luminance relationships between adjacent patches, without regard to their absolute luminance (Adelson & Anandan, 1990; Anderson, 1997). In the episcotister model, these relationships must occur at the well-known X-junctions, where they determine perceived depth order (Metelli, 1974; Beck et al., 1984; Brill, 1984; Adelson & Anandan, 1990; Gerbino et al., 1990; Kersten, 1991; Nakayama et al., 1990; Plummer & Ramachandran, 1993; Anderson, 1997; Masin, 2006; Singh & Anderson, 2002; Koenderink et al., 2008, 2010; Delogu et al., 2010). However, the episcotister model is not predictive of changes in transparency perception as other factors of the stimulus are varied, such as the mean luminance. For example, Singh and Anderson (2002) showed that perceived transparency varies with changes in mean luminance, which Metelli's model says should not happen, and they concluded that it was rather Michelson contrast that determined perceived transparency, although it has been suggested that Singh and Anderson's model does not incorporate the psychophysical “principle of independence of effects” for flat filters (Masin, 2006). Regardless, there are situations that give rise to the perception of transparency when the established luminance relationships at X-junctions are broken. Plus, transparency is even perceived without X-junctions (Watanabe & Cavanagh, 1993; Sayim & Cavanagh, 2011), so the episcotister model of transparency perception has seen less application in modern perceptual transparency research, although the episcotister itself continues to be used as a stimulus in studies.

All of the episcotister studies mentioned above were done in the achromatic domain. If the scene and filters are chromatic, then D’Zmura et al. (1997) found that if one considers the pixels in some extended and continuous region of a Mondrian and one then shifts the chromaticity of all of those pixels in the same direction and by the same amount, then one will perceive a flat colored transparent filter overlying the Mondrian. A similar effect is obtained when forcing the chromaticity of those pixels to converge on the same color and a mixture of both techniques also works. This effect is especially curious, since transparent objects will always reduce the luminance in the region of the image that they filter, relative to when the transparency is absent, since unless they are perfectly transparent, they will always absorb and/or reflect some of the incident light. However, the technique of D’Zmura et al. (1997) can be applied so that it does not change luminance, meaning the perceived transparency that their technique can generate is physically impossible (if only chroma is altered) (D’Zmura et al., 1997; Chen & D’Zmura, 1998; D’Zmura et al., 2000). Regardless, their model is predictive of the colors that observers set when they can adjust the color in a restricted portion of a filter (Chen & D’Zmura, 1998) and it is also predictive of the colors that observers set when they can adjust the color of surfaces seen through the filter (D’Zmura et al., 2000). It should be kept in mind, though, that the convergence model is a description of how the physical input to the visual system is altered by the presence of a colored filter. Although the original authors developed a computer vision algorithm to extract the convergence transform from images of flat filters (D’Zmura et al., 1997), they did not conclude that the human visual system was implementing that specific computer vision algorithm.

The flat filter settings that are consistent with the convergence model show that a physical regularity exists that the brain could utilize for assigning a color to a transparent object. If the convergence model holds for the stimuli in our article (considered in more detail later), then the question is how observers monitor or extract the magnitude and point of convergence from the image. While the computer vision form of the convergence model (D’Zmura et al., 1997) could potentially be implemented by the brain, there are also other established models of perceptual transparency that could generalize from the flat filter case to the curved glass case. For example, there are more recent attempts to investigate perceptual transparency with a physically based model (Allen, 1980; Nakauchi et al., 1999). The physically based model includes internal refraction and filtering effects on luminance. The studies based on this model have followed two closely related paths that have come to somewhat different conclusions. The investigations of Khang, Robilotto, and Zaidi (Khang & Zaidi, 2002a, 2002b; Robilotto et al., 2002; Robilotto & Zaidi, 2004) have found that observers use a measure of contrast to detect and match a flat filter (Robilotto & Zaidi, 2004; Robilotto et al., 2002; Khang & Zaidi, 2002a), since the mean color in the filtered region is insufficient to predict observer matches. In fact, when asked to match the color of two filters, each placed under a different illuminant, observers match the ratio of mean cone excitations between the filtered and unfiltered region (RMC) (Khang & Zaidi, 2002a). Khang and Zaidi (2002a) also found that observers do not match the chromaticity difference between filtered and unfiltered regions, but they do match the ratio of mean chromatic contrast. Here, we focus on the RMC that directly involves cone activations, since this is more directly comparable with other research described below. The ratios of mean cone excitations are similar to, but not exactly the same as, mean spatial cone excitation ratios (MCERs), which have been extensively studied with respect to color constancy (Foster & Nascimento, 1994; Nascimento & Foster, 1997, 2000, 2001; Foster et al., 2006, 2016). Actually, the work of Westland and Ripamonti (2000) and Ripamonti and Westland (2003) found MCER capable of predicting the strength of perceived transparency. Essentially, Khang, Robilotto, and Zaidi's investigations have found that observers are capable of making veridical identifications of filters across different illuminants and backgrounds (Khang & Zaidi, 2002b), achieving color constancy, and that observers match a quantity that is related to spatial variation and the change in cone signals between the filtered and unfiltered regions. From this perspective, flat filters act similarly to spotlights (Khang & Zaidi, 2004; Dojat et al., 2006; Knoblauch & Dojat, 2003). On the other hand, some investigations (Faul & Ekroll, 2002, 2011, 2012; Faul & Falkenberg, 2015; Faul, 2017) made a detailed investigation of the same physical model (Allen, 1980; Nakauchi et al., 1999) and devised a way to transfrom the physical parameters of the model into various perceptual quantities, such as the hue and saturation of the filter, provided that certain simplifying assumptions are adopted (Faul & Ekroll, 2011). Their model suggested that observers could either extract the ratio of the standard deviations of cone excitations between the filtered and unfiltered regions (RSD) as a measure of the flat filter's color code or they could use a more robust procedure that makes use of additional equations derived from the flat filter image generation process (which we refer to as the “robust ratio model” for reasons that become clear in the “Image statistics” subsection of the “Method” section). Their psychophysical experiments supported the conclusion that observers at least use the RSD, if not the more general and robust ratio model, but in the majority of their work, they use the RSD model. Using their perceptual model of filter color, they also suggested that when matching filters (each placed under a different illuminant), observers make a match that lies approximately halfway between a proximal appearance match and a perfect color constancy match (Faul & Falkenberg, 2015).

However, while the results of studies with flat filters have certainly provided insight into the potential mechanisms that underlie transparent color perception, they do not incorporate other factors that arise when considering curved, transparent objects. This is by no means to their detriment: The flat filter studies provide a solid foundation upon which to build further studies, and they have elucidated and tested many features that contribute to the perception of transparency. However, the RMC, RSD, and robust ratio models both do an adequate job of predicting the color of transparent filters, and they have not yet been applied to curved, transparent objects. Since both models are still viable, we tested their applicability to our stimuli to gain a better perspective. Essentially, the physical process of transparency in a variegated three-dimensional environment gives rise to shadows, caustics, specular highlights, subtle reflections of the surrounding environment, and a tinted and refracted (i.e., distorted) view of surfaces that lie beyond the transparent object's body. Shadows, caustics, specular highlights, and subtle reflections of the environment are not present in studies with flat filters, and all could play a role in the color appearance of a transparent object. For example, caustics are unique to specular reflections from glossy objects or refraction from more translucent or transparent objects and are rather complex, being determined by the geometry of the illumination, the shape of the object, the refractive index if the object is transmissive, and the shape of the diffuse surface that receives these focused regions of light (Lynch & Livingston, 1969; McGuire, 2019). Caustics may be too complex to serve as a cue to the structure of the illumination, but their color is heavily influenced by the transmission and absorption spectrum of the transmitting body, so they may play a role in the color appearance of the transmitting body.

We would like to briefly make additionally clear that we do not say that our Glaven stimuli are more natural than the flat filters. Both are natural and can be reproduced with real objects. Rather, the Glaven stimuli introduce additional factors that are also important aspects of physical transparency and that have perceptual counterparts. These factors serve as an additional “apparatus” when differentiating between various models and investigating potential mechanisms of perception. To that end, in this article, we investigate what information observers use when judging the color of curved, transparent objects. We present here variations of color-matching experiments that attempt to answer the question, “What is it that makes a red glass look red?” and we specifically test whether the RMC, the RSD, or the robust ratio models are capable of predicting observer matches with a flat filter.

It is also important to emphasize that we do not deal with the question of how transparent objects are detected. In this article, we are only focused on what color they have after the detection process is presumably complete. There could certainly be interactions and feedback between the detection mechanisms and those mechanisms that determine the color of glass, but that is outside the scope of this article. We make this clear, because if the RMC, the RSD, or the robust ratio model is not capable of predicting observers’ color matches, then this does not mean that they have no relevance whatsoever for transparent objects; it only means that the statistic does not play a role in the mechanism that assigns a color to a transparent object. So, even if a statistic does not predict the color match, it could still be used for detection of transparent objects or for qualifying the “clarity of glass” or some other feature of a transparent object.

## Method

### Scene rendering

**Figure 1.**
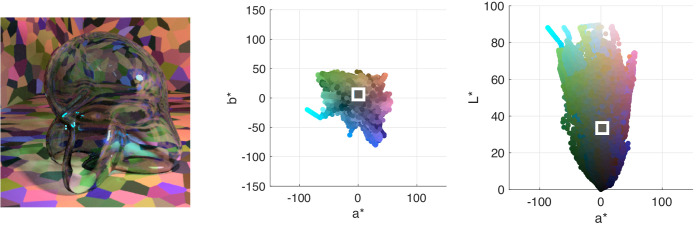
(Left) A close-up example of one of our stimuli: a glass Glaven with the blue Munsell high-transmission distribution under the white illumination. (Middle) The CIELAB coordinates in the (a*, b*) plane for the pixels from the filtered region of the image (i.e., the region covered by the Glaven). The points are colored with their corresponding RGB coordinates. The white box is centered on the average of the distribution. (Right) The CIELAB coordinates in the (a*, L*) plane for the pixels from the same filtered region of the image. The points are again colored with their corresponding RGB coordinates, and the white box is centered on the average of the distribution. Please be aware that because of compression artifacts that can distort images, especially dark images, we do not suggest using the images in this article as a way to double-check our analyses. The images in this article will also not look exactly as they did on our experimental setup, but if you are viewing the article on a handheld device, then tilting the device back and forth a bit can help make the images clearer, if necessary.

**Figure 2.**
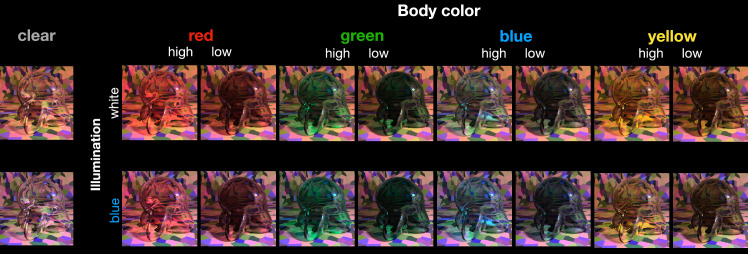
Our initial set of test images. The 16 images containing the red, green, blue, and yellow transparent Glavens were presented to observers during the main color-matching experiments. Two transmission levels were tested (high and low). The high-transmission condition was simply the original Munsell reflectance distribution that we gave to Mitsuba, and the low-transmission condition was made by scaling the distribution down by a constant factor of 0.68. The Glavens were rendered under two different illuminants: a blue and a white illuminant, each from the daylight locus. The two images at the left, showing the clear Glavens, illustrate that the illuminant can have a subtle effect on the color appearance of curved, transparent objects that are highly transmissive. See the text in the Method section for specific details about the rendering procedure.

To investigate the features that observers might be using when judging the color of curved transparent objects, we used the multispectral and physically based Mitsuba renderer (version 0.6.0, from Github; git commit: 06340ccbb3f4) to generate 400-pixel × 400-pixel (10.3${}^{\circ }$ visual angle square) images of a cube room with a glass object, called a Glaven (Phillips et al., 2009, 2016), and a small spherical light. The spherical light (not a point light source!) was placed above and to the right of the Glaven, outside the field of view of the camera, and it created a noticeable highlight on the top right of the Glaven. One example stimulus is in Figure 1, and more examples can be found in Figure 2. There are two things to notice. First, the Glaven is hollow inside, and the walls have a uniform thickness. We emphasize this because variations in thickness can look like variations in the transmission distribution, leading to ambiguity, which we did our best to avoid here. Second, upon close inspection, readers should be able to see two highlights overlapping each other. This is due to internal reflection between the inner and outer side of the wall of the Glaven. The highlight is tinged with the color of the transparent object, due to total internal reflection at the inner wall boundary, which is not something seen with glossy objects.

The three-dimensional (3-D) configuration of the scene was created in Blender (v2.79b) and was exported to Collada DAE format. The DAE file was then translated into a serialized binary file that Mitsuba can efficiently process, using Mitsuba's mtsutil program. The mtsutil program produces an XML file that specifies to Mitsuba the scene layout, the properties of objects in the scene, and the details of the simulated camera system that “takes the image” during the rendering process. The XML file was edited so that the Glaven had Mitsuba's smooth dielectric BSDF (bidirectional scattering distribution function) with the default properties that make it act like glass. We then used a small script, written in R (v3.6.1), to produce variations of this XML file that used different spectral distributions for the illumination, different transmission distributions for the dielectric BSDF, and to give the wall either a uniform Lambertian BSDF with a flat reflectance distribution (i.e., white walls) or to texture the wall with a multicolored RGB Voronoi pattern with a Lambertian BSDF. We also found that we could only achieve acceptable rendering of caustics with the small, spherical light source and with the Metropolis Light Transport integrator in combination with the Independent sampler running at 1,000 samples per pixel.

The color of the glass Glaven was varied by providing different Munsell reflectance distributions to the spectral transmission and spectral reflectance parameters of the dielectric BSDF. Four Munsell reflectance distributions were chosen from the database provided by the Computational Spectral Imaging group at the University of Eastern Finland (Hiltunen, 2019). They corresponded to the chips listed in Table 1.

**Table 1.** The chips whose reflectance distributions were used as the spectral transmittance and spectral reflectance distributions of the glass Glaven when it was rendered with Mitsuba.


Red	2.5R 7/2
Green	5G 2.5/2
Blue	5B 4/1
Yellow	10Y 5/1

**Figure 3.**
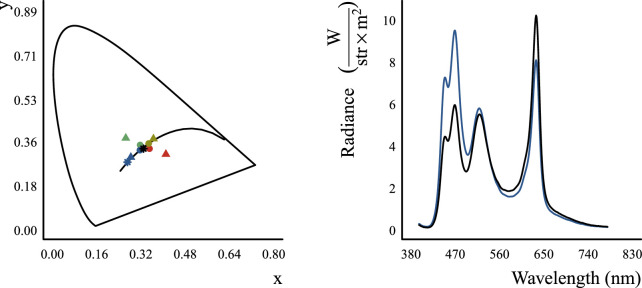
Left panel: Representation of illuminants, stimulus transmission distributions, and the matching filter distributions in the CIE1931 xyY chromaticity diagram. The colored stars denote the blue (blue star) and white (black star) illuminants that were given to Mitsuba when rendering our scenes. The colored disks denote the chromaticity coordinates of the four Munsell transmission distributions (red, green, blue, and yellow) that were used when rendering the Glavens. They correspond to a flat filter, lying on a white surface and imaged under a neutral illuminant. Since changing the intensity of a distribution does not change its position in the chromaticity diagram, the high- and low-transmission Munsell distributions plot directly on top of each other and so only four of the eight disks can be seen. The triangles denote the four Munsell distributions (red, green, blue, and yellow) that were used as endpoints of two filter transmission axes (red-green and blue-yellow). During experiments described in the “Experimental paradigm and stimulus presentation” section below, observers could vary the transmission distribution of a matching filter through linear combinations of the two filter transmission axes. Right panel: The spectral distributions corresponding to the two illuminants in the chromaticity diagram to the left. The blue distribution corresponds to the blue star and the black distribution corresponds to the black star.

The Munsell reflectances were chosen such that if they were used as the reflectance distributions for a piece of paper and these hypothetical papers were measured under an equal-energy white, then their chromaticity coordinates would lie close to the cardinal axes of the MacLeod-Boynton-Derrington-Krauskopf-Lennie color space (MB-DKL) (MacLeod & Boynton, 1979; Derrington et al., 1984). (Standard procedures for converting between linear RGB coordinates and the MB-DKL space are documented elsewhere; Zaidi & Halevy, 1993; Hansen & Gegenfurtner, 2013.) Stimuli with coordinates on these axes selectively stimulate the S-(L+M) and the L-M mechanisms (Derrington et al., 1984). We also made four more spectral distributions that were scaled versions (scaling factor = 0.68) of the Munsell reflectance distributions that were just described. These scaled versions, when used as the spectral transmission and spectral reflectance distributions of the glass BSDF, result in a darker and more saturated color appearance for the glass. Color coordinates for these Munsell reflectances and the illuminants (described below) in the CIE1931 xyY space are depicted in Figure 3.

For the multicolored Voronoi background, all eight of these spectral distributions were rendered under a blue and a white illuminant, giving 16 combinations in total. (Note that the images with the white walls were rendered under a neutral illuminant metameric to D65.) The spectral distributions of these illuminants were obtained from prior work that used hyperspectral measurements of real scenes (Ennis & Doerschner, 2019). Briefly, the illuminants were measured under a JUST Normlicht LED box by placing a PhotoResearch white reference in the box at approximately a 45${}^{\circ }$ angle and placing the line of sight of the hyperspectral camera roughly perpendicular to the white reference surface. The two chosen illuminants lie along the daylight locus and were created from linear combinations of the blue and yellow illuminants taken from the just referenced hyperspectral measurements. The spectral distributions of the illuminants were first scaled down by 50%, because otherwise, highlights would be burnt out and map to maximum white (i.e., all RGB values in the highlight would have been at 100%). These dimmer illuminants were then used in the next step: Each illuminant was slightly desaturated by scaling it down to 70% of its maximum and mixing it with 30% of the other distribution. For example, a relatively slightly desaturated blue illuminant was produced by scaling it down by 70% and adding the spectral distribution of the yellow illuminant after it was scaled down to 30% of its maximum. This was done because while highly saturated illuminants produce physically reasonable images, they will have a reduced variance of hues. The two resulting illuminants that were used for rendering were blue and white in appearance and were on the daylight locus. This was acceptable, since we were not performing a rigorous color constancy study and were mainly interested in having some extra variety in the stimuli.

Our version of Mitsuba was configured to render with multispectral data, rendering visible light in the range of 360 nm to 830 nm in 47 equally spaced wavelength bands. The distributions that we provided as input to Mitsuba were specified in the range of 380 nm to 830 nm and were sampled either in 400 equally spaced wavelength bands (Munsell reflectances) or in wavelength bands that were on average ∼1.12 nm wide (illuminant distributions), which is due to the construction of our hyperspectral camera. When provided with these data, Mitsuba interpolates them and resamples them at 360 nm to 830 nm in 47 equally spaced wavelength bands, filling in zeros where no data were provided by the user. This was more than sufficient for our purposes and produced realistic and pleasing images.

The Voronoi background seen in Figure 2 was produced with a modified version of an OpenGL fragment shader created by Íñigo Quílez and found in Quilez and Jeremias (2017). The Voronoi algorithm determined not only the shape of each element in the texture but also its color, which was sampled from multiplications of three base colors, where the weight of each base color was modulated according to a cosine that was sampled with the ID of each element, as determined by the Voronoi algorithm, and the specific trio of base colors was also determined by the ID as well. We wrote a small OpenGL-based program in Rust (v1.30) (Matsakis & Klock, 2014; The Rust Programming Language, 2017) that used the shader to produce a large 1,024-pixel × 1,024-pixel RGB Voronoi texture that could be fed into Mitsuba to act as a wall texture.

We also rendered the scene with a Lambertian background that had a flat reflectance distribution with 100% reflectance, giving the appearance of white walls. The uniformly reflecting Lambertian BSDF is a feature provided by Mitsuba, so we only had to specify the parameters in the XML scene file. We did not provide any external spectral data for the white walls. Note that the images with the white walls were rendered under a neutral illuminant that was metameric to D65. The reason for this is given at the beginning of the “Experimental paradigm and stimulus presentation” section below.

The final render output of Mitsuba, in our case, was an HDR OpenEXR image containing linear RGB data in 16-bit floating point format. Mitsuba uses the standard CIE1931 color-matching functions (Wyszecki & Stiles, 1982) and the standard sRGB specifications (Multimedia systems and equipment - Colour measurement and management - Part 2-1: Colour management - Default RGB colour space - sRGB, 1999) (in particular, the ITU-R Rec. BT. 709-3 primaries [Parameter values for the HDTV standards for production and international programme exchange, 1998] with a D65 white point) to convert from the multispectral render output to linear RGB coordinates. Mitsuba's built-in Reinhard tonemapper (Reinhard et al., 2002), provided by the mtsutil program, was used to convert these outputs to 8-bit RGB images in a PNG format. The following parameters were provided to the program: multiplier = 0.8, gamma = 1.8, key = 0.8, and burn = 0.1. All other parameters were left at their default values, and we found this combination to be best at preventing burn-out of highlights.

In total, for the main experiments described here, we had 24 images: 8 with the Voronoi background and the four original Munsell distributions (4 for each illuminant), 8 with the Voronoi background and the four scaled Munsell distributions, 4 with the white background and the four original Munsell distributions, and 4 with the white background and the four scaled Munsell distributions.

### Experimental paradigm and stimulus presentation

The task of observers was to change the color of a matching element until it appeared to have the same color as the glass Glaven shown in the test image. The Glaven stimulus and the matching element were always presented simultaneously. The Glaven stimulus was always positioned at the center of the monitor, and the matching element was always to the right of the Glaven stimulus. In the case of the uniform patch element, it was centered 7.72${}^{\circ }$ visual angle to the right, and in the case of the flat filter matching element, it was centered 8.75${}^{\circ }$ to the right, since it was larger than the uniform patch-matching element. The Voronoi background images and the white wall background images were tested in separate experiments. The white wall images were tested to see if observers perform some kind of color constancy-esque discounting operation when they make their matches. Essentially, is the color of a transparent object determined by what the object would look like if it were placed against a white background under a neutral illuminant?

**Figure 4.**
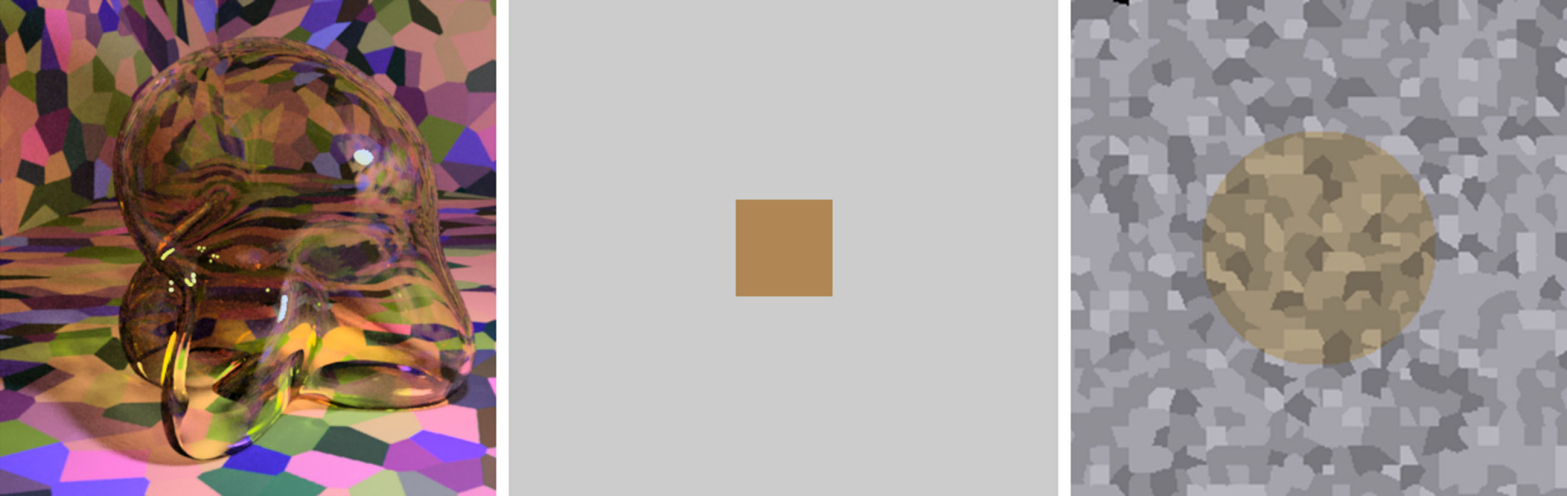
Examples of the stimuli that observers used when making a color match to the transparent Glaven. (A) An example stimulus to which an observer would make a match. (B) Observers could adjust the color of a simple patch. Shown is the average setting of observers in the “proximal match” experiment for the glass Glaven shown in Panel A. (C) Observers could adjust the color of a flat transparent filter, lying above an achromatic Voronoi background. Shown is the average setting of observers in our experiment for the glass Glaven shown in Panel A. See the text in the “Method” section for specific details about how the matching stimuli were created and how observers controlled the color of the matching stimuli.

Prior to each experiment, observers were shown an example stimulus and the respective matching element and told that they were to “change the color of the matching element on the right until it had the same color as the glass object in the scene on the left.” These instructions changed slightly in an experimental variation that is described below. The observers were then shown how moving the mouse and pressing buttons would change the color of the matching element (details also below about how the mouse changed the color of the matching element). Observers then did a practice trial, where they were given no feedback. We then asked if everything made sense, and no observers expressed difficulty or confusion about the stimuli or their task in any of the experimental variations. They then adapted to a light gray background ($∼82\frac{cd}{m^{2}}$) for 1 min. A beep then indicated the start of the experiment, and one of the images corresponding to the specific test set (Voronoi or white wall) was presented at the center of the monitor. The images and the matching elements were always presented against the same light gray background that was used for adaptation, and the images were always presented in a randomized order. Five matches were made for each image, resulting in 80 trials for the Voronoi set of stimuli and 40 for the white wall set of stimuli. We used two different matching elements in separate experiments: a 50-pixel × 50-pixel (1.3${}^{\circ }$ visual angle square) uniform patch or a 256-pixel × 256-pixel (6.6${}^{\circ }$ visual angle square) image of a flat transparent filter lying above an achromatic Voronoi background. The flat transparent filter was rendered according to an equation provided by Khang and Zaidi (2002a) that is similar in result to the formulation given by Faul and Ekroll (2002, 2011, 2012; Faul & Falkenberg, 2015). See Figure 4 for examples of the matching elements. The Voronoi background was generated using the same Rust program that was used to make the Voronoi background in the rendered scenes, but it was altered to only produce various shades of gray. The resulting Voronoi background texture was 256 pixels × 256 pixels.

Note that while the uniform patch does not look transparent, this did not invalidate its use as a matching element. As will be seen in the results, observers make consistent matches that correspond with one's perceptual expectations, and due to its simplicity relative to a flat filter, it becomes a potential probe of the features that are most essential for the color of a transparent object. For example, see Giesel and Gegenfurtner (2010) for experiments that determine important properties of perception for curved opaque objects with a uniform patch. We do not intend to say with this that the “color” of the uniform patch is equivalent to the “color” of glass. Colors of different material classes most likely exist in their own separate perceptual spaces (Beck, 1972; Katz, 1911; Xiao & Brainard, 2008). What we instead are testing is if observers can make a mapping between transparent colors and uniform Lambertian colors, and presumably, this mapping will incorporate features that are important for determining the color of glass. On the other hand, the flat filter actually appears transparent and has additional complexity that is reflected in additional scene statistics and sources of information that the visual system can monitor when making a color match. With both together, one can get an idea of the most important stimulus features and then “close the gap” in terms of variance explained with the additional information provided by more complex matching elements. Put another way, the uniform patch settings are interesting, because they provide a partial answer to the question of what “the color of a transparent object” means. Can the color of the glass be abstracted and conceived of as something separable or is it absolutely necessary that the color be “embedded” in a transparent matching element for the task to be feasible and sensible? Is the color an intrinsic and constant property of the glass, or is it actually dependent on specular highlights, reflections, shadows, caustics, and so on? Can one find a piece of paper that has the same color as a piece of glass? (Similar questions were considered by Wittgenstein [1978].) Since the uniform patch does not have any of these additional elements, it forces the observer to make a choice about what is most relevant. And, if observers make settings that consistently deviate from the flat filter in many respects, then these “errors” could serve as an additional apparatus for understanding the relevant perceptual mechanisms. Lastly, the uniform patch-matching element is a tool that has been used in countless color vision studies, and we wanted to acknowledge and build on a robust tradition of starting simple and building upward in complexity.

We tested two different sets of instructions for the uniform patch-matching element in separate experiments and one set of instructions for the flat transparent filter in an additional experiment. In the first experiment with the uniform patch, observers were asked to adjust the color of the patch until it appeared to have the same color as the glass Glaven (“proximal match”). This was similar to a proximal stimulus match in color constancy research. In a follow-up experiment, observers were asked to adjust the color of the patch until it appeared to have the same dye that was used to tint the glass Glaven (“dye match”). This was similar to asking observers to make a paper match in color constancy experiments (Arend & Reeves, 1986).^1^ In the flat transparent filter experiments, observers were asked to “change the color of the glass filter until it appeared to have the same color as the glass Glaven.”

In all experiments, observers adjusted the color of the matching element by moving the mouse. The mouse cursor was hidden from view during the experiment. As an example of how mouse position was mapped to color, we can consider the uniform patch stimulus, where its color was specified in the MB-DKL space. When the position of the cursor was at the center of the screen, the uniform patch was mid-gray. When the cursor was moved left, the color of the patch was shifted a proportional amount along the green direction of the L-M cardinal axis of the MB-DKL space. When the cursor was moved right, the color of the patch was shifted a proportional amount along the red direction of the L-M cardinal axis. Up and down were similarly mapped to the blue and yellow directions of the S-(L+M) cardinal axis. With this setup, moving the mouse around in a circle at a given radius from the center of the screen would modulate the uniform patch through all colors on a hue circle at a proportional radius in MB-DKL space. The lightness of the patch could be adjusted by pressing the left mouse button to make it darker and the right mouse button to make it brighter.

The normalized mouse position was computed by subtracting the mouse coordinates for the center of the monitor from the current mouse position and dividing the x-component by half the width of the monitor (specified in pixels) and the y-component by half the height of the monitor (specified in pixels).

In the case of the flat transparent filter, the achromatic Voronoi image was loaded into MATLAB, and a circular region at the center of the image with a radius of 60 pixels was processed according to the filter formula in Khang and Zaidi (2002a). Mouse position was now mapped to linear combinations of four Munsell reflectance distributions (see Figure 3) that defined the transmission distribution component of the filter equation. These four Munsell distributions were also taken from the Computational Spectral Imaging group at the University of Eastern Finland (Hiltunen, 2019), and they corresponded to the chips listed in Table 2.

**Table 2.** The Munsell chips whose reflectance distributions were used in the observer-controlled linear combination that determined the spectral transmittance distribution of the flat filter matching element.


Red	7.5RP 6/8
Green	10G 7/8
Blue	7.5B 2.5/2
Yellow	7.5Y 5/2

These four Munsell distributions were all normalized, such that their maxima were equal to 1, and they were taken in pairs as the endpoints of two axes, one pair defining a “red-green” variation and the other defining an orthogonal “blue-yellow” variation. Now, moving the mouse would vary the shape of the transmission distribution of the filter, and clicking the mouse buttons would vary the overall transmittance, which would change the apparent opacity or “lightness” of the filter. The changes were enacted according to the following equation:

$$\begin{array}{ccc} & & \;\;\;\;\;\;\;\;\;FilterTransmission=Thickness*((NMX*RF\\ & & +\;(1-NMX)*GF))+\;(NMY*BF\\ & & +\;(1-NMY)*YF)), \end{array}$$

where Thickness was increased or decreased according to left and right mouse clicks, respectively; RF stands for the red filter transmission distribution; GF is for the green filter; BF is for the blue filter; YF is for the yellow filter; and NMX and NMY stand for normalized x- or y-mouse position, where being completely in the top-left corner of the screen would be NMX=NMY=0 and the bottom right of the screen would be NMX=NMY=1. This scheme produces a transmission distribution color space that is analogous to the MB-DKL color space, and moving the mouse in a circle at a given radius from the center of the monitor would modulate the color of the filter through all the hues.

In all experiments, the stimuli and matching element remained on the screen until observers finished making their match. When observers were satisfied with their match, pressing the middle mouse button would clear the screen, save their data, and prepare the next trial. Before each trial, observers readapted to the mid-gray of the monitor for 2 s.

### Monitors

Our experimental setups were contained in one room, with the walls painted black, and the lights were always off during the course of an experiment. The only light came from the monitor for the experiment. Stimuli for the Voronoi background stimuli (“dye match,” “proximal match,” and “filter match”) were displayed on a 10-bit EIZO ColorEdge CG245W monitor (EIZO Corporation, Hakusan, Japan) using Psychtoolbox (SVN revision 8643) (Brainard, 1997; Pelli, 1997; Kleiner et al., 2007) via the MATLAB environment, R2014b (MathWorks, Inc.; Natick, MA, USA). The computer that was connected to the EIZO was a Dell Precision T1700, running Microsoft Windows 7 Professional edition SP1 (64-bit) (Microsoft Corporation, Redmond, WA, USA) with an Nvidia Quadro K620 graphics card (Nvidia Corporation, Santa Clara, CA, USA) controlled by Version 347.52 of the Nvidia drivers.

For the experiment with the white walls (filter match), stimuli were displayed on a SONY PVM2541-A OLED (Sony Corporation, Tokyo, Japan) via Psychtoolbox (SVN revision 9641) (Brainard, 1997; Pelli, 1997; Kleiner et al., 2007) using the MATLAB environment, R2018b (MathWorks, Inc.). Although the SONY OLED is a 10-bit monitor, it requires special hardware to be used in this mode, so our SONY OLED was running in 8-bit mode, but this was found to not have an effect on the final results of our experiments. The computer that was connected to the SONY OLED was an HP Pavilion 595 (HP, Inc., Palo Alto, CA, USA), running Microsoft Windows 10 Home edition (64-bit) with an Nvidia Geforce GTX 1050 Ti controlled by Version 398.36 of the Nvidia drivers.

Both computers were disconnected from the Internet to prevent automatic updates from potentially changing their behavior. Both monitors were calibrated using a Konica-Minolta CS2000-A via standard procedures documented elsewhere (Zaidi & Halevy, 1993; Hansen & Gegenfurtner, 2013a). In particular, the calibrations were used to ensure that our stimuli could be accurately reproduced by the gamuts of both monitors and to calculate CIELAB (Recommendations on Uniform Color Spaces, Color-Difference Equations, Psychometric Color Terms, 1978; Wyszecki & Stiles, 1982) representations, and LMS cone excitations (Stockman et al., 1999; Stockman & Sharpe, 2000) for our stimuli (explained in further detail below, in the “Color space conversion” subsection). The properties of the Eizo monitor in the CIE1931 xyY color space were as follows: red phosphor (x: 0.6733, y: 0.3088, Y: 37.223), green phosphor (x: 0.2103, y: 0.6869, Y: 86.659), and blue phosphor (x: 0.1564, y: 0.0554, Y: 7.9206), and the luminance of its mid-gray was 62.29 $\frac{cd}{m^{2}}$. The properties of the SONY OLED monitor in the CIE1931 xyY color space were as follows: red phosphor (x: 0.6694, y: 0.3235, Y: 37.675), green phosphor (x: 0.1924, y: 0.719, Y: 82.7), and blue phosphor (x: 0.1436, y: 0.0539, Y: 9.6269), and the luminance of its mid-gray was 66.37 $\frac{cd}{m^{2}}$.

### Observers

We had different groups of observers in each of the experiments: six that adjusted the uniform patch with the “proximal match” instructions and the multicolored Voronoi background, nine that adjusted the uniform patch with the “dye match” instructions and the multicolored Voronoi background, five that adjusted the flat transparent filter and the multicolored Voronoi background, and seven that adjusted the flat transparent filter and the white walls. Three observers also ran in a control experiment discussed in more detail later in this article.

All observers were in the age range of 20–30 years, so yellowing of the crystalline lens cannot be considered a significant contribution to our results, and they were all recruited from the student population at the Justus-Liebig University in Giessen. The observers were naive to the purpose of the experiment. All observers had normal or corrected-to-normal visual acuity. All observers were checked for color deficiency using the Isihara color plates (Ishihara, 1973) and no observers were excluded based on its criteria. Observers were paid for their participation in the experiments. All observers gave written informed consent in accordance with the Code of Ethics of the World Medical Association (Declaration of Helsinki) for experiments involving humans. The experiments were approved by the local ethics committee LEK 2015-0021.

### Analysis

#### Color space conversion

For analysis, we converted our rendered images and the settings that observers made with the matching elements to the CIELAB color space (Recommendations on Uniform Color Spaces, Color-Difference Equations, Psychometric Color Terms, 1978; Wyszecki & Stiles, 1982) and LMS cone excitations (Stockman et al., 1999; Stockman & Sharpe, 2000). The conversions were done with routines written in the Go programming language (v1.13.3) (The Go Programming Language, 2019). Essentially, the routines convert the linearized RGB values at each pixel to the corresponding CIELAB or LMS coordinates, using the equations described below, and return a CIELAB “image” or LMS “image,” respectively.

The CIELAB color space was developed to be a perceptually uniform color space, meaning that movement in any direction at any point in the space by a unit amount should correspond to a step size of 1 JND. One feature of the space that assists with this goal is accounting for adaptation to the illuminant, whose CIE1931 XYZ coordinates play a role in the transformation equations that define the CIELAB space. While the original CIELAB definition does not achieve the goal of complete perceptual uniformity (Ennis & Zaidi, 2019), it is close enough for our purposes here. Also, while the CIELAB color space was designed for reflective materials, we only use it to have some way to represent the data of our observers.

The CIELAB space has three color axes that correspond to red-green, blue-yellow, and light-dark variations. The equations that map a color to the CIELAB space require the CIE1931 XYZ coordinates of the color and the CIE1931 XYZ coordinates of the illuminant (also known as the “reference white point”). The equations are as follows:

$$\begin{array}{ccc} L^{*} & = & 116*f\left(\frac{Y}{Y_{n}}\right)-16\\ a^{*} & = & 500*\left(f\left(\frac{X}{X_{n}}\right)-f\left(\frac{Y}{Y_{n}}\right)\right)\\ b^{*} & = & 200*\left(f\left(\frac{Y}{Y_{n}}\right)-f\left(\frac{Z}{Z_{n}}\right)\right) \end{array}$$

and

$$\begin{array}{ccc} f(t) & \;= & \left\{\begin{array}{cc} \sqrt[{3}]{t}, & \text{if}\;t>\delta^{3}\\ \frac{t}{3\delta^{2}}+\frac{4}{29}, & \text{otherwise} \end{array}\right.\\ \delta & \;= & \frac{6}{29} \end{array}$$

where $X_{n}$, $Y_{n}$, and $Z_{n}$ are the CIE1931 XYZ coordinates of the reference white point and X, Y, and Z are the CIE1931 XYZ coordinates of the color of interest. L* aims to be a perceptually uniform scale for lightness, a* aims to be a perceptually uniform scale for red-green variations, and b* aims to be a perceptually uniform scale for blue-yellow variations.

When converting our images to the CIELAB color space, the chromaticity coordinates of the maximum white of the monitor were used as the reference white point, for which there is already a precedent in color vision research (Milojevic et al., 2018). This means that an RGB value of [1,1,1] will have an L* value of 100 and an a*=b*=0 as chromaticity components.

The LMS excitations were computed using the calibration data of the monitors. Briefly, an {L,M,S} triplet can be computed from an {R,G,B} triplet in the range of $\left[0,1\right]$ with knowledge of the spectral distributions emitted from the three primaries when they are at their maximum intensity. Once these were obtained, we used the $2^{\circ }$ LMS cone spectral sensitivity functions (Stockman et al., 1999; Stockman & Sharpe, 2000) to calculate the {L,M,S} excitations for the three primaries. Then, provided that the primaries are linearly independent and do not change in their properties as their intensity changes, one can take a given {R,G,B} triplet and scale and sum the maximum {L,M,S} excitations accordingly to get the total {L,M,S} excitation. For example, if the {R,G,B} triplet ={0.5,0.2,0.5}, then the total {L,M,S} excitation $=\{0.5\cdot \left(L_{R}+L_{G}+L_{B}\right),0.2\cdot \left(M_{R}+M_{G}+M_{B}\right),0.5\cdot \left(S_{R}+S_{G}+S_{B}\right)\}$.

#### Convergence model analysis

We applied the convergence model to our stimuli to see if the physical regularities that it captures for flat filters carry over to curved transparent objects. We examined the convergence model's effects in the MB-DKL color space (MacLeod & Boynton, 1979; Derrington et al., 1984), just like in the most recent convergence model report (D’Zmura et al., 2000), as well as in the LMS and CIELAB color spaces. See the “Color space conversion” and “Scene rendering” sections for more details about these spaces.

**Figure 5.**
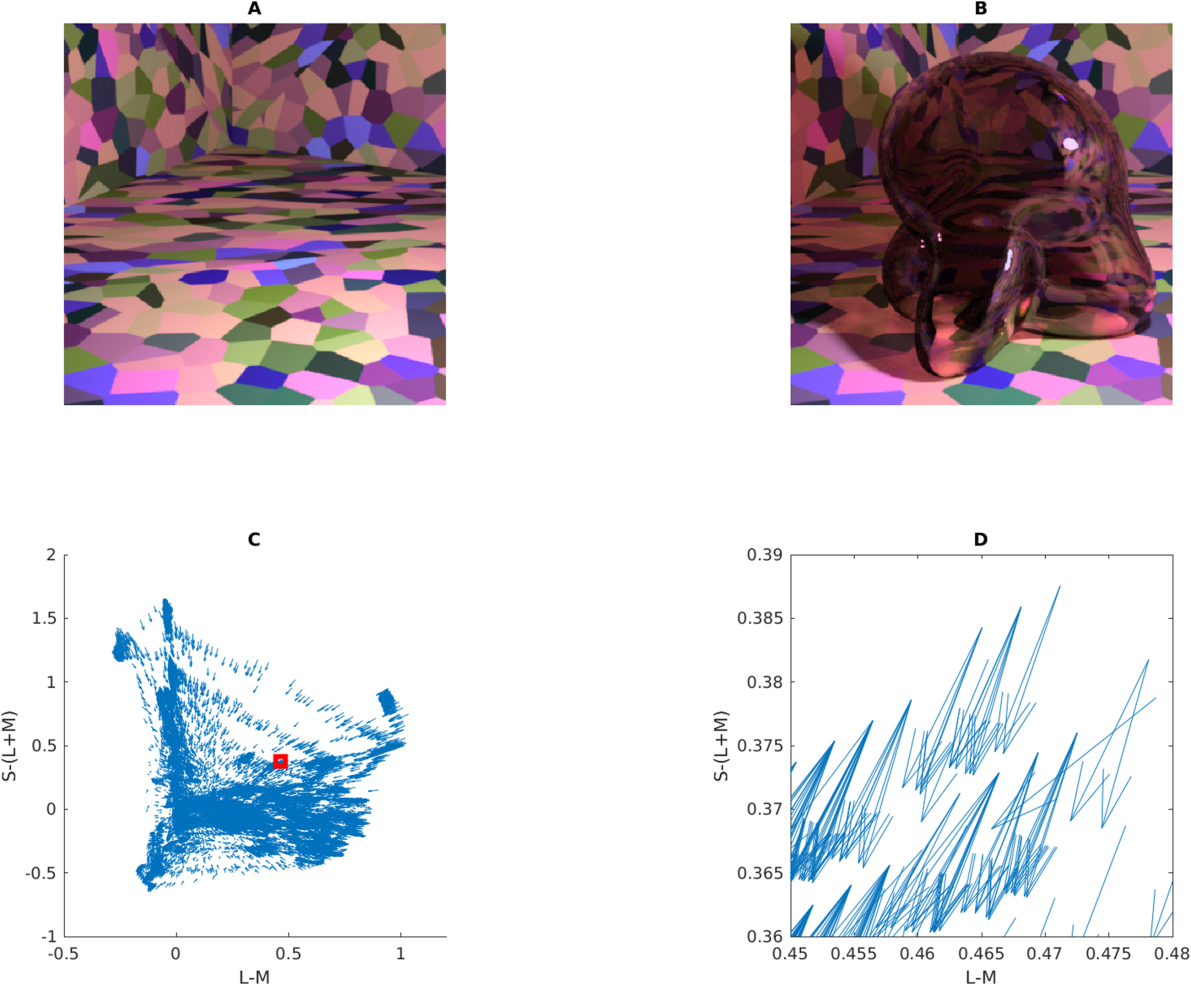
Details of our application of the convergence model to our stimuli. (A) One of the main scenes tested in this article, but with the glass Glaven removed. (B) One of the actual stimuli used in the article: a colored, curved glass object (a Glaven), placed in the room from Panel A. The convergence model focuses on analyzing the change in color induced by the glass Glaven in the region that it filters. (C) The vector field that connects the color of pixels in Panel A to their filtered counterparts in Panel B (vector lengths have been arbitrarily scaled to aid visibility and show the general pattern). Only the pixels that are actually filtered by the glass Glaven are included in the vector field. The bases of vectors are the colors from Panel A, and the tips are their filtered counterparts from Panel B. (D) A close-up zoom of the region marked by the red square in Panel C. One can see that many vectors are assigned to the same point in color space. For presentation purposes, the scaling of the vectors is arbitrary, since we plotted the data with MATLAB's quiver() function.

Consider Figure 5. In Panel A, we show the multicolored room that was frequently rendered for the stimuli in this article. Panel B shows how this scene looks when a glass Glaven is added. We considered two ways of interpreting and applying the convergence model: 1) (a) as the Affine model considered in the original papers and 2) (b) in terms of the vector field that is formed by connecting the unfiltered color at a pixel (before the Glaven is in the scene; vector tail) to the filtered color at the same exact pixel (after the Glaven is added to the scene; vector tip) (Panel C). In the case of the Affine model, we also tested applying it to the full-color distribution (i.e., luminance/lightness and chroma), as well as just to the isoluminant distribution (i.e., chroma only; after projecting all colors into the isoluminant plane). The reasoning for this is that the convergence model can be used to produce percepts of objects that transmit light when only altering the chroma of colors, without altering the luminance, which is physically impossible for a flat filter that is lying flat on a surface (filters always absorb some light, so they must always reduce luminance, unless they are completely clear). However, it could have been the case that for the richer, more complex stimuli that we investigate here, the mixed effects of reflections, highlights, and caustics could augment the luminance-reducing effects of our glass Glavens, leading to a breakdown of convergence in the luminance dimension, but with convergence still happening in the chromatic dimensions, providing some foundation for the perception of the physically impossible flat filters that the original convergence papers investigated. It could also be the case that observers mainly monitor convergence for chromaticity, but use some other mechanism for determining the “lightness” of a transparent object. Since both were a priori unclear, we decided to test it for the sake of completeness.

Returning to Figure 5 and the application of the Affine model, Panel C depicts a vector field that shows how the colors from the region filtered by the glass Glaven are transformed when going from the image in Panel A to the image in Panel B. The bases of the vectors correspond to the colors from the image in Panel A, and the tips correspond to the colors from the same pixels after they have been filtered by the Glaven in Panel B. Panel D shows a close-up zoom of a region in the center of Panel C. It details how the vector field that characterizes the color change between the filtered and nonfiltered scenes can have many vectors assigned to the same point in the chromaticity plane.

In particular, we investigated the most general 12-parameter Affine form of the original convergence model and the more restrictive 4-parameter model that D’Zmura et al., 2000 accepted, because if the more restrictive model fails, then we can see if the more general Affine form is applicable. Both of these models sit in a nested hierarchy that D’Zmura and colleagues tested, with the most general 12-parameter Affine form at the top. The 4-parameter form is the 12-parameter form, but with select parameters fixed. To be clear, the 12-parameter form can account for shearing, projection, translation, rotation, and arbitrary and independent scaling of the components of the filtered color distribution, whereas the 4-parameter form can only account for translation and arbitrary, but uniform, scaling of the components (i.e., the same scaling factor is applied to all components). The equation for the 12-parameter general Affine form (D’Zmura et al., 2000) is

$$\begin{array}{c} b=Ma+t, \end{array}$$

where a is a color before being filtered (e.g., one of the colors from Panel A of Figure 5), M is a 3 × 3 matrix, t is a 3 × 1 translation vector, and b is the resultant color after filtering (e.g., one of the colors from Panel B of Figure 5). For the 4-parameter restricted Affine form (D’Zmura et al., 2000), the equation is

$$\begin{array}{c} b=\beta a+t, \end{array}$$

where a, b and t are defined as before, but β is now a scalar (i.e., a single real number that uniformly scales all components of the color vector, a).

For this analysis, we performed the following procedure in MATLAB (R2018b; MathWorks, Inc.):

(1) Use the same mask for both images to extract the pixels that are directly filtered by the transparent object.
(2) Convert the RGB colors of these pixels to the 3-D LMS, MB-DKL, and CIELAB color spaces.
(3) If testing the 12-parameter model, then start with a random 3 × 3 linear transformation matrix (M) and 3 × 1 translation vector (t) and apply them to the unfiltered colors (a) in the respective color space (a separate linear mapping and translation vector was found for each color space). If instead testing the 4-parameter model, then start with a scaling factor (β) and a 3 × 1 translation vector (t). The scaling factor was used to uniformly scale all components of each color, and the translation vector was used to shift them. The combined effect of the transformation matrix or scaling factor and the translation vector is an Affine mapping.
(4) Then, calculate the root mean squared error (RMSE) between the actual filtered colors in the masked region in Panel B of Figure 5 and the results of applying the respective transformation to the unfiltered colors.
(5) Use the RMSE in conjunction with the fminsearch routine of MATLAB (an iterative simplex search algorithm) to find the transformation pair (matrix/scaling factor and translation vector) that best maps the unfiltered colors from the masked region of the image in Panel A of Figure 5 to their filtered counterparts from the image in Panel B.
(6) Separately repeat the Affine transformation search for the MB-DKL and CIELAB spaces, but ignoring luminance (in MB-DKL) or lightness (in CIELAB) (i.e., all colors are projected into the mid-gray isoluminant plane, and only chroma is considered). This emulates part of the original investigations of the convergence model. We did not perform this step for the LMS space, since declaring a luminance axis turns it into a quasi-MB-DKL space, which is already being tested.

We did this analysis for three different types of mask, for three refraction levels, and with or without darker pixels included in the analysis. The reason for testing different refraction levels is that the convergence model depends on each unfiltered color being properly paired with its filtered counterpart. Since refraction distorts this relationship by bending the light (which can be seen most clearly at the edges of the glass Glaven), we tested how much of an influence it can have. The different refraction levels were obtained by changing the interior index of refraction (IOR) parameter for the glass Glaven in the Mitsuba rendering engine that we used, while the exterior IOR was always locked to its default value of 1.000277 (i.e., the IOR of air). Briefly, indices of refraction control how much incoming light rays are “bent” when they pass through the surface of a light-transmitting object. The amount of bending depends simultaneously on the index of refraction of the light-transmitting object and that of the surrounding medium. If the interior and exterior IOR are equal, then the light is not bent. The following values for interior IOR were tested: interior IOR = 1.000277 (“no refraction” condition), interior IOR = 1.02 (“little refraction condition”; as low as we could go before it looked like no refraction was happening), and interior IOR = 1.5046 (“normal refraction” condition; Mitsuba's default when rendering glass and was used for all stimuli tested later in this article).

For the three masks, we tried the following: one that captured all pixels filtered by the transparent object, one that excluded the specular highlights from the calculations, and one that excluded all specular reflections from the calculations. The specular reflections were found by putting the specular reflection component of the transparency BSDF in Mitsuba (the physically based renderer we used; see “Method”) to its maximum value and putting the transparency component to zero (i.e., full absorption of all incoming light, giving a black, but shiny, surface).

When excluding darker pixels in the convergence analysis, we set a threshold of 5% of the maximum luminance within the Glaven (excluding the specular highlights when computing this threshold). This is quite a strict threshold that only allows pixels that have visibly clear colors into the analysis. The reason for testing the effect of dark pixels was that dark pixels will contribute a small signal and so any noise could potentially induce large deviations in chroma from dark pixel to dark pixel that will lead to spurious distortions of the associated vector field.

We also wish to point out that we additionally tested the influence of the relative scaling of the Stockman–Sharpe LMS absorption curves (Stockman et al., 1999; Stockman & Sharpe, 2000) on the fminsearch procedure in MATLAB. For instance, one could use the original, unmodified Stockman–Sharpe LMS absorption curves, but one can also precondition them by normalizing their responses to an equal energy white stimulus of unit intensity. When one performs this pre-conditioning step, the final results of the fitting procedure for the 12-parameter form of the convergence model differed by roughly 5% on average. We presume that since the fminsearch procedure was allowed to independently scale the three cone channels during its search in the 12-parameter case, then it was partly able to internally adjust their outputs to account for their default relative scaling differences. Since one should not rely on this in general, we show the results of using the preconditioned LMS absorption curves in this article.

Switching our attention to the vector field itself, since the original convergence papers spoke specifically of “convergence,” which is the inverse of divergence (Schey, 2004), we sought additional algorithms that focused on the local contributions to the global tendency of a vector field to converge on a single point. We did not use divergence explicitly, since it is highly sensitive to small local fluctuations, and vector fields with such small local fluctuations will appear as if they do not converge on a point when analyzed only with divergence, even if they actually do converge. In particular, divergence is best applied to (mostly) smoothly varying and uniformly sampled vector fields. In the case of our stimuli, the color space is not uniformly sampled, as seen in our vector field in Panels C and D of Figure 5, and that makes “streamline” plots a better option. “Streamline” plots are typically used for analyzing fluid flow, and they show the trajectory a particle would take if it were dropped into this flow at different points. In the case of color changes induced by glass objects, “streamline” plots show the overall tendency of colors to be “pushed” towards a convergence point when filtered by a transparent object. We calculated the corresponding “streamline” plot using the “streamslice” function of MATLAB. To be clear, the “streamline” plot is not how the convergence model was implemented or tested in the original work. However, it is a more general operation that captures the same properties as the original convergence operation, but in a more localized manner. We applied it here to see if it would provide any further insight.

To do the “streamline” analysis, we started with the vector field in Panel C of Figure 5. However, this vector field cannot be used in its original form in a streamline analysis. Since the walls of the room in our stimulus have broad regions that are all the same color, it was found that many pixels from Panel A map to the same point in color space. However, because the curved transparent object has a different optical thickness for filtering at each point in the image (due to changes in orientation of the walls, but not due to any changes in thickness) and because specular reflections, caustics, and shadows are also in the filtered region, many of these pixels that initially map to the same point eventually spread out in color space after filtering and map to many different points. Panel D of Figure 5 shows a zoomed region of Panel C to highlight this. However, this pattern is not conducive to “streamline” calculations, since it is a one-to-many mapping, resulting in a vector field that is not continuously differentiable in the format that is expected by many vector field/vector calculus formulae.

To account for this, we created a basic interpolation approach that went through the bounding box around the cloud of vectors, at 100 equally spaced points along each axis of the bounding box (so, 1,000,000 equally spaced points spanning the interior of the bounding box). At each point, any vectors within a small box (the “averaging box”) centered at the point of interest (the side length of the box was equal to the step size of the interpolation algorithm) were averaged, and that average vector was saved into a separate array at the same point as the center of the “averaging box.” To be specific, when we averaged a vector, we averaged first one component of the vectors (e.g., the x-coordinate) and then separately averaged the other components of the vectors. The resulting coordinates were used to construct the final averaged vector that was placed at the center of the “averaging box.” If no vectors were within the box, then a vector of length zero was saved at the center of the “averaging box” in the separate array. The result was a vector field that was very similar to the original, but with each point having only one vector associated with it. The result of this interpolation procedure is shown in Panel D of Figure 5. The streamslice() function of MATLAB was then applied to the resulting vector field, by first projecting the vector field separately into the “red-green”/“blue-yellow” plane and then the “blue-yellow”/“light-dark” plane of the MB-DKL space and applying the streamslice function on each of these projections. This allows for a clearer two-dimensional (2-D) visualization of the overall trend of the 3-D vector field.

#### Image statistics

We assessed how well different image statistics could predict the matches of observers. These statistics were computed on the CIELAB and LMS color distributions of the pixels filtered by the glass Glaven. The statistics were computed with a program written in the Go programming language (v1.13.3) (The Go Programming Language, 2019) that used the color space conversion routines mentioned in the previous section.

Essentially, the Glaven was segmented from the image, and the analysis was run on this segmented region. This included the highlight and some of the caustics and shadows. For the uniform patch-matching element, we initially considered the following statistic:

• Average along the three axes of the CIELAB space (i.e., the average CIELAB color) for the filtered region of the image

Other statistics for the uniform patch were considered later and are described in the context of the “Results,” where the reason for testing them becomes clearer. For the flat filter matching element, we considered an expanded set of statistics:

• Average along the three axes of the CIELAB space (i.e., the average CIELAB color) for the filtered region of the image
• The average color of the 5% brightest pixels for the flat filter, when represented in CIELAB space (a.k.a., “White Point”), and those of the glass Glaven, excluding the specular highlight
• Ratio of the mean cone excitations for the filtered and unfiltered regions of the image, as suggested by Khang and Zaidi (2002a)
• Ratio of the standard deviations of cone excitations for the filtered and unfiltered regions of the image, as suggested by Faul and Ekroll (2011, 2012), Faul and Falkenberg (2015), and Faul (2017)
• The robust ratio model, as suggested by Faul and Ekroll (2011)

Please note that the LAB statistics were indeed computed on the full LAB color distribution. For example, the average along the three axes of the CIELAB space was computed by first converting all corresponding linearized RGB values to LAB and then computing the average color. Doing the analysis this way might seem incorrect: Since the LAB space is a nonlinear transformation of the linearized RGB values, then computing the average in the LAB space will not correspond to the colormetric average obtained by first computing the mean in a linearized RGB space and then converting that mean RGB value to LAB. However, the “correct” choice depends on the task and the research question that one is trying to answer. If one is working with color tolerances and doing quality control for industrial purposes or if one is analyzing data from color-matching experiments in a Maxwellian view system, then the colorimetric average is usually preferred. However, if one is looking to model higher-level percepts, like the color of curved transparent objects, then the measures should reflect the perceptual mechanisms that one is trying to understand. For example, the CIELAB space intends to account for many different factors that can influence the perceptual uniformity of color space, more than the LMS or MB-DKL spaces account for. This means that points in CIELAB can roughly represent the inputs to visual cortex and higher-level mechanisms. One could reasonably assume, before conducting any experiments, that the visual system might only start to represent the color of a transparent object at a stage that comes after the earlier stages. In that case, there is a chance that the visual system actually waits until the “LAB chromatic distribution for the Glaven” is available and then it computes the average color of this LAB distribution. If one starts from this assumption, then it is perfectly fine to compute the average in the LAB space, instead of first computing the mean in the linearized RGB space and then converting that mean to LAB. However, to be sure that results could not be strikingly different with either approach, we computed the CIEDE2000 color difference between the average colors of our glass Glavens from Figure 2, computed either directly in LAB or first computed in linearized RGB and then converting that mean linearized RGB value to LAB. The average difference between the two approaches was 3.68 ± 1.82 JNDs, which is negligible in comparison to the noise of observer responses.

The ratio of mean cone excitations is computed by segmenting the image into filtered and unfiltered regions. In the case of the Glaven test stimuli, the filtered region is the region that is covered by the glass Glaven itself, and the unfiltered region is everything else. Similarly, for the flat filter matching stimulus, the filtered region is that region covered by the simulated filter, and the unfiltered region is the achromatic Voronoi background surrounding it. Then, for each of these regions, the mean excitations of the L, M, and S cones are computed. Last, the ratios of these means (again, computed separately for each cone class) are calculated, with the filtered region typically in the numerator and the unfiltered region in the denominator. This results in a vector with three elements: [ratio L, ratio M, ratio S].

The robust ratio model of cone excitations uses additional equations that are directly derived from a reduced version of the underlying image generation process to bolster the estimate of a 3-D color code for the flat filter, which will be denoted as τ. In this article, we test the most robust and general version proposed in Faul and Ekroll (2011) (their “case 3” in their “Robust parameter estimation” section). This robust version first finds the cone class with the largest standard deviation of responses from the unfiltered, background colors (denoted as cone class MSD). It then computes the following values for that cone class:

$$\begin{array}{ccc} \tau_{MSD} & \;= & \frac{std(P_{MSD})}{std(A_{MSD})}\\ u & \;= & mean\left(P_{MSD}\right)-\tau_{MSD}*mean\left(A_{MSD}\right)\\ v & \;= & \left(\tau_{MSD}+u\right)*I_{MSD}\\ \delta & \;= & \frac{u}{v} \end{array}$$

where P denotes all colors from the filtered region, A denotes all colors from the unfiltered background, I denotes the color of the illuminant, and τ is the to-be-determined 3-D color code for the flat filter. In their model, δ is also related to the direct reflection factor, k, of the underlying physical model that governs the optical properties of a flat filter. In this most general version, the method for determining I, the illuminant color, from the image is left unspecified, so in this article, we have taken the average of the unfiltered background colors in a scene as an estimate of the illuminant color, since this seems to be what observers do (Ennis & Doerschner, 2019) and it is one possibility suggested in other parts of Faul and Ekroll (2011).

Once the values of the terms above are known, then one uses them in the following formula to come to a final estimate of the three-component color code, τ:

$$\tau_{i}=\frac{mean\left(P_{i}\right)-u*\delta *I_{i}}{mean\left(A_{i}\right)+\delta *I_{i}}$$

for i=L, M, and S cone classes. If one investigates this final equation for the robust ratio model, they will see that it is a modified version of the RMC, with offsets applied to the numerator and denominator that attempt to correct for any biasing due to the illuminant (I) and the specular reflections (δ, which is related to the direct reflection factor, k). Because of that, if we have a scene where the illuminant color and the background are fixed and we only vary the specular transmission component of a glass object (specular transmission and specular reflection are allowed to independently vary in physically based rendering systems, such as Mitsuba, so we can also fix the specular reflection component), then the RMC and robust ratio model will highly correlate. Faul and Ekroll (2011) also show a simpler version of this more general model, where one indirectly computes τ via a scaled version of the RMC. We do not consider the simpler version in this article, since our tests showed it to be less robust than the more general version. Faul and Ekroll (2011) also propose that τ can be computed as the ratio of the standard deviations of cone excitations (RSD). This RSD model is the primary model used in other reports (Faul & Ekroll, 2012; Faul & Falkenberg, 2015; Faul, 2017) and is computed in the same way as the RMC, but with the standard deviations of cone excitations instead of the mean cone excitations.

The “White Point” statistic mentioned above is essentially the “brightest point on the object is most informative about albedo/surface color” rule (Giesel & Gegenfurtner, 2010; Toscani et al., 2013a; Gilchrist et al., 1999). We included this statistic in our analysis because it predicts the settings that observers make when matching the color of curved Lambertian objects. The brain could simply reuse this strategy when assigning a color to a transparent object. There is nothing “right” or “wrong” about whether or not the brain uses the “White Point”; it is merely a question of what the brain does in a given situation.

Note that these statistics are computed directly on the colors in the image. When computed in this fashion, there is no preprocessing stage that performs any color constancy operations.

#### Analysis of observer data

Since observer data were collected in MATLAB and saved in its MAT file format, a program written in Octave (v5.1.0) (Eaton et al., 2019) was used afterward to cycle through each observer's data and convert them to a CSV format that could be easily processed in the R programming environment (v3.6.1) (R: The R Project for Statistical Computing, 2019). For data that came from the experiments that used the flat filter matching element, the Octave program also used the saved data to re-create the flat filter that observers set for their match by passing the saved parameters for their match to a program written in the Go programming language (v1.13.3) (The Go Programming Language, 2019). This Go program would then compute and return the statistics mentioned above for the flat filter, using the same routines mentioned in the previous sub-section, “Image statistics.” After these processing stages, R programs were written to analyze data and make plots. Only base R packages were used for analysis and plotting. In particular, the key statistic computed for data that came from observers was the grand mean: that is, the mean of the observers’ mean settings, and so error bars in plots always show the standard error of the mean (*SEM*). In plots that show best-fit lines for data, these lines were always fit to the distribution of observer means, not to the grand mean data. In other words, they were fit to the data that were used to compute the grand mean. This was done to better capture the variance in the data via the fit. In the end, the difference in doing fits either way was negligible.

In all of our experiments, it was possible for observer settings to go out of the monitor gamut (i.e., they could make matches that had R, G, or B values that were less than 0 or greater than 1). When this happens, the monitor will either clamp the RGB values to the range of [0,1] or it presents a random color. To prevent any contamination of results, we excluded any settings that went out of gamut. This was rather rare. In the worst case, for the experiment where observers viewed the Glaven against the multicolored wall and used the uniform patch to make a dye match, 6.25% of trials had to be excluded. Overall, the average percentage of trials that had to be excluded from an experiment was 3.11% ± 2.39% of all trials across all observers for the given experiment. This was deemed acceptable, and despite rejecting a few trials, we were led to parsimonious conclusions across all experiments and consistent results across all observers.

#### CIEDE2000 maps

It is already known from work by Giesel and Gegenfurtner (2010) and (Toscani et al., 2013a, 2013b) that when making color matches to Lambertian objects, observers use the most luminant region of the object to guide their match (i.e., the “White Point”). To gain some perspective on which regions of the object observers might use when making a match to a transparent object, we created images of the glass Glaven where we only colored in those pixels that were 15 CIEDE2000 units or less from the grand mean of the matches that observers made. When paired with the CIEDE2000 color difference metric (Sharma et al., 2004), CIELAB comes closer to being a perceptually uniform space, although still not fully uniform (Ennis & Zaidi, 2019), but CIEDE2000 is still the recommended metric for computing JNDs in the CIELAB space, so it is what we use here. The maps were computed with a Go program (v1.13.3) (The Go Programming Language, 2019) that transformed each test image into the CIELAB space and used a mask to test only those pixels that were filtered by the Glaven. We only computed CIEDE2000 maps for the data coming from the uniform patch experiment with the “proximal match” instructions.

For each scene that was processed, a new companion image was made that was initially all black. For any pixel in the masked region that passed the CEDE2000 threshold, its original RGB value was placed in the corresponding pixel of the companion black image. All other pixels in the masked region were converted to grayscale using the following formula:

GrayValue=0.299*R+0.587*G+0.114*B,

which is the formula used by Go's color package and follows the RGB to YCbCr color space conversion given in the JFIF specification. This grayscale value was then divided by 1.5, so that the bright intensity of specular highlights would not make them look accidentally colored. The slightly darker gray values also help emphasize the actually colored portions of the map. These resulting grayscale values were then placed in the corresponding pixels in the companion black image. The CIEDE2000 maps are similar to analysis done in Giesel and Gegenfurtner (2010), and they give a rough estimate of what might drive the observers’ percepts. It acts as a quick and coarse analog of an eye-tracking experiment, but note that it is by no means a replacement for an eye-tracking experiment or a form of eye-tracking.

## Results

### Applicability of the convergence model to curved transparent objects

**Figure 6.**
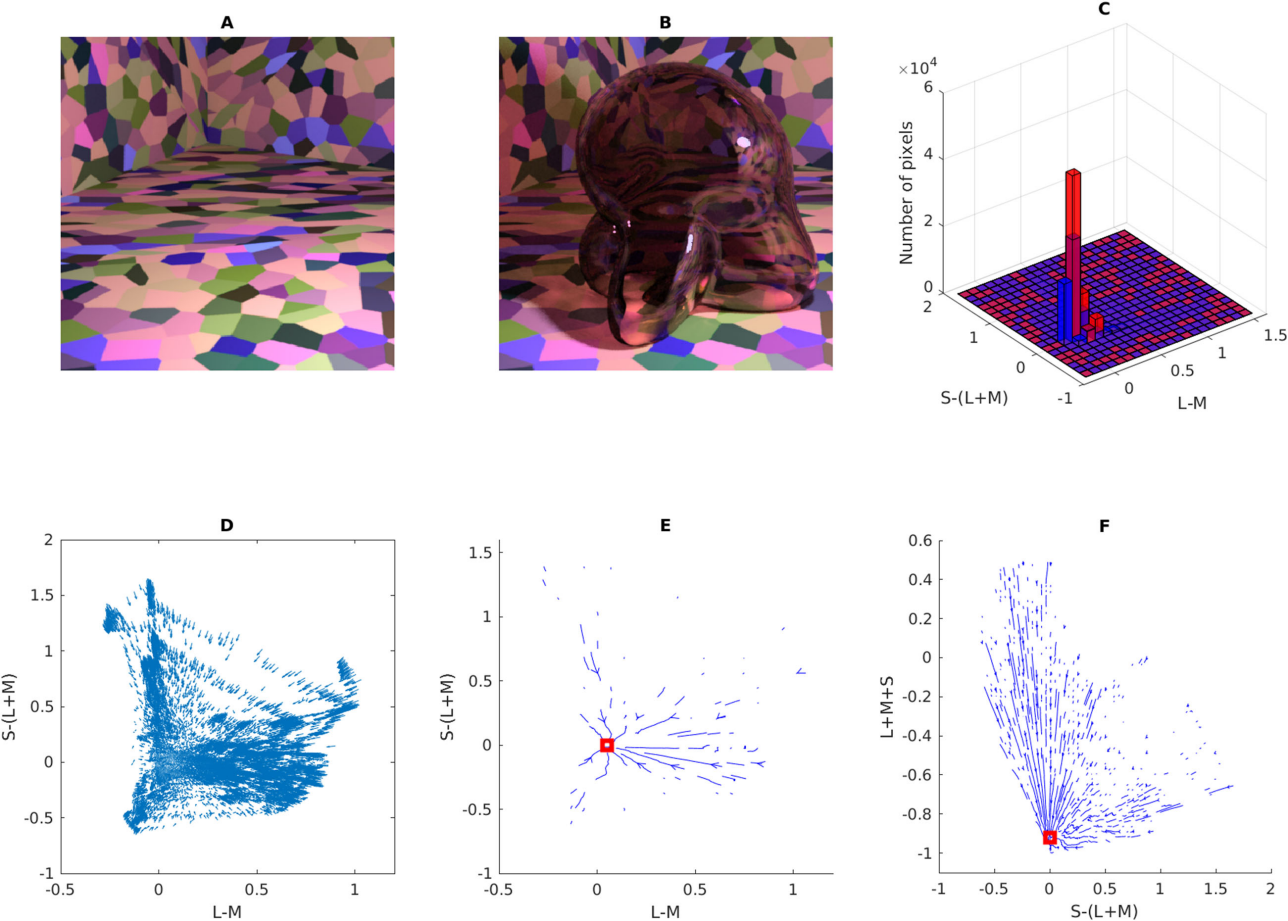
Results of applying two forms of the convergence model to the stimuli used in this article. (A) One of the main scenes tested in this article, but with the glass Glaven removed. (B) One of the actual stimuli used in the article: a colored, curved, glass object (a Glaven), placed in the room from Panel A. The convergence model focuses on analyzing the change in color induced by the glass Glaven in the region that it filters. (C) The isoluminant plane of the MB-DKL color space, where the colors of the pixels that are directly filtered by the glass Glaven are plotted as a blue transparent histogram. The red transparent histogram shows the result of applying the best-fitting 12-parameter Affine transformation to the colors of the unfiltered pixels in Panel A. (D) The vector field that connects the unfiltered colors from the image in Panel A (vector tails) with their filtered counterparts from the image in Panel B (vector tips). In this case, we show the interpolated field (see Figure 5 for the uninterpolated version and the “Convergence model analysis” section of the “Method” for a description of the interpolation process). (E) A “streamline” plot for the red-green (L-M) and blue-yellow (S-(L+M)) chromaticity plane of the MB-DKL color space. This streamline plot is computed from the vector field shown in Panel D. We see that there is actually an overall tendency for the color distribution to converge on a “reddish” color (marked with a red square that was placed “by hand”). (F) A “streamline” plot for the light-dark (L+M+S) and blue-yellow (S-(L+M)) plane of the MB-DKL color space. We see again an overall tendency to converge on a point (marked again with a red square that was placed “by hand”), with many colors becoming darker and a few becoming a bit brighter. The darkening effect is due to the absorbing/reflecting qualities of the glass and the brightening effect is due to specular reflections/highlights and caustics.

In Panel C of Figure 6, we provide a test of the 12-parameter Affine map formulation of the convergence model for the pair of example images in Panels A and B of that same figure. In Panel C, we plot the actual filtered colors coming from the image in Panel B as a blue transparent histogram in the isoluminant plane of the MB-DKL space. The red transparent histogram shows the result of applying the best fitting 12-parameter Affine transformation to the original distribution of colors from the wall (Panel A) that are eventually filtered by the glass Glaven. Ideally, the red histogram and the blue histogram should line up on top of each other, but we see that while the red histogram comes close, there are noticeable discrepancies. In addition, even though parts of the histograms align, the histograms do not show if this best-fitting model is mapping the right pixels to the correct colors; they only show if the overall distributions overlap. For instance, a pixel in the wall might map to slightly purple after introduction of the Glaven, but at best, the histograms can only show that some of the pixels in the wall are mapped to that purple but not necessarily the specific pixel that we need to map to purple. Stated differently, we could make another image where we spatially scramble all of the pixels in the Glaven and plot its histogram and that will align perfectly with the blue histogram, even though the new Glaven will look like random chromatic noise. To be more exact, we instead need to investigate the RMSE, which should be low relative to applying an identity transform (i.e., no transformation). We have followed the process of D’Zmura et al. (2000), where we calculate the relative reduction in prediction error (RRPE) for the convergence model as $\left(RMSE_{identity}-RMSE_{convergence}\right)/RMSE_{identity}$. We do not convert colors to a threshold-scaled MB-DKL space for model comparison, though, since we do not apply the convergence model to observer data; we only apply it to images to see how well it accounts for the physical effects of curved, transparent objects.

**Figure 7.**
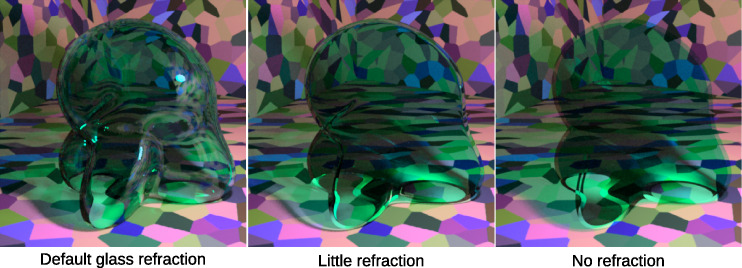
Examples of the three different refraction levels that we used in our tests of the convergence model. The “Method” section contains more detail about how and why these three levels were chosen, but the main intention was to estimate the influence of the optical distortions of refraction in the context of the convergence model.

We find that for our default refraction stimuli (examples of tested refraction levels shown in Figure 7), the MB-DKL version of the 12-parameter Affine convergence model shows a 57.3 ± 17.2% reduction in prediction error on average across all conditions that we tested, which is a drop from its ability to account for ∼96% of the variability in observer responses with flat filters in the original investigations. This does not mean that the central idea of the convergence model is no longer applicable, though. First, it might be that a modified version of the convergence model is capable of handling the extra complexity that comes with curved transparent objects. We deal with this possibility below. Second, it might be that the roughly 60% of variance that the convergence model accounts for is all that observers care about and is also all that they monitor. The extra complexity that the convergence model does not account for could be something that observers simply ignore and they are just interested in the magnitude and point of convergence. In other words, the visual system might use the same mechanisms for flat filters and for curved glass, and that is “good enough.”

In Table 3, we show the average RRPE for the best-fitting 12-parameter Affine maps. In this table, we average over fits for the full 3-D distributions, including luminance/lightness, and for 2-D isoluminant projections, as well as mask type, illuminant/body color, and whether or not dark pixels were excluded, since we found little difference in RRPE for these factors, as can be seen in the relatively small overall standard deviations. Otherwise, the average RRPEs were grouped according to color space and refraction level. Examples of the different refraction levels are shown in Figure 7 (please see the “Method” section for more details about how these refraction levels were made and chosen). Note that we also performed the analysis described above in the LMS and CIELAB color space. The LMS space was tested, because the original convergence model papers investigated that space, too. The CIELAB color space was tested, since there was the possibility that if the convergence model fails in the LMS and MB-DKL spaces for richer stimuli, then it might still hold at a higher-level representation. Details about these two spaces are contained in the “Method” section.

**Table 3.** Average relative reduction in prediction error (mean ± *SD*) for fits of the general 12-parameter Affine map from the original convergence model papers to our stimuli, broken down according to color space and amount of simulated refraction. Values are averaged over mask type, illuminant color, transparent body color, whether the fit was done for the full-color distribution or chromaticity only, and whether or not dark pixels were excluded. The relatively low standard deviation in all cases indicates that variations in all of these factors play relatively little role in the applicability of the convergence model to our stimuli. See the main text for more details.

	LMS	MB-DKL	CIELAB
No refraction	35.8 ± 8.74%	42.6 ± 18.8%	51.1 ± 13.0%
Little refraction	42.2 ± 7.76%	44.6 ± 18.5%	46.9 ± 15.1%
Default glass refraction	56.9 ± 14.8%	57.3 ± 17.2%	49.0 ± 13.7%

Considering the above, the 12-parameter Affine transformation-based convergence model in its original form is not fully generalizable to curved transparent objects that exhibit specular reflections, shadows, and caustics, but it is not performing poorly. One would then also wonder how well the 4-parameter version of the convergence model works in comparison. The 4-parameter version was the one accepted by D’Zmura et al. (2000) because in their investigations, it did just as well as the 12-parameter version and was simpler (if the simpler model does just as well, then it should always be preferred, unless one has additional evidence and a good reason that justifies rejecting it). We have tested the 4-parameter version with our data in the exact same way as we did with the 12-parameter Affine transformation version. We find that its explanatory power is basically on par with the 12-parameter version. For example, for the default refraction case, we get the following RRPE values in the three different color spaces: LMS = 58.6 ± 15.2%, MB-DKL = 59.2 ± 18.0%, CIELAB = 46.9 ± 15.9%. Just like before, we found no appreciable differences for inclusion/exclusion of dark pixels, different refraction levels, or different masks. Taken all together, this indicates there is a significant amount of convergence happening, since the 4-parameter model is able to account for about 60% of variance on average for our stimuli.

Before we continue, we would like to take a brief detour to discuss the functionally similar “over” operator from Porter and Duff (1984). The “over” operator models the optical effects of surfaces with partial coverage, such as a discontinuous thin fabric with holes and gaps between the threads (McGuire, 2019) (cf. the diagrams in Porter & Duff, 1984). It is a form of alpha blending (Foley et al., 1990), but it does not model a continuous surface that transmits light, such as glass. For example, a red glass will cast a shadow with red caustics, and it will also appear black (or at least much darker) before a green surface, because the red glass and green surface together absorb all (or most) of the incoming light when overlaid, dependent on the exact absorption and reflectance distributions of the red glass and green surface. On the other hand, a red fabric with gaps between the threads will cast a black shadow with no caustics, and it will still appear red before a green surface (McGuire, 2019). It is just in the situation of 2-D flat surfaces that these two cases can produce similar results, and in that case, the “over” operator can be used as an efficient operation in computer graphics to approximate the appearance of thin flat filters, but the “over” operator alone cannot account for what happens with transparent objects in general. Consider, for example, the following comparison of their equations. First, the convergence model has more freedom than the “over” operator. In the case of the 4-parameter convergence model, the scaling of the unfiltered color and the translation that it undergoes are not coupled in any way (i.e., a general linear combination), whereas the “over” operator is strict linear interpolation with the restriction that the coefficients add up to 1 (i.e., a convex linear combination), so that if the contribution of the convergence point increases, then that of the unfiltered color must necessarily decrease. Essentially, the 4-parameter convergence model is

(1) $$C_{filt}=aC_{orig}+t,$$

where a is a real scalar and t is three-component translation vector, both of which can independently vary, while $C_{orig}$ and $C_{filt}$ are the position vectors for the original, unfiltered color and the resulting, filtered color, respectively. (In this model, different choices of a and t correspond to different flat filters.) On the other hand, the “over” operator model is

(2) $$C_{filt}=aC_{orig}+\left(1-a\right)C_{conv},$$

where a is a real scalar and $C_{orig}$ and $C_{filt}$ are defined as before, but now the amount of translation (1-a) is dependent on the amount of scaling (a), with the additional restriction that their sum is 1. (In this model, a and $C_{conv}$ correspond to surfaces of different partial coverage.) This is a different model from the 4-parameter convergence model, since t in Equation 1 does not need to equal $\left(1-a\right)C_{conv}$ in Equation 2, although is free to do that. Essentially, the “over” operator is a special case of the 4-parameter convergence model, but a special case that curiously corresponds to a different class of transmissive materials.

Furthermore, the reflectance of the just mentioned red fabric can be constructed to have the same luminance as the green surface that it covers, which is very similar to the “physically impossible” flat filters of D’Zmura et al. (2000) that alter chromaticity but are isoluminant with the background. In fact, a subset of these “physically impossible” flat filters can be produced by an application of the “over” operator: just do a convex linear interpolation between isoluminant colors. So, it seems more likely that at least some of the “physically impossible” flat filters are actually physically possible opaque discontinuous surfaces with fine gaps in their surface, viewed from a distance where the gaps are not resolvable, and that these stimuli are a special case that are either stimulating the same mechanisms as transparent objects (i.e., the brain is just being economical and reusing a strategy for perceiving both kinds of materials) or stimulating separate, but similar, mechanisms related to the perception of partial coverage.

Returning to our analysis, notice that the results for the CIELAB space are consistently worse than for the LMS or MB-DKL color spaces. This indicates that the pattern in the visual input that supports the Affine convergence model begins to degrade as signals progress through the visual system. The amount of degradation we find for the CIELAB space could be the limit to how well the Affine convergence model accounts for transparent objects, but if the brain estimates the point and magnitude of convergence, then this at least indicates one of two things: Either the point and magnitude of convergence are extracted at the level of the cones (LMS space) or the LGN (MB-DKL space) and that is preserved separately on the way to higher-level stages of visual processing where it is used to determine the color of a transparent object, or the more likely possibility that convergence generally emerges in a different way at later stages and is then extracted by different mechanisms, with flat filters being a special exception where convergence can be measured in the early visual input.

Indeed, our “streamline” form of the convergence model (Panels E and F of Figure 6) shows that the introduction of a transparent object into a scene does cause many of the filtered colors to converge to a point, just with additional non-linearities that an Affine model cannot capture (the nonlinearities are introduced by caustics, shadows, interreflections, specular reflections, and varying optical filtering due to changes in the curvature of the walls of the Glaven). The natural question then is “since convergence holds in a modified form for curved transparent objects, then how does the visual system evaluate/measure it?” The original convergence investigations suggested that the visual system has a way to connect an unfiltered portion of a surface with its filtered counterpart at the edges of the glass object (D’Zmura et al., 1997). These samples at the edges of the object would then be enough to recover the convergence transform. However, for a curved object, it is at these edges where refraction will usually have some of its strongest effects, making it difficult to use the originally proposed edge-traversing algorithm to match a filtered color with its unfiltered counterpart. Inverse optics methods are not possible, as it is highly unlikely that the visual system is capable of tracing light paths to undo the resulting optical distortions. In addition, it can be seen that specular reflections are often present at the curved edges, and caustics/shadows are around the edges at the bottom of the glass, further masking or distorting the absorbing properties of the glass.

**Figure 8.**
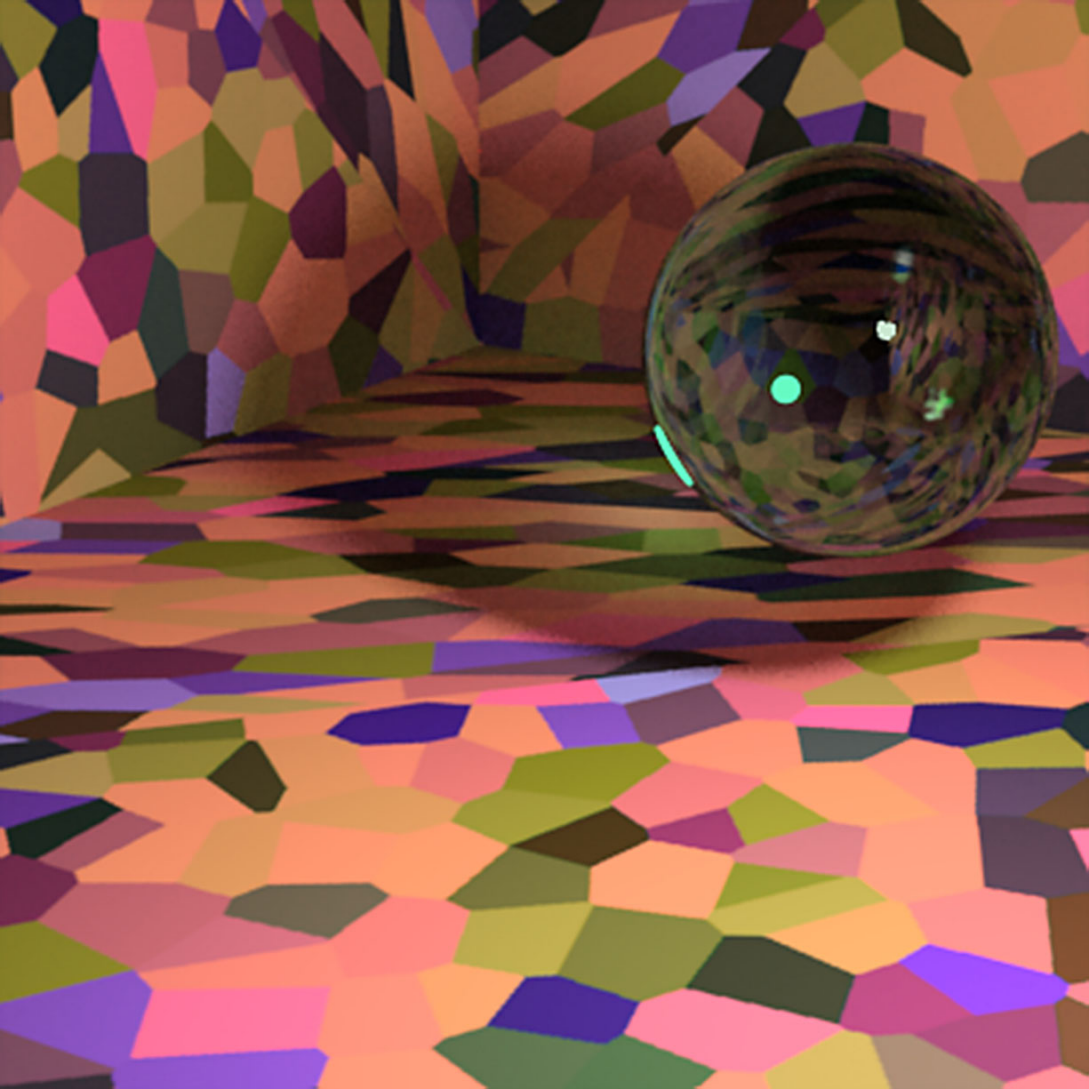
A scene where the Gestalt principles of continuity, connection, and good figure cannot be applied. The floating transparent sphere exhibits considerable refraction across its whole boundary, meaning that only the Gestalt principles of proximity and similarity could potentially be applied to extract the magnitude and point of convergence. See the main text for more details.

There are, however, some regions at the “foot” of the Glaven, where there is continuation, connection, good figure, and proximity between the filtered and unfiltered parts of patches in the floor. This suggests that the brain could use Gestalt principles to find out how the glass transforms the colors of objects, but continuity, connection, and good figure do not apply in general (because other shapes can be used where refraction at the edges breaks them; see Figure 8). This means that the brain would probably want to find pairs of filtered and unfiltered patches that simultaneously maximize the Gestalt principles of proximity and similarity. Proximity is important, because one does not want to risk a mistake by comparing a filtered color to an object of similar color that is several meters away: Such a remote object would be irrelevant because it is unlikely that any part of it is filtered by the glass. Similarity is important because of the way convergence works: If all of the colors are converging toward a point, then a filtered color will be the most similar to its unfiltered counterpart, provided that the pattern of convergence is not highly nonlinear (our tests so far indicate that the pattern of convergence probably will not be highly nonlinear in normal viewing situations, but we have not rigorously confirmed this). However, it is unclear to us if the brain uses Gestalt principles or if it computes statistical descriptions of the stimulus and determines the magnitude and point of convergence from the value of these statistical descriptors. Since there already are three established statistical descriptors that can predict the colors of flat transparent filters (the RMC, the RSD, and the robust ratio model that were discussed in the “Introduction” and “Method” sections), we have decided to first test and compare their applicability to curved transparent objects. The potential role of Gestalt principles in determining the color of glass is an interesting path of research for future investigators.

Although our investigation here does not establish the exact method by which the brain samples the scene to compare the unfiltered and filtered color distributions (this would require eye tracking and image manipulations at the least), our analysis of the convergence model at least shows that a physical regularity is shared across both flat filters and curved glass and the brain could utilize it for assigning colors to transparent objects, despite the additional complexity that is not captured by the original Affine convergence model. The investigations that we present below aim to answer two questions: (1) If a transparent object has a single, clear, and defined color and it can cause filtered colors to converge to a point, then is it possible for observers to make a sensible match with a single flat uniformly colored patch (i.e., a patch that looks more like paper and does not appear transparent at all [Wittgenstein, 1978])? and (2) the RMC, RSD, and robust ratio model statistics that were discussed in the “Introduction” and “Method” sections have already been established for flat filters and they could potentially be a way for the brain to encode the magnitude and point of convergence for glass objects in general, so do observers’ color matches with a flat filter indicate that these statistics are relevant for curved transparent objects?

### Matches made for the multicolored Voronoi background

**Figure 9.**
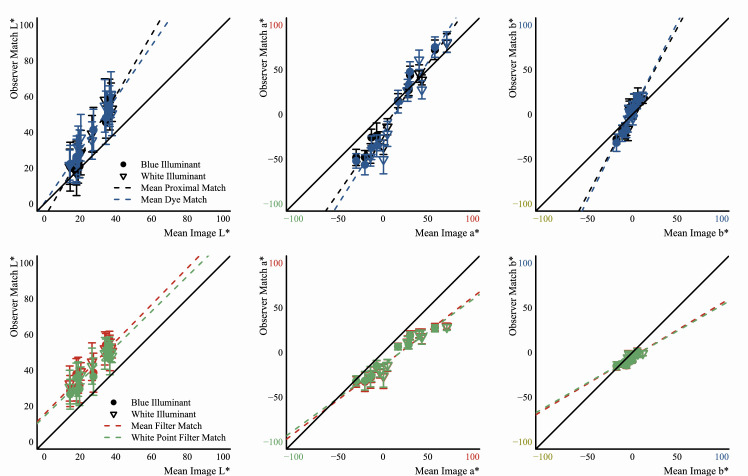
Results of matching experiments with the multi-colored Voronoi background. Each Panel shows the location of the resulting match on the three axes of the CIELAB color space: left Panels = L*, middle Panels = a*, and right Panels = b*. We have split the data across two rows to aid visibility. The endpoints of the axes in the middle and right Panels are colored to indicate the approximate perceived colors along the respective color dimension. The x-axis in each Panel represents the mean of the filtered region of the test image (i.e., the region occupied by the Glaven) for the respective color dimension. The y-axis represents the average match of observers for the same color dimension. Filled dots are matches made under the blue illuminant and empty triangles are matches made under the white illuminant. Error bars show the standard error of the mean and the color of the symbols denotes the experimental condition: black = proximal match (uniform patch), blue = dye match (uniform patch), red and green = filter match. The difference between the red and green points is whether we plot the mean of the filter match on the y-axis or the color of the brightest region (i.e., the “White Point”) of the filter match. In both cases, the mean and the “White Point” of the filter match are plotted against the mean of the image. The regression lines indicate the general trend of observer settings and can be roughly compared to the unity line to see whether or not observers’ settings track the mean color of the region filtered by the Glaven. In this and all following plots, these regression lines were always fit to the distribution of observer means, not to the grand mean data. Please see “Analysis of observer data” for more info.

The average matches of observers for the “proximal match” instructions with a uniform patch, the “dye match” instructions with a uniform patch, and the mean colors of the flat filter matches for the experiments with the multicolored Voronoi background are shown in Figure 9. We also show the color of the “White Point” statistic for the flat filter in Figure 9. We see that the lightness (L*) of observer matches is consistently higher than the mean lightness of the Glavens and that there are noticeable deviations between the mean colors of the settings and the mean colors of the glass Glaven. It should also be noted that there are indeed some differences between the flat filter and uniform patch settings. This is considered in more detail in the “Discussion” section.

**Figure 10.**
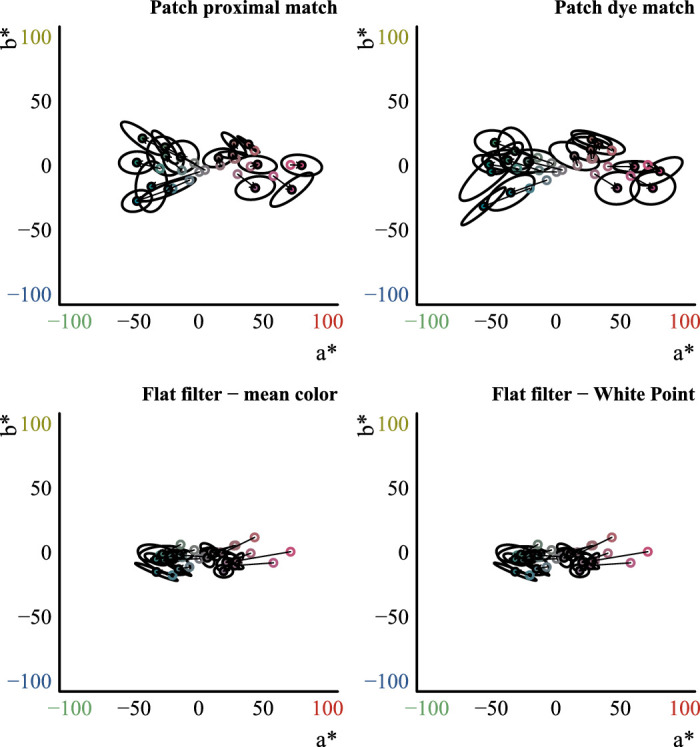
The same results as shown in Figure 9, but now projected into the chromaticity plane (a*, b*) of the CIELAB space. The mean colors of the Glavens are the empty circles with the colored borders, and the average settings of observers for the different experiments (see titles of Panels) are shown as black circles that are filled in with color. The colors of these symbols only roughly correspond to the color at that point in LAB space and are merely meant as visual aids. The arrows connect the mean color of a Glaven (tail) with the corresponding average observer setting (tip), and the ellipses around the average observer settings show one standard error of the mean (*SEM*), as an error ellipse derived from the covariance matrix of the corresponding data. Observer matches are usually several LAB units from the mean color of the Glaven, sometimes being 20 units away, which is much larger than a JND. The mean color and “White Point” are not what observers are matching.

In Figure 9, we have also plotted the color of the “White Point” statistic for the flat filter matching element against the mean color of the glass Glaven. There are two reasons for this. First, since the filter-matching element has spatial variation, there are a number of features that observers could attempt to match to the glass Glaven, and the mean does not necessarily have to be one of these features. Second, it is known from work with curved Lambertian objects (Giesel & Gegenfurtner, 2010; Toscani et al., 2013a, 2013b) and glossy objects (Granzier et al., 2014) that when observers are given a uniform patch and asked to set it to the color of the object, they often match it to the brightest region on the object, excluding highlights in the case of the glossy object. This implies that observers could follow a similar strategy for many different types of materials and that regardless of whether they use a uniform patch or a flat filter as the matching element, they will match “brightest” region to “brightest” region. If this were the case, then the green points should considerably differ from the red points in each Panel. However, the green points essentially overlap the red points in each Panel, so it is unlikely that the “brightest” region of a transparent object is what drives the associated color percept. We can look at the data another way by plotting the mean color of the glass Glavens and the mean color of observer matches in the chromaticity plane (a*, b*) of the CIELAB color space. We show this in Figure 10, where the data from Figure 9 are projected into the chromaticity plane. It is readily apparent that observers are not matching the mean color or the “White Point” of the transparent objects.

**Figure 11.**
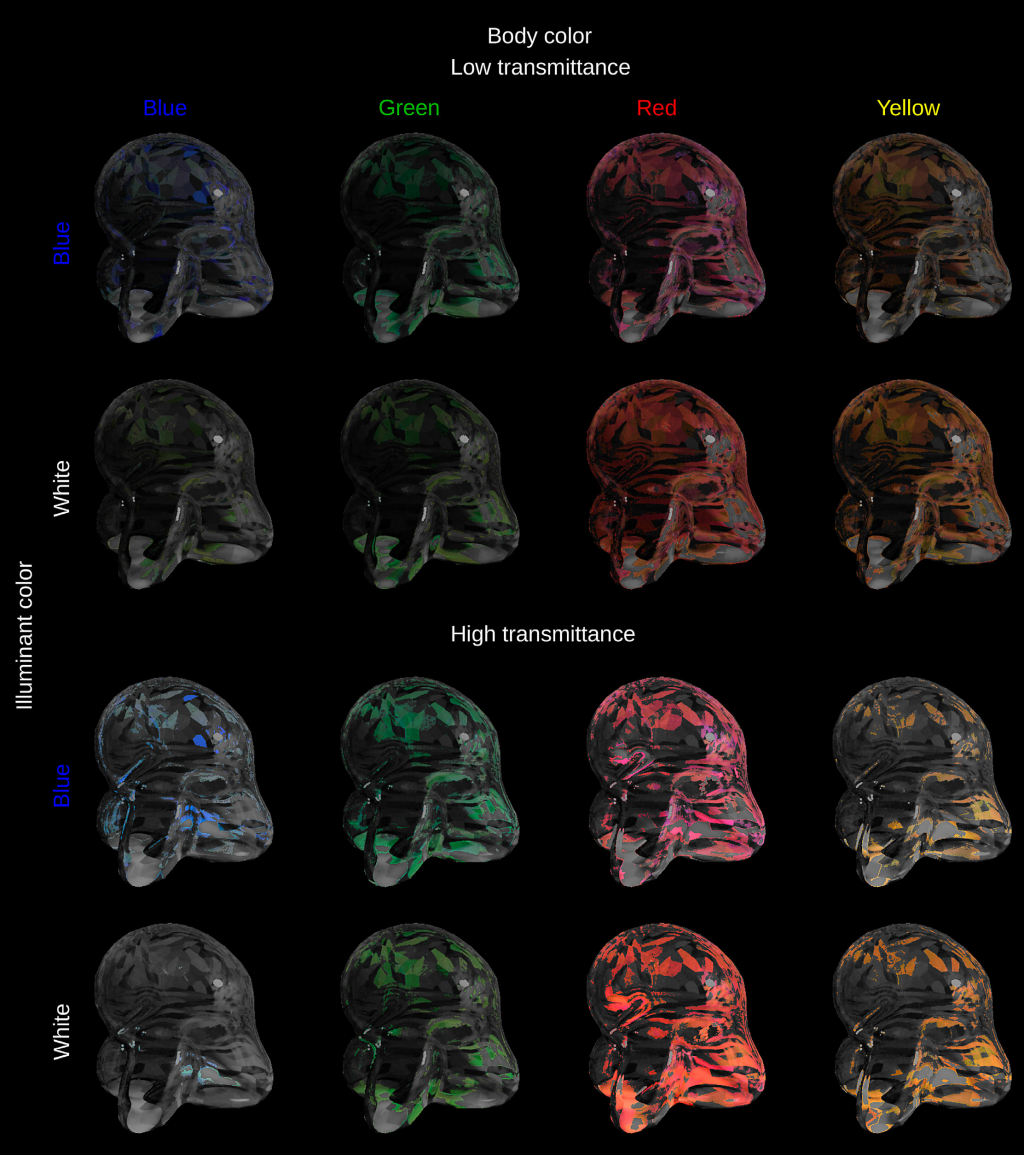
CIEDE2000 maps of the glass Glaven stimuli, where pixels are colored in with their original color if that color is located 15 CIEDE2000 units or less from the color of the “proximal match” with the uniform patch-matching element (the color in question being the grand mean of observer matches; see “Method” section for more details). The pattern is essentially the same for the “dye match” instructions. See main text for an interpretation of the results.

If we look at the CIEDE2000 maps in Figure 11 for the “proximal match” uniform patch instructions, we find that observer matches are close to colors on many different regions of the Glaven stimuli. There is no single specific region that stands out from others. With this analysis, it seems that observers are doing their best to squeeze all the variations in the Glaven stimuli into the single color that the uniform patch allows them to set, essentially being a sort of summary statistic. After examination of the CIEDE2000 maps, one might consider that the most saturated color (many pixels in the maps are rather saturated) or the most frequent color (many pixels are colored) is what observers are matching. We calculated the most saturated color in CIELAB space by calculating the Euclidean distance from mid-gray for the chromaticity coordinates of each pixel and dividing that distance by the lightness at the pixel (Schiller & Gegenfurtner, 2016). We excluded any pixels with a lightness less than 1 to exclude dark pixels and to prevent division by 0. The specular highlights were also excluded, as well as any pixels with a lightness greater than 85 units on the L* axis in order to exclude any excessively bright pixels that were missed by our specular highlights mask. We then computed the average color of the top 5% most saturated pixels, which was taken as the most saturated color. To compute the most frequent color (again in CIELAB space), we divided the bounding box around the cloud of colors corresponding to the pixels in the glass Glaven (excluding the specular highlights) into boxes of unit size. We stepped through the unit boxes and, at each one, counted the number of colors within it. The center of whichever box had the most colors was taken as the most frequent color (Feitosa-Santana et al., 2020).

**Figure 12.**
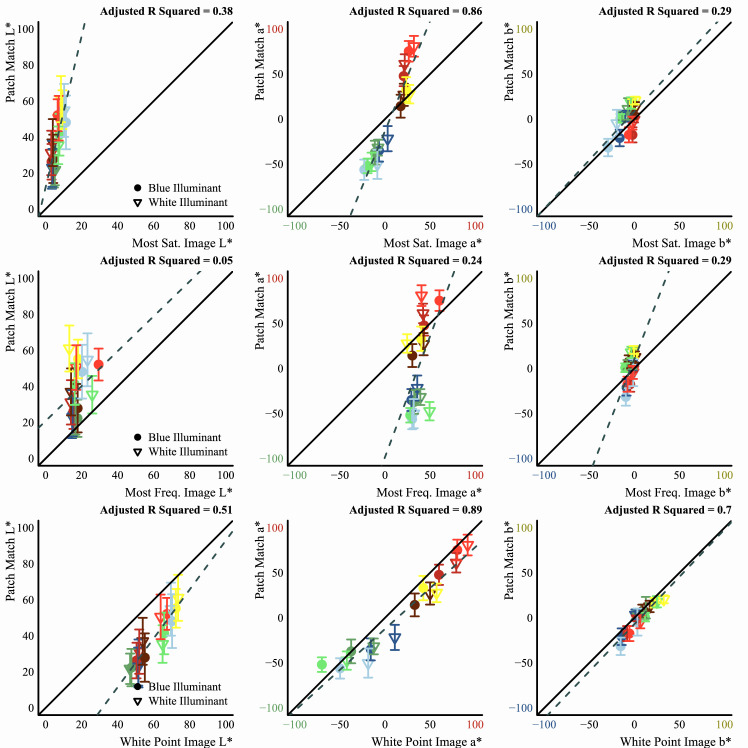
Comparison of the uniform patch match to the White Point (top row), most saturated (middle row), and most frequent (bottom row) colors in the Glavens, inspired by inspection of the CIEDE2000 maps; see main text for how they were computed. The colors of points correspond roughly to the body color of the Glaven, their lightness corresponds to the transmittance level of the Glaven (high = lighter; low = darker), and the plot symbol specifies the illuminant in the scene (see legend).

We have plotted the results of this analysis in Figure 12, where it can be seen that both of these statistics are clearly worse than the mean color. We have also included a comparison of the uniform patch setting to the White Point statistic for the Glavens, which captures observers’ chromaticity matches surprisingly well but predicts the wrong lightness for their settings.

**Figure 13.**
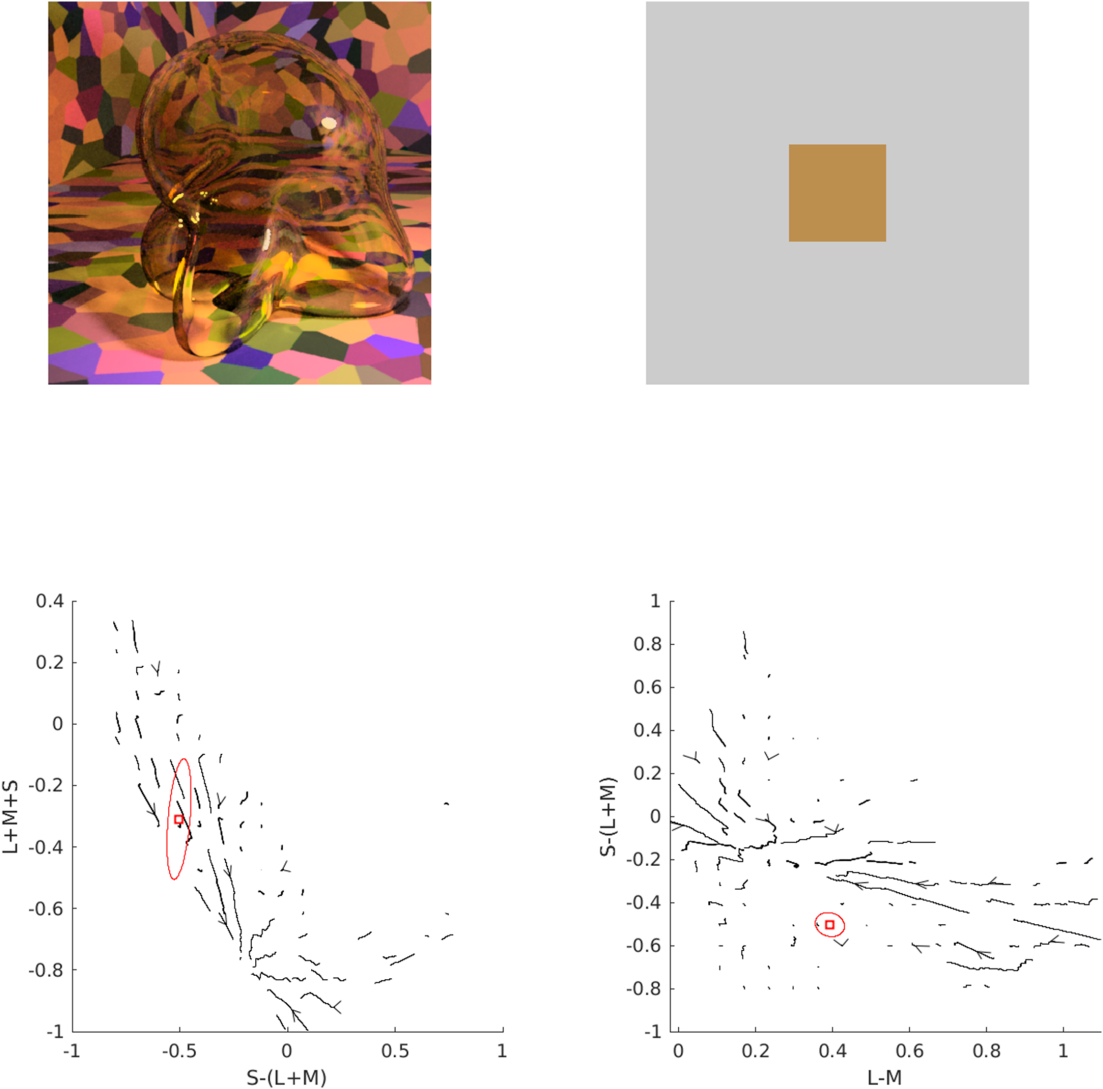
Comparison of the population average uniform patch match to the pattern of convergence for the high-transmission yellow Glaven under the white illuminant. Top left: The Glaven stimulus. Top right: The population average uniform patch match. Bottom left: Streamline plot in the “blue-yellow” (x-axis), “light-dark” (y-axis) plane of the MB-DKL color space. The population average match is shown in red and the covariance ellipse shows 1 *SEM*. Bottom right: A streamline plot, plotted in the same way as in the bottom left Panel, but for the “red-green” (x-axis), “blue-yellow” (y-axis) plane of the MB-DKL color space.

**Figure 14.**
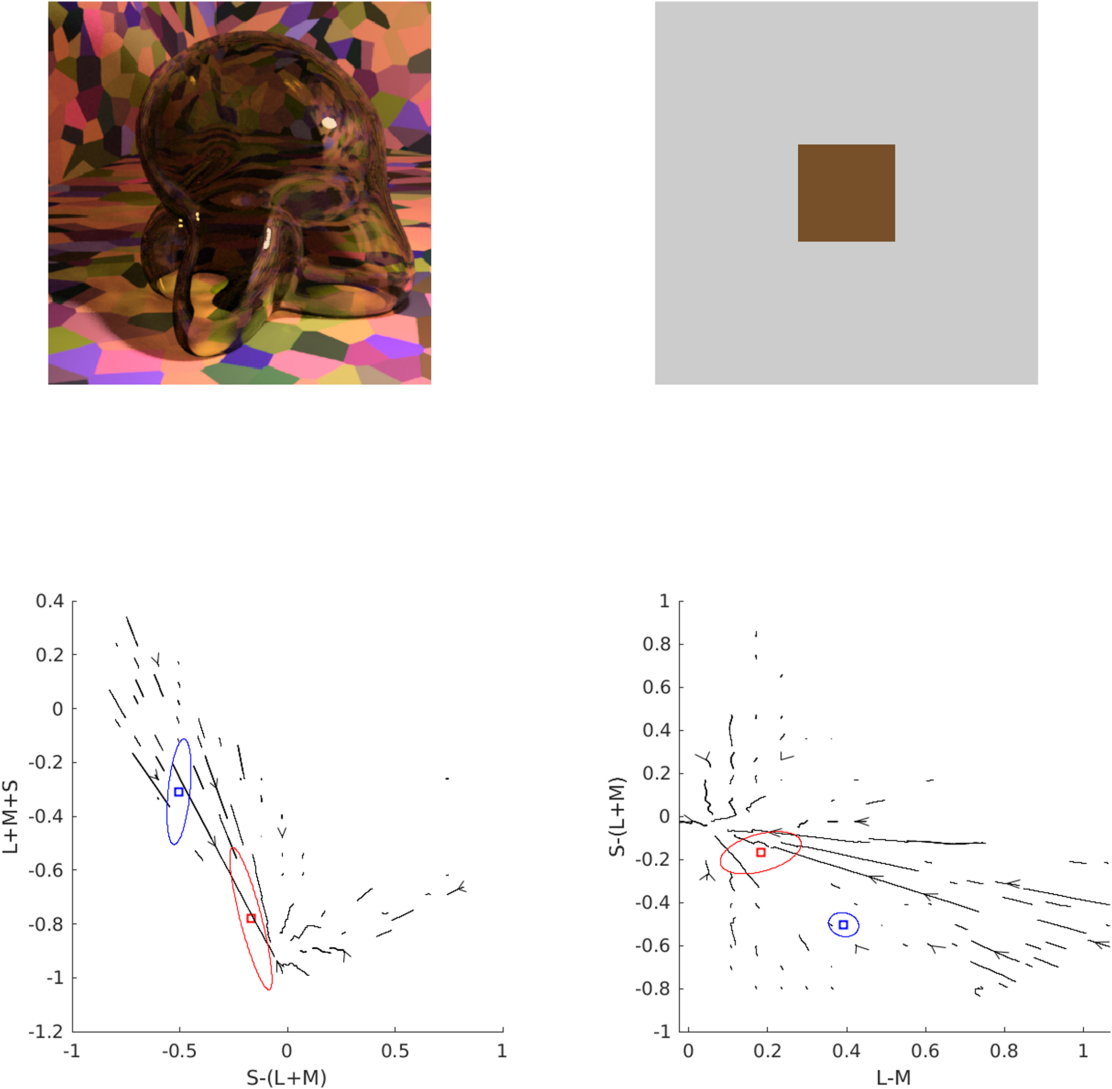
Comparison of the population average uniform patch match to the pattern of convergence for the low-transmission yellow Glaven under the white illuminant. Top left: The Glaven stimulus. Top right: The population average uniform patch match. Bottom left: Streamline plot in the “blue-yellow” (x-axis), “light-dark” (y-axis) plane of the MB-DKL color space. The population average match is shown in red and the covariance ellipse shows 1 *SEM*. The blue square and its blue covariance ellipse show the data from the previous figure (Figure 13) to see if observers always match to the final point of convergence. See the main text for more details. Bottom right: A streamline plot, plotted in the same way as in the bottom left Panel, but for the “red-green” (x-axis), “blue-yellow” (y-axis) plane of the MB-DKL color space.

One other possibility that we hinted at the end of the previous convergence model analysis section was that observers maybe make a match that is related to the convergence point associated with the glass object. In Figure 13, we compare the average uniform patch matches of our observers to the pattern of convergence for the high-transmission yellow Glaven under the white illuminant, where it is clear that observers are consistent in their matches, but they are not making a match to the convergence point, but rather some other aspect of the distribution. However, it could have been the case that the high-transmission filters were not absorbent enough for the streamline analysis to clearly show the point of final convergence. The point of final convergence would be the point that is reached when a filter or curved glass object is maximally absorbent and minimally transmissive, which would be achieved by scaling down the spectral transmission distribution without changing its shape, and it could be this point that observers are matching with the uniform patch element. We can gain a better idea, then, by looking at the results of this analysis for the low-transmission version of the same yellow Glaven under the same white illuminant, shown in Figure 14. Here, we show two sets of matches: The matches to this low-transmission Glaven are red and the matches for the high-transmission version that we just investigated in Figure 13 are blue. While the matches for the low-transmission Glaven are close to the point of convergence, this is to be expected, since the more absorbent a glass is, the more that all colors in the filtered region are forced to the same point and the less variability there is overall in the glass. This means that although observers might also not make a match to the point of convergence for the lower-transmission stimuli, it can look that way at first because even if they match to a different feature of the distribution, this feature will have a color that is close to the point of convergence in the lower-transmission cases by necessity. If we turn our attention to the matches for the high-transmission version of this yellow Glaven (shown in blue in Figure 14), we see that they are not making a match to the final point of convergence, neither in terms of luminance nor chroma, so the convergence model apparently does not explain their matches with the uniform patches. For those readers who are interested, similar plots for the other Glavens in Figure 2 are in the Supplemental Material, where it can be seen that this pattern of results holds for basically every Glaven that we tested.

All of this could mean that observers weigh different features to come to a final estimate for the uniform patch, and components of the White Point, for example, or the point of convergence are part of this estimate, or they give different weights to different parts of the Glaven or make use of a different feature that has components that correlate with the White Point or the convergence point, but to be certain about any of these possibilities would require more extensive manipulation of the different factors in the stimuli, as well as potential eye-tracking experiments. So, while it is unclear what exactly observers are doing when they make a uniform patch match, we will see shortly that observers at least also make use of information from the surrounding background of the scene when making a match with a flat filter.

### Matches made for the white walls scene

**Figure 15.**
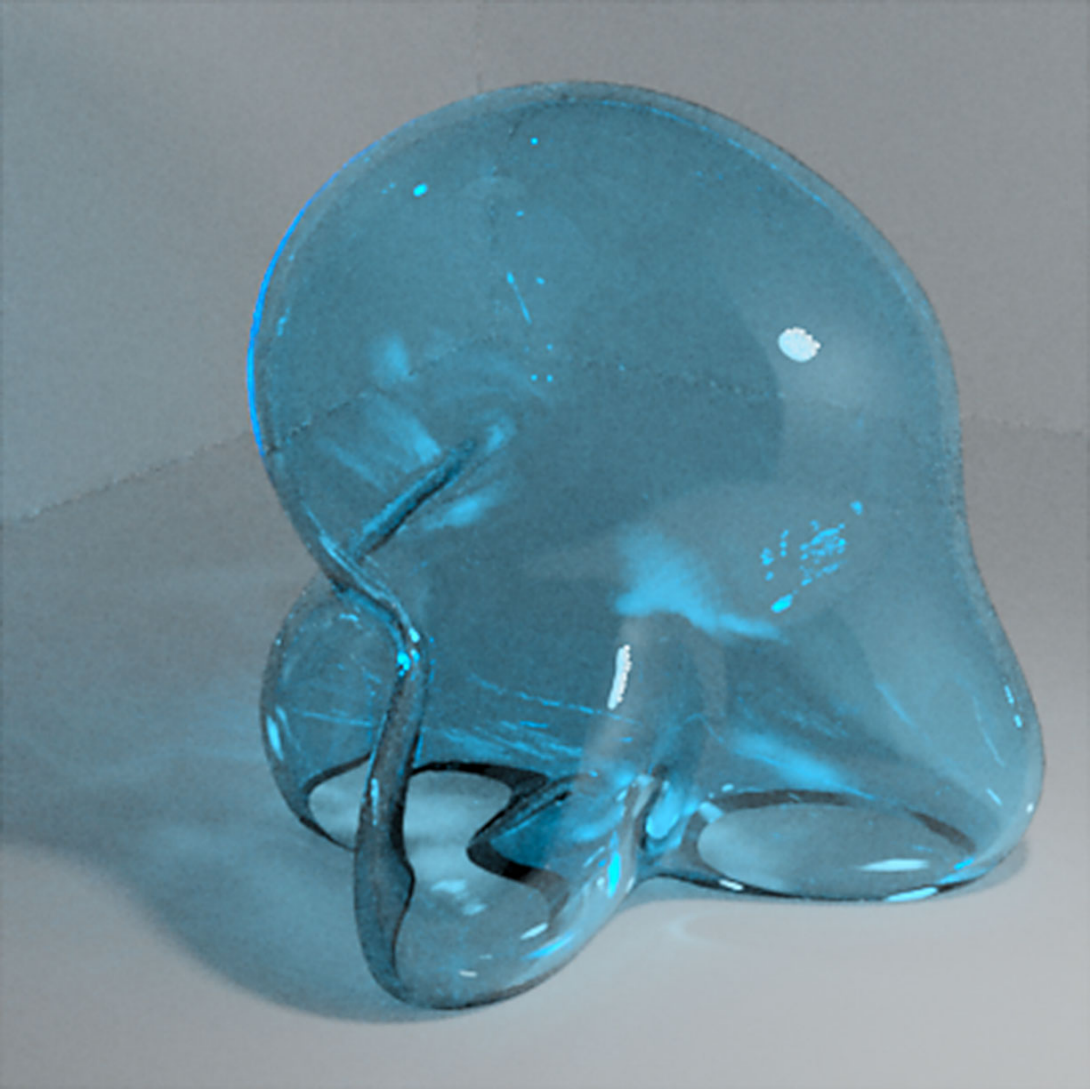
An example of the white walls scene.

**Figure 16.**
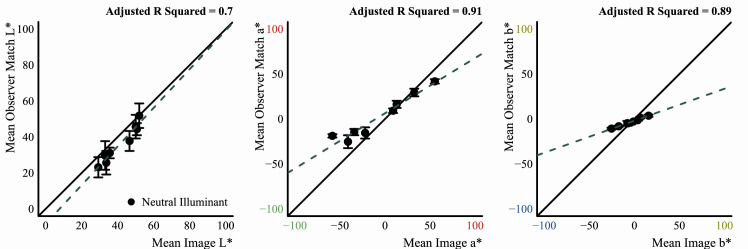
Results of matching experiments with the white background and neutral illuminant. Plotting conventions are the same as those in Figure 9. See the main text for interpretation of the results.

**Figure 17.**
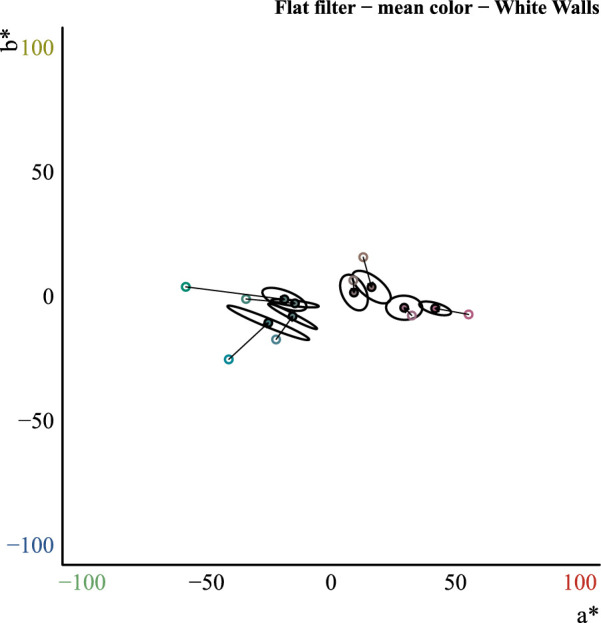
The results from Figure 16, but plotted in the CIELAB (a*, b*) plane, following the same plotting conventions as in Figure 10.

In the case of the white walls scene (an example is shown in Figure 15), we only tested the flat filter matching element. The results can be seen in Figure 16: Now, the mean lightness of matches corresponds closely with the mean lightness of the glass Glaven. The point of this experiment was to test if observers are potentially accounting for the illumination difference between the test scenes and the flat filter renderings. In other words, it tested if observers were performing a kind of color constancy operation. Basically, if we use a scene with a white background and a neutral illuminant, then observers no longer need to discount the illumination difference, and the lightness of their matches might come closer to or even directly match the mean lightness of the region filtered by the glass Glaven. We see that this is the case. This indicates that observers are partly performing a color constancy-esque discounting operation that takes into account the fact that the flat filter is rendered on an achromatic background and under a neutral illuminant. However, all of this only applies for the mean luminance of their matches. In terms of overall mean color, the observers are still not matching the mean color of the Glaven with the mean color of the flat filter. That is made more apparent when the mean colors of the matches are again plotted in a chromaticity diagram, as shown in Figure 17.

However, these results are not enough on their own to determine if observers put more weight on discounting the effects of the background or on the effects of the illuminant, since both are confounded in this test. A more rigorous color constancy style of experiment that independently manipulates both of these factors would be necessary. Regardless, one might presume that for the white walls stimuli, that the overall mean color of the observer matches and the mean color of the Glavens should be closer than in the experiments described above. Looking at Figure 16, we see that only the mean lightness of the two is much closer; the trend of a* still shows deviations from the mean color (larger than before), and b* shows basically the same deviating trend as before. This is not a problem, however. It depends on what information in the scene the observer actually uses. It is not required that they use the mean color, and if they do not use it, then it is not required that it matches exactly, nor is it required that the correspondence between the mean color of the Glaven and flat filter improves as the illumination and walls become whiter. In fact, as the data here shows and as we will see in the section below, the mean color is not what observers are matching. Essentially, the increase in correspondence between the mean lightness of the flat filter match and the mean lightness of the Glaven turns out to be a consequence of another statistic that observers match.

### Statistics investigated for flat transparent filters

The previous two subsections dealt mainly with the relationship between the mean color of the region filtered by the glass Glaven and observers’ matches, where it was found that none of the mean color, the most frequent color, the most saturated color, and the White Point statistic are adequate predictors of their matches. However, other important factors that were determined to work for flat filters could certainly translate to curved transparent objects. In the case of the flat filter matching element, there is spatial variation that the visual system could evaluate when detecting transparency and assigning a color to it, and our analysis of the convergence model has already shown that features related to this spatial variation are shared across the flat and curved case. This feature could be what observers are actually matching. Two measures that relate to spatial variation are the ratios of mean cone excitations (RMCs) between filtered and unfiltered regions, as suggested by Khang and Zaidi (2002a), and the robust ratio model, as suggested by Faul and Ekroll (2011).

**Figure 18.**
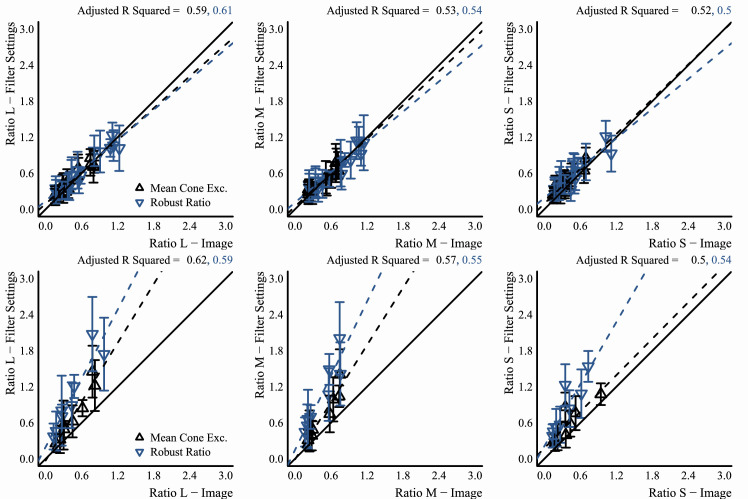
Comparison of the RMC and robust ratio models for our test stimuli with the glass Glaven and the matching flat filter stimulus. For each test scene, we consider each cone type in turn and plot the given statistic for the test scene against the same statistic for the matching filter. In the top row, we show the results for the scenes with the multicolored Voronoi background, and in the bottom row, we show the results for the white walls scenes. Since both statistics are ratios computed from the same units, they are plotted on the same axes as different symbol types (see figure legend). If points lie on the solid unity line, then there is good correspondence between the statistic in the matching filter image and the test scene. The dashed lines show the best-fitting linear regression to the data, where their color indicates the associated data points, and the adjusted $R^{2}$ for each is shown in the same color above each plot.

In Figure 18, we compare the RMC and the robust ratio model for the flat filter matching element and those for the test stimulus with the glass Glaven (see the “Method” section for details on how these ratios were computed). We find an initial good correspondence between the RMC and the robust ratio model for the multicolored Voronoi background test stimuli (results in the top row of Figure 18). In the bottom row, we show the results for the white walls stimuli, where we again find a good correspondence between the values for the glass Glaven and observers’ flat filter matches, although the RMC is now performing better than the robust ratio model. Since the RMC and the robust ratio models are both essentially ratios of means and we have found them to be highly correlated across many simulated variations of our scenes, it is not much of a surprise that they both do similarly well at predicting observers’ responses, but the results for the white walls stimuli already indicate that the RMC has an advantage, since its trend lines run closer to the diagonal unity line, especially for the S cone class. In light of this, we have decided that if given a choice of just these two models, then one should prefer the RMC, not only because it has an advantage for the white walls scenes but also because it is simpler and does not require that the brain internalize extra details about the stimulus.

#### A comparison of the RSD model with the RMC

The robust ratio model is a companion to an RSD model that was shown by Faul and Ekroll (2011) to be capable of explaining observer color matches across pairs of flat filters. In fact, the RSD has seen more attention than the robust ratio model: The RSD was used for predicting a matching filter's color code in Faul and Ekroll (2012) and; Faul and Falkenberg (2015), and it was used to build a perceptually uniform space of filter colors in Faul (2017). In this section, we provide some tests to see if the RSD model is sufficient.

**Figure 19.**
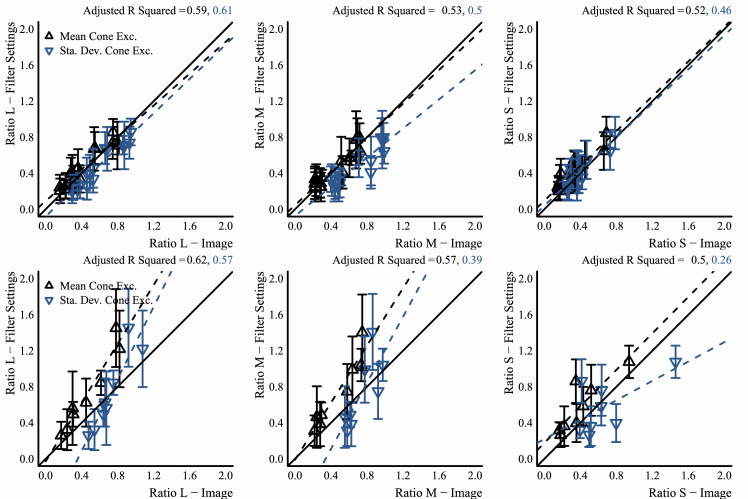
Comparison of RMC and the RSD for our test stimuli with the glass Glaven and the matching flat filter stimulus. Plotting conventions are the same as in Figure 18.

In Figure 19, we compare the RMC and the RSD for the flat filter matching element and those for the test stimulus with the glass Glaven (see the “Method” section for details on how these ratios were computed). We find an initial good correspondence between the RMC and the RSD for the multicolored Voronoi background test stimuli (results in the top row of Figure 19). In the bottom row, we show the results for the white walls stimuli, where we again find a good correspondence between the values for the glass Glaven and observers’ flat filter matches, although the RMC is now performing better than the RSD for S cone stimulation (third Panel in bottom row).

It is important to note that the RMC is not predicting observer matches perfectly for the white walls stimuli, even though it is doing better than the robust ratio and RSD models. While this is true, we show in the remainder of the article that the RMC performs the best, and it is predicting observers’ matches quite well for the majority of stimuli. Also, directly manipulating the RMC via image processing produces substantial changes in the color of a glass Glaven, whereas manipulating the RSD does not change the color in general (shown and discussed later). Rather, the results for the white walls stimuli imply two things: (1) that the RMC does not account for all of the variance and additional factors can be at play (we consider this in more detail in the “Discussion,” but in short, we think that the RMC accounts for the majority of variance) and (2) that specific combinations of background colors and filters lead to a breakdown in the high correlation between the RMC and the RSD that was exhibited in our original set of stimuli (those in Figure 2).

Continuing with our comparison of the RMC and the RSD, in the event that we chose stimuli that ignore particular combinations of background and filter colors that break a potential correlation between the RMC and the RSD, we ran a series of simulations, where we instructed Mitsuba to render our scene, but for many different combinations of transparency, illuminant, and background distribution. We only used these simulated scenes to contrast and compare the RMC and RSD models. We did not do an extra comparison for the RMC and robust ratio model, since they are so similar in form with the RMC doing better, and the RMC was already to be preferred on grounds of simplicity alone. However, in the case of the RMC and RSD, they are equally simple models, and the white walls scenes were deemed insufficient to distinguish the two models. Also, the RSD has seen much more attention and use than the robust ratio model. Hence, the more detailed comparison of the RMC and RSD.

We simulated scenes for lighter and darker transparency distributions, to remain consistent with the experiments detailed above. In particular, we took the four base transparency distributions that were used to render our test scenes with the Glaven (i.e., the spectra that came from the Computational Spectral Imaging group at the University of Eastern Finland; Hiltunen, 2019) and used those as four different illuminant spectral distributions. We then took these same four base transparency distributions and computed a number of linear combinations of them and used those as the spectral reflectance and spectral transmission distributions for the glass Glaven. To get these new transparency distributions, we took combinations of the base blue, yellow, red, and green distributions and computed linear combinations of them using an equation of the following form:

(3) $$\begin{array}{ccc} & & \;\;\;\;\;\;\;\;\;\;\;\;NewDistribution=0.5*LDScale*((\alpha_{RG}*D_{red}\\ & & +\;\left(1-\alpha_{RG}\right)*D_{green})+(\alpha_{BY}*D_{blue}\\ & & +\;\left(1-\alpha_{BY}\right)*D_{yellow})) \end{array}$$

where LDScale is a factor that controls whether the resulting distribution gives a lighter or darker body color; $D_{...}$ is the respective transparency distribution, say, the red Munsell transparency distribution; $\alpha_{...}$ is the multiplication factor that determines the specific linear combination; and the 0.5 scaling factor is used to keep values in the transmission distribution range of [0,1]. We then independently varied $\alpha_{RG}$ and $\alpha_{BY}$ from –1 to 1 in four equally spaced steps, and LDScale was either 0.68 or 1, like in the experiments described in the main article, which in total gives 32 distributions. We then created four additional background textures, in addition to the one that was already used in the main experiments. Three of them had the same spatial configuration as the multicolored Voronoi background that we already used (see Figure 1), but with different color distributions that pushed their means toward red, green, and blue, since the original distribution had a yellow bias. The fourth distribution was an Eidolon-transformed (Koenderink et al., 2017) version of a background texture with the blue bias. Briefly, Eidolons apply a locally smooth, but random, spatial deformation to an image (it can be applied separately to different spatial scales), so that the image is distorted but still retains certain features. It can simulate the appearance of objects in the peripheral visual field (Koenderink et al., 2017), or when they are under water (Dövencioğlu et al., 2018), or what a tarachopic amblyope sees (Koenderink et al., 2017). This Eidolon transformation was done to roughly test if the spatial distribution of the background played a role. The Eidolon-transformed version was also slightly darker. In total, we had five different background textures, since we also used the original background texture with the yellow bias. We then rendered each possible combination of transparent distribution, illuminant distribution, and background texture, giving 640 images in total.

**Figure 20.**
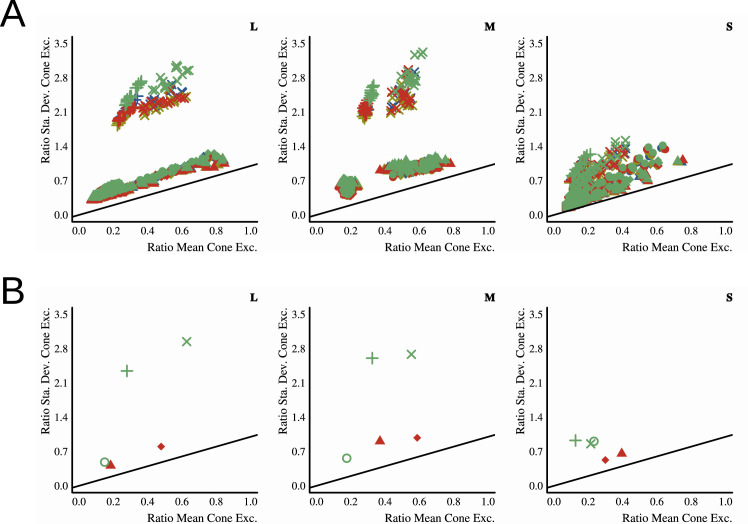
Comparison between RMC and RSD for the simulated scenes described in the main text, computed for each cone class. (A) Values for each cone class are in separate subplots. RMC is on the x-axis and RSD is on the y-axis. Each point corresponds to one of the simulated scenes. The colors of the points denote the color of the illuminant, and the plotting symbols simultaneously denote the color bias of the background texture and whether the transparent object had a lighter or darker body color. Lighter body color: open square—blue background, open circle—yellow background, open triangle—red background, open diamond—green background, cross—Eidolon-transformed blue background. Darker body color: filled square—blue background, filled circle—yellow background, filled triangle—red background, filled diamond—green background, x-symbol—Eidolon-transformed blue background. The solid dark line denotes the unity line. (B) The values for the five scenes that were chosen for testing.

After rendering these scenes, we computed the RMC and the RSD for them, like described above, and looked at the correspondence between the two statistics. This is shown in Panel A of Figure 20. It can be seen that for some scenes, the correspondence breaks down, especially for the L and M cones. We selected five scenes, shown in Panel B of Figure 20, to see if they could help us determine whether observers match RMC or the RSD when making their filter match. Four of the scenes were chosen such that any pair would have either roughly the same RMC or RSD in the M cone class, while the other statistic would be different. The fifth scene was chosen to increase the sample size. It was too difficult to achieve this criterion across all three cone classes; this is why we arbitrarily applied the criterion to the M cone class. In the end, this was sufficient to contrast the two models. Essentially, if two images have the same RMC, but different RSD, then if the RMC model is correct, observers should give the same match for both images, but if the RSD model is correct, then observers should give a different match for each image, and vice versa. With this approach, we end up with a selection of images where only one model at most can make the correct prediction, and so we have a way to differentiate between the validity of the two models. Since some of the scenes that break the correspondence are darker overall, we also made brighter versions by rendering the same scene, but with a more reflective background texture: 1.5 times more reflective for the blue, yellow, red, and green background textures and 9 times more reflective for the Eidolon-transformed background with the blue bias, since it was darker relative to the others.

**Figure 21.**
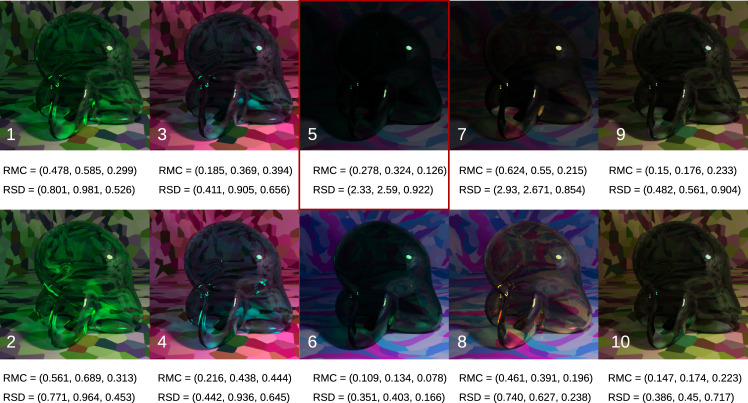
The 10 scenes that we used for a more direct comparison of RMC and RSD via our filter-matching task. The original scenes that were selected from the simulations are in the top row, and the five counterparts with the more reflective background textures are in the bottom row. The images in the third and fourth columns have the Eidolon-transformed blue background texture. RMC and RSD for each scene are shown underneath each image in the following format: (ratio for L cones, ratio for M cones, ratio for S cones). The scene with the red frame (Image 5) was not tested in the final experiment, since it posed too much difficulty for observers in preliminary testing. Note that these images are only rough approximations when shown on the Internet. The images display correctly (i.e., exactly as they were shown to observers) on the Eizo monitor that was used for testing. If you are viewing the document on a tablet or laptop, it may also help to tip the display forward or backward a bit. Also, check the main text that references this figure for details on how to reproduce our viewing conditions. Please be aware that because of compression artifacts that can distort images, especially dark images, we do not suggest using the images in this article as a way to double-check our analyses.

The 10 scenes and the corresponding RMC and RSD values are shown in Figure 21. In the end, we only presented 9 of the 10 scenes to the observers and did the data analysis for only those 9 scenes, since one of the scenes was too difficult for observers. The scene that was too difficult is shown with a red border in Figure 21. We wish to point out that we also do not use illuminants that lie at the extremes of color space, and we are not choosing illuminants with only a few wavelengths or using transmission distributions that only transmit a few wavelengths. Part of this is enforced by how we selected illuminants: The new illuminants had the same distributions as the original transparency distributions that we tested (see Table 1), which were all broadband. The broadband qualities of the illuminants and the transmission distributions can be seen if one inspects the images in Figure 21: In each image, different pairs of complementary colors can be seen in the backgrounds, including other hues, even when viewed through the glass Glavens. This would not happen if we had narrow-band illuminants and highly selective transparency distributions, since they greatly reduce the variance of hues in a scene. So, while we are searching for stimuli where RMC and RSD give different predictions and these stimuli maybe appear “extreme,” we are not choosing stimuli that severely limit the information provided to the visual system, which could potentially cause it to act in unexpected ways that do not correspond with “normal” behavior (although, this is not bad in principle: Illusions are classic examples of limiting information content and eliciting “nonnormal” behavior from the visual system, and illusions have taught us much about the function of the visual system).

One could rather argue, though, that perhaps such illuminants, backgrounds, and transmission distributions occur rarely in the natural world, which would then seem ironic, since we use the “more natural” Glavens to overcome the limitations of flat filter stimuli, but there is also a dual meaning to the term “natural” stimuli. One meaning of “natural” is if all factors that are typically encountered in everyday life are present in the stimulus. In the case of flat filters versus Glavens, these factors would be shadows, caustics, specular reflections, and curved surfaces. A different meaning of “natural” is frequency of occurrence: Certain values of these factors occur more or less frequently in the everyday world (e.g., the curved Glavens occur quite infrequently in the everyday world, but the “curve” factor is present). If bringing more factors into play is not enough to discriminate between equally viable models, then one needs to go an extra step and push the visual system and the models outside their normal operating range by using less frequently occurring stimuli to accept one model and reject another. Essentially, the question should usually be phrased, “Have we explored enough (the possible values) of this perceptual space (the different factors/dimensions) to have a good enough idea of what is going on?” And, as hinted at in the previous paragraph, there is a difference between “nonnormal” and “limited information content.” For example, Images 3 and 4 of Figure 21 that show the bluish Glaven in a pink room might look “nonnormal,” but the information content in the image is not limited or severely reduced relative to more “normal” scenes, like those in Figure 2. As already stated, one can see this by noticing the presence of different complementary colors in the background, even when viewed through the glass Glaven, and other hues are also present. In addition, the Glaven has a clear and defined color, and it does not look like it is the result of “erroneous” visual processing or faulty mechanisms that are not receiving proper stimulation.

Also, note that the images in Figure 21 are only rough approximations when shown on the Internet or in print. The images display correctly (i.e., exactly as they were shown to observers) on the Eizo monitor that was used for testing. One can come close to reproducing our test environment by making a screenshot of one of the stimuli and presenting that on a color-calibrated monitor in a dark room, against a light gray background. Adapting first for 1 min to the light gray background is also recommended. Please also be aware that because of compression artifacts that can distort images, especially dark images, we do not suggest using the images in this article as a way to double-check our analyses.

We showed these nine scenes to three observers and had them make a filter match in the exact same manner as before, except that observers only did four repeats for each image, rather than five, to save a bit of extra time, since observers were rather internally consistent in the previous experiments. Also, this time, after each trial, observers gave a quality rating for their match on an ordinal scale of 1 to 5. It was explained to observers that they should enter 5 if they felt that their match was perfect and that they should enter 1 if they felt that they could not find any satisfactory match, and that they should consider 2, 3, and 4 as equally spaced steps between these possibilities.

The only other major difference from the earlier experiments was that new filter transmission distributions were chosen as the endpoints of the axes that defined the linear combinations observers could make in the flat filter when moving the mouse. We did this because some of these new scenes had glass objects with a body color that was outside the gamut of matches that could be achieved with the selection of transmission distributions used in the experiments described earlier in the article. The Munsell coordinates that corresponded to the new filters (see “Method” section for more details) for this specific experiment are shown in Table 4.

**Table 4.** A new set of more saturated Munsell chips whose reflectance distributions were used in the observer-controlled linear combination that determined the spectral transmittance distribution of the flat filter matching element.


Red	5RP 4/12
Green	2.5G 6/12
Blue	5PB 4/12
Yellow	2.5Y 7/12

However, despite this expanded gamut, one might have concerns that observers could not make adequate matches on our monitor, especially for the RSD values. The concern with RSD values comes from the restrictions placed on totally transmissive and totally absorbing flat filters in earlier studies. If specular reflections are not allowed, then a totally transmissive flat filter will have an RSD of $\left[RSD_{L}=1,RSD_{M}=1,RSD_{S}=1\right]$, and a totally absorbing flat filter will have an RSD of $\left[RSD_{L}=0,RSD_{M}=0,RSD_{S}=0\right]$, meaning that to get RSD values for a flat filter that are greater than 1 is physically impossible, unless the flat filter itself glows in a few spots and emits its own light. Basically, an RSD value greater than 1 means that a flat filter would be increasing the variability of the colors that it is filtering. These physical restrictions on the classical flat filter stimulus would then imply that if RSD were the main determinant of the color of glass, then observers would not be able to make an adequate match for Image 7 in Figure 21, since it has RSD values that are greater than 1. One might also be concerned that the RSD values for Image 7 are also physically impossible and incorrect, but the restriction of RSD to the range of [0,1] only applies for the limited viewing conditions of the original flat filter studies. Once specular reflections, caustics, shadows, and interreflections are allowed, then there can be greater overall variability in the region filtered by the Glaven than in the background.

**Figure 22.**
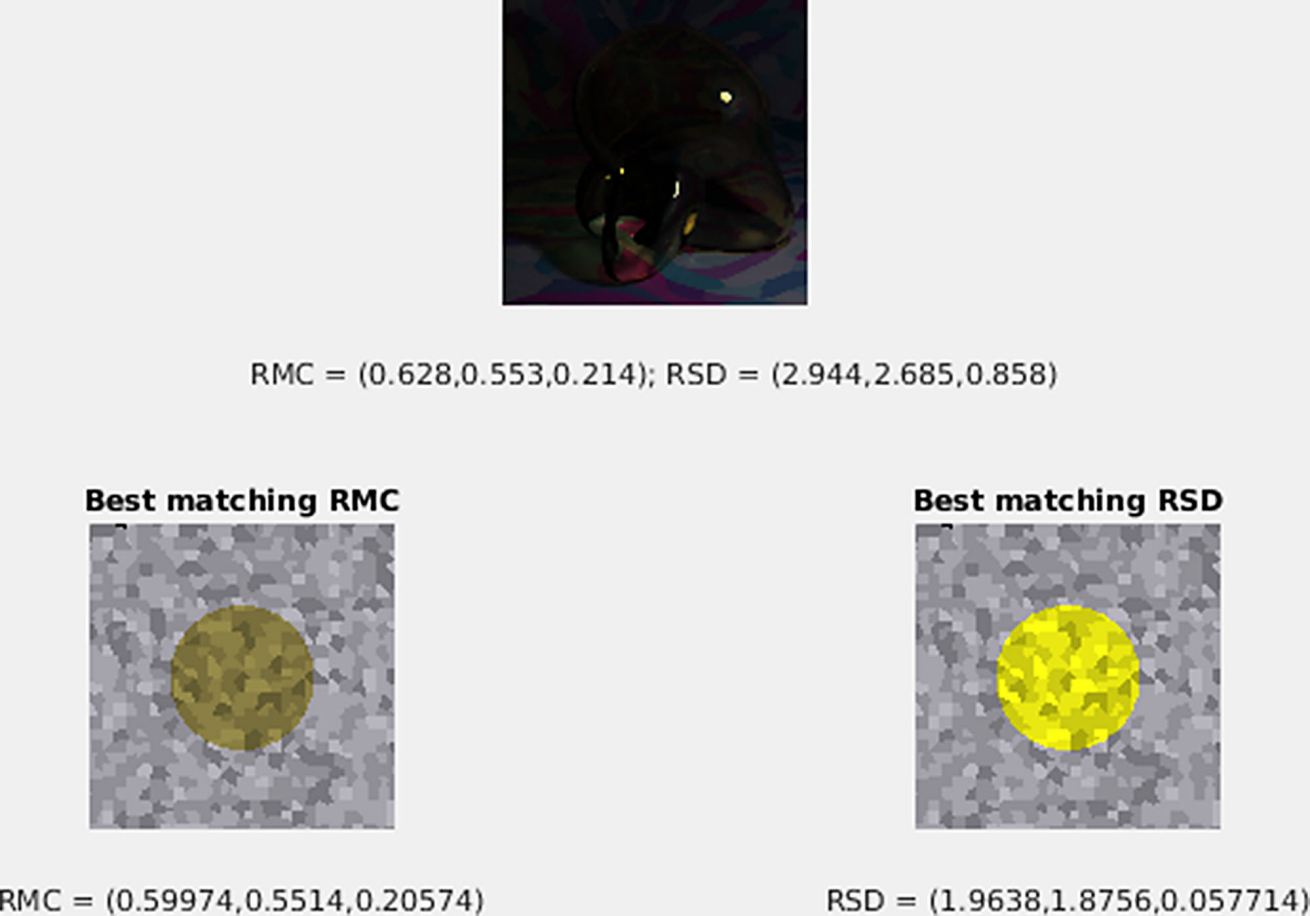
The two filters with the best matching RMC and best matching RSD for image 7 from Figure 21. While our monitor could not go to the “extreme” RSD values necessary for producing the best matching RSD filter for image 7, it can get close and it can be seen that going further would only make the discrepancy between the filter and the Glaven worse. Rather, the best matching RMC filter is more sensible, although this image is peculiar and observers do choose the best matching RMC filter when making a match to it.

As a quality control check, we densely sampled the “flat filter gamut” of our monitor, and from that set, we chose the filters with the best-matching RMC and best-matching RSD for some of the Glavens in Figure 21, especially Image 7. Aside from Image 7, we were always able to find a flat filter with a matching RSD that could be produced by our monitor. In the case of Image 7, the best-matching RSD that our monitor could produce was equal to [1.96, 1.88, 0.058], which is close but not a perfect match. We show in Figure 22 what this filter looks like, as well as the filter with the best-matching RMC. The best-matching RSD filter is clearly a wrong match, and using a monitor where we could push the RSD of this flat filter further would only make the discrepancy between the filter and the Glaven worse. However, the matches that observers make for this image in particular do end up being a bit peculiar. We discuss this in more detail below, when we consider the results from the experiment.

**Figure 23.**
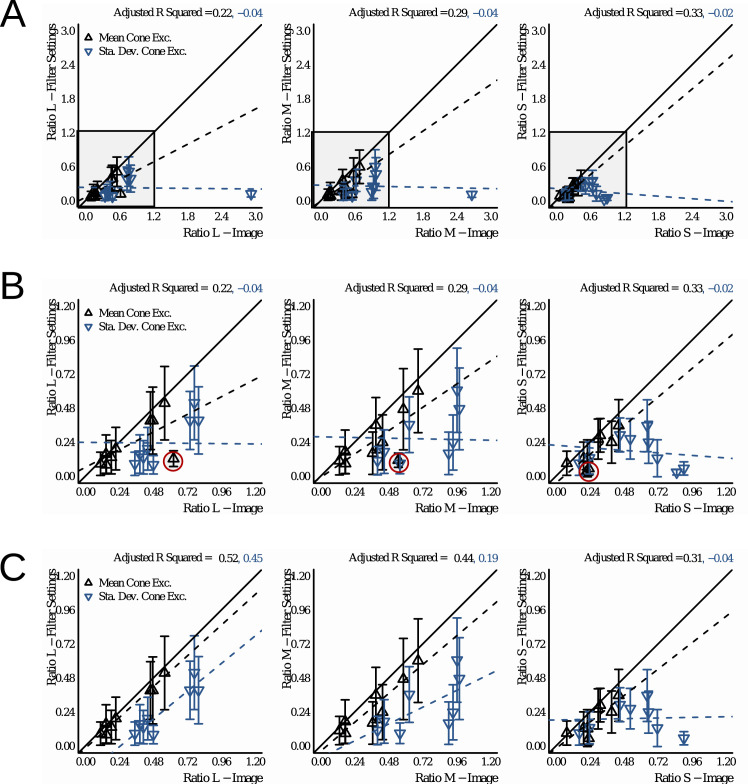
Comparison between RMC and RSD for the nine scenes that are intended to break the correlation between these two statistics (see main text for details). Plotting conventions are the same as in Figure 20. (A) Results for the filter matches of three observers. (B) The same data, but zoomed to focus on the shaded, square region in Panel A. The best-fit lines are the same as those from Panel A, in that they are still fit for the image with very high RSD in Panel A. The RMC data for one image are circled in red. This is the same image with the high RSD in Panel A. It is an example of an image with a filter setting that has lower RMC than expected given the value of the RMC in the actual test image and the slopes of the best-fit lines. (C) The same zoom as in Panel B, but with the data for the red point removed, since this also corresponded to the image with the lowest quality rating.

Observers’ average quality ratings are shown in Table 5. In Panel A of Figure 23, we show the results for the nine scenes that we tested with the three observers. At first glance, it can be seen that RMC is best at predicting the settings in the S cone channel, and that it is better overall than the RSD but not perfect.

**Table 5.** Average quality ratings (±*SEM*) of observers for their matches to the stimuli that decorrelated the RMC and RSD. The image numbers correspond to the labels in Figure 21. See the main text for more details.

Image	Avg. quality	*SEM*
1	4.083	0.52
2	3.667	0.722
3	4.583	0.144
4	4.25	0.661
6	4.25	0
7	2.333	1.041
8	4.25	0.661
9	4.25	0.5
10	4.667	0.289

**Figure 24.**
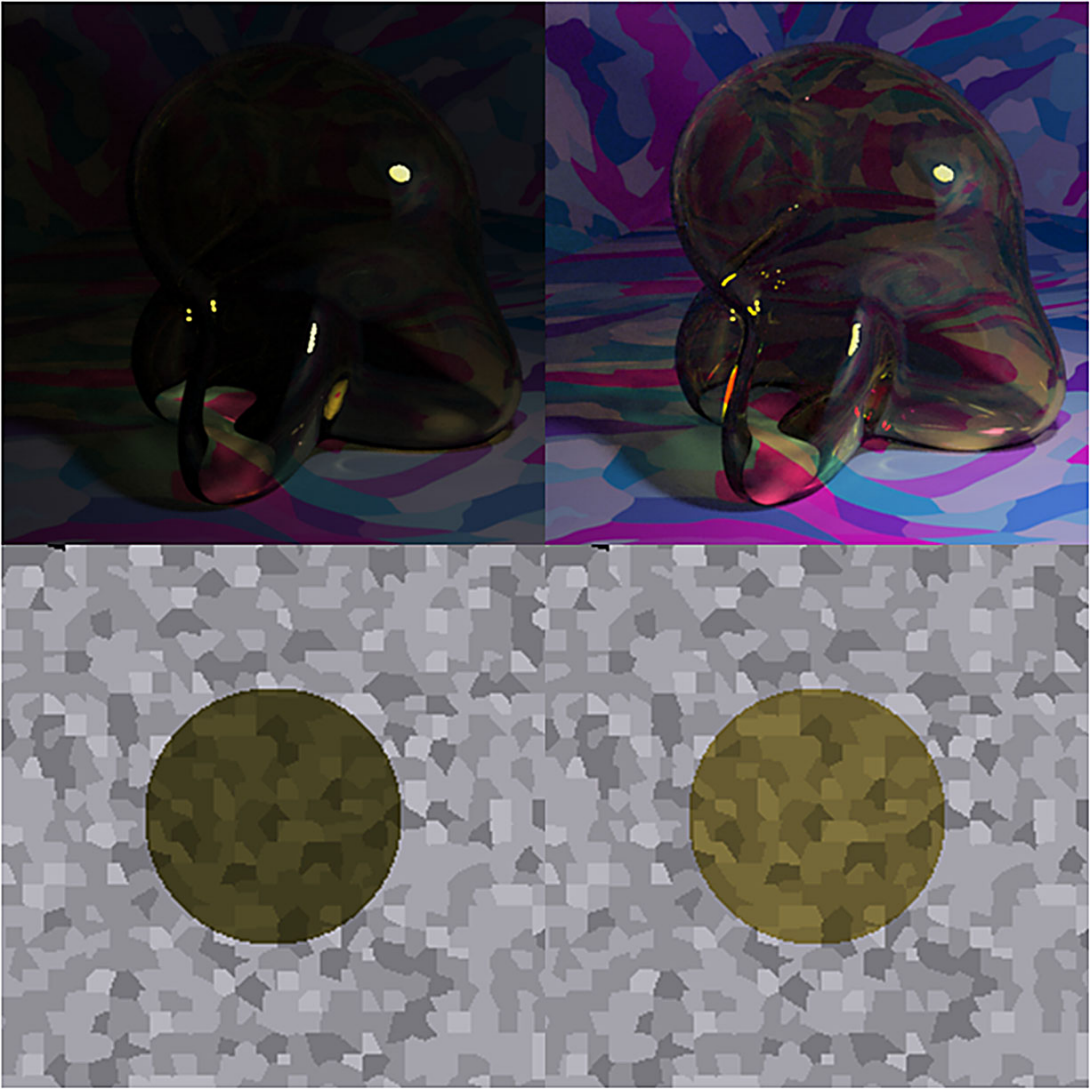
Depiction of the test image whose data are circled in red in Panel B of Figure 23. The image whose data are marked in red is on the top left, and its counterpart with the more reflective background texture is on the upper right. The average settings of observers left in the filters when they were satisfied with their match are shown in the bottom row, where the filter on the bottom left is the average match to the image on the upper left, and the filter on the bottom right is the average match to the image on the upper right. Note that these images are only rough approximations when shown on the Internet. The images display better on the Eizo monitor that was used for testing. If you are viewing the document on a tablet or laptop, it may also help to tip the display forward or backward a bit. Please be aware that because of compression artifacts that can distort images, especially dark images, we do not suggest using the images in this article as a way to double-check our analyses.

In Panel B of Figure 23, we zoom into the shaded, square region marked in each subplot of Panel A. The fitted lines are still for the data shown in Panel A, including the image with very high RSD in the L and M cone channels. We see that RMC is doing better overall, but one image, circled in red, is always matched with a filter that has a lower RMC setting than the RMC found in the image. This is the same image that has the high RSD value shown in Panel A (by design; see explanation of paradigm above), but it is also Image 7, which received a low-quality rating from observers (see Table 5). Observer filter matches for that image have a very low RSD in comparison to the RSD that is actually in the image. If it is possible that this one image drives the poor trend for RSD, then we can temporarily remove this image from the analysis and look at the results for the eight remaining scenes. That is shown in Panel C of Figure 23. We see that this improves the trend for RMC and RSD, to the point that RMC could be considered a good predictor of the data, whereas RSD is still doing poorly for the M cone channel and especially the S cone channel. However, it is also interesting to actually look at the tested image, its counterpart with the brightened background texture, and the average filter settings that observers left in their matches. This is shown in Figure 24.

In Figure 24, we see that although observers gave a low-quality rating for the filter matches to the glass object in the scene on the upper left, they actually make a setting that is reasonable: What appears to be a dark yellow-green glass is matched to a dark yellow-green filter. We show the counterpart image with the more reflective background texture on the right to show that observers make a similar match in terms of hue when the intensity of the scene is greater overall. This indicates observers might just be hesitant when making a match under darker conditions, and although they are making a reasonable match, they just do not trust themselves and put a low-quality rating. With a bit of confidence training, observers might put higher-quality ratings for such images and then future investigations of these types of images, where both RMC and RSD do not predict the matches that observers make, might provide further insight into the mechanisms that determine the color of a transparent object.

**Figure 25.**
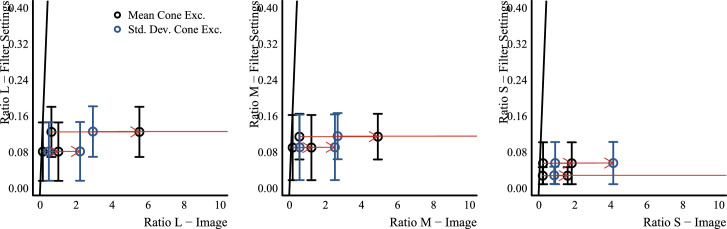
RMC (black points) and RSD (blue points) for the image in the top-left Panel of Figure 24, plotted in their original form from Figure 23 (tails of arrows) and in a revised form (tips of arrows) where dark pixels have been excluded from the analysis (see main text for details). The arrows always point to the right, further away from the unity line (black diagonal line; visually stretched because of unequal axis lengths to zoom in on the data). This means that excluding dark pixels makes RMC and RSD predictions worse. Some points even shift so far to the right that they reach predicted values of 10 or larger. They are not plotted here to keep the majority of data in view, but part of the red arrow that connects them is still visible.

One could expect, though, that observers might ignore regions of the image that are relatively too dark (i.e., a weak signal), and only those regions that pass a certain threshold are evaluated when determining the color of a transparent object. If that were the case, then the circled red point in Figure 23 might only be a poor prediction of observer responses because dark pixels were not excluded when the RMC and RSD were computed. To test if this were the case, we recomputed the RMC and RSD for that point (i.e., the image on the top left of Figure 21) and again excluded pixels that were less than 5% of the maximum luminance in the glass Glaven (excluding specular highlights when computing this threshold), just like when we excluded dark pixels in the convergence model analysis. The results of this recomputation are shown in Figure 25, where it can be seen that excluding dark pixels actually makes the predictions in both cases worse; sometimes predictions become worse enough that they shift very far to the right, reaching values of 10 or larger (not plotted to keep the majority of data in view, but part of the red arrow that connects them is still visible). This indicates that observers do take the whole scene into account, including dark regions, and that there might be a lack of, or at least a limit to, “transmittance constancy” (i.e., glass under dim illumination looks “darker” or more absorbent, even though it might be highly transmissive). The matches in Figure 24 support this conclusion.

From these simulations and these data, we find that observers’ settings do diverge when RMC and RSD are decorrelated in test images, but in a slightly complicated way. The data support RMC over RSD as a likely image statistic that observers use to assign color to a transparent object, yet there are images that contend with this conclusion, such as the red point in Panel B of Figure 24. These images could indicate that there is another statistic that trades off with RMC for determining the color of a transparent object. Further work will need to be done with images of this type.

## Discussion

Our goal was to understand what determines the perceived color of a curved transparent object, and we have found further evidence that the percept is strongly driven by the ratios of the mean cone excitations between the filtered and unfiltered regions of the image (RMC), as suggested by Khang and Zaidi (2002a). The RMC could be how the visual system estimates the magnitude and point of convergence affected by a transparent object, since we have found that the convergence model holds in a modified form for curved transparent objects. However, additional statistics might be at play or might trade off with the RMC, especially since there are more factors at play for curved, transparent objects. Indeed, as discussed in the convergence model subsection of the “Analysis” section, the visual system might also estimate the magnitude and point of convergence via Gestalt principles. On the other hand, we saw that the Affine convergence model begins to degrade in the CIELAB color space, indicating that the signals that encode the magnitude and point of convergence begin to transform in nonlinear ways at later stages of visual processes. This means that an alternate way to understand the relevance of the RMC and convergence model is that glass objects produce a pattern in the initial visual input that is preserved under topological deformations of the space and is later extracted by the appropriate mechanism. In other words, the visual system might not actually compute the RMC to determine the color of glass, for example, but the RMC statistic quantifies a regularity that is present in the initial cone activations that is preserved under transformations imposed by later stages, until this regularity is finally analyzed by the relevant mechanism. The nature of these mechanisms and the role of other factors, as well as how eye movements could be used to collect samples of the RMC and other features, remain open avenues of investigation for future researchers.

Interestingly, results for the uniform patch settings still defy a complete explanation. When using the uniform patch, observers are making a very asymmetric match between objects in two different modes of perception (Katz, 1911; Beck, 1972; Giesel & Gegenfurtner, 2010). The patch appears like a flat Lambertian surface or a piece of paper, while the glass Glaven is curved and has a body color and various physical properties that the patch cannot emulate. This could partly explain the discrepancy and deviations from the mean color seen in Figure 9. Although, we should point out that observers never stated any difficulty with any of the instructions or with either matching element, and all observers finished the experiments in roughly 45 min. More interestingly, the White Point statistic does an excellent job of predicting the chromaticity of the uniform patch match, but it is not capable of explaining the lightness of the match. As a result, it alone is not capable of explaining observer behavior in the task.

**Figure 26.**
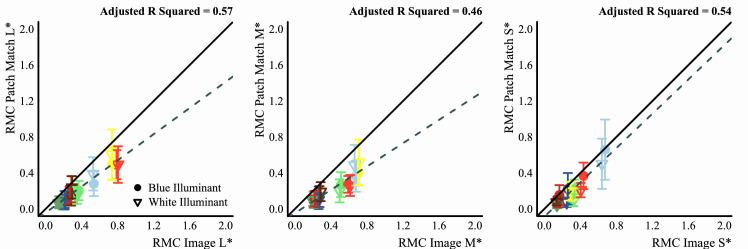
RMC for the uniform patch compared to the RMC for the glass Glavens from Figure 2; see main text for more details. Plotting conventions are the same as in Figure 12.

An additional interesting aspect of the uniform patch is that one can technically calculate the RMC for it, by putting its LMS activation in the numerator and the LMS activation of its surround in the denominator. It is not possible to compute the RSD for the uniform patch, since there is no spatial variation in the patch or its surround. In Figure 26, we show the result of comparing the RMC for the uniform patch to the RMC of the glass Glavens in Figure 2. Although there is a relatively decent trend in each cone class, the RMC for the uniform patch does not match the RMC for the glass Glaven, so how exactly observers compensate for the relative differences between the two stimuli and make a mapping from one class to the other is still unclear.

The use of a uniform patch as a means to determine the perceived color of a transparent object also raises some other interesting points. First, it is important to keep in mind that it is not the case that the matches with the uniform patch could be “wrong” and that those with the flat filter could be “more accurate” or “right.” They are just different, and observers are using different strategies to account for those differences. The original intention of the uniform patch-matching element was to see if observers can reduce the appearance of a transparent object to a single point in color space (i.e., a single color), since we certainly see a red glass object as “red” with a specific and distinct color. Essentially, observers are making a map between transparent colors and uniform Lambertian colors. Regardless of whether observers use a uniform patch or a flat filter, they make matches that to us (the authors) look sensible, but the actual settings for these two types of matching elements do differ in their color properties. This is reminiscent of the comment by Xiao and Brainard (2008) that there may not actually be a single well-defined color for all 3-D objects.

It could also be the case that the uniform patch element has led to a situation that is impossible to analyze. Consider that we tested different statistics, in different color spaces, and we could potentially use other masks to restrict our analysis to different subsets of the Glaven or the scene. The question, then, is “which combination of color space, subset, and statistic is correct?” The combinatorial possibilities suddenly explode, and many combinations could produce the same exact result, potentially leaving one hopeless to determine what is actually going on. The possibilities even include the case that observers might find the uniform patch element to be too difficult to use, and they just punt on the issue and make a match to a single pixel in the Glaven. Or, it could be the case that observers are comfortable with the uniform patch, but each of them uses a specific, but different, strategy to deal with the discrepancy in the appearance of the Glaven and the patch, and this variability in strategies would mean that there is no single “general model” that we can apply to explain their behavior. While all of this is theoretically possible and such types of questions are technically a concern for all vision science experiments in general, we also have to consider what is most possible in the context of what we already know about the visual system and the different tasks that observers are able to accomplish. First, it is quite a common task in interior design to “match” or “coordinate” the colors of different materials: The color of the walls might need to match the color of a glass vase, which sits on a table that should have a color that is “slightly accented” to provide an extra ambiance to a room. Ignoring the complications that usually arise from communication issues (e.g., when the customer complains that they wanted “a shade of red, but not that shade of red”), if the customer and the interior designer can sit in the room together and search through a palette of colored wallpaper patches and choose the one that best matches the glass vase on the table, then they have performed a task that is very similar to what we tested in this article. As stated earlier, this task is also of philosophical interest (Wittgenstein, 1978). With respect to difficulty, our observers said that our task with the uniform patch made sense and did not provide them with any difficulty. This further indicates the task is natural, is common, and has real-world implications (e.g., less disagreements between partners when decorating their dining room and fewer complaints from the customers of the interior designer), so the idea that the task is too difficult or senseless has to be given less weight. Considering that and considering what we know about the neural structure of the visual system, then the idea that observers match a single pixel becomes incredibly unlikely, since our current best model of the visual system is that it is a statistical processing machine that takes various factors and parts of the image into account for tasks, not just a single pixel (Attneave, 1954; Barlow, 1961; von Neumann, 2012; Knill & Richards, 1996; Rao & Ballard, 1999; Simoncelli & Olshausen, 2001; Doya et al., 2006; Yuille & Kersten, 2006; Geisler, 2008; Trommershauser et al., 2011). Of course, this model will be refined and potentially replaced in time, but it is unlikely to be replaced with a model that says we only consider single pixels. In fact, one way in which the current model of the visual system is being refined is to account for individual differences: It is actually the case that strategies can vary across observers for the same image and task, and methods for quantifying and evaluating these variations in strategies are a subject of current investigation (Toscani et al., 2017; Wilmer, 2017; de Haas et al., 2019; Linka & de Haas, 2020). However, the variability shown for the patch matches in Figure 10 does not suggest that observers are using very different strategies. For each Glaven, our observers’ uniform patch matches end in the same region of color space. Of course, the overall variability for the uniform patch matches is larger than the variability of matches made with the flat filter, but not much larger, and this slightly increased variability does not mean that a general model that can explain their uniform patch matches is lacking. Rather, it could imply that each observer weighs and integrates different factors of the Glaven, and some observers might put more weight on the caustics, while others put more weight on the colors at the center of the Glaven, all of which can be tested. Lastly, the question of which color space, which statistic, and which subset of the image to use is always part of the process when searching for viable color vision models to explain behavior in complex scenes. It certainly makes the problem difficult, but not impossible. One needs to use many different techniques and manipulations, such as eye tracking, renders of different scenes, image manipulations, real-world scenes, comparison across illumination conditions, moving stimuli, recordings of neural activity, and so on. By comparing and contrasting the results of these different kinds of experiments and considering everything in the context of the broader model of the visual system, one can have a better idea of which model is most likely and can use prior knowledge to assign more or less weight to different models that give the same result. For instance, this is how we were able to differentiate between the RMC, robust ratio, and RSD models. While our techniques and methods were not sufficient to find an acceptable model for the uniform patch experiment, further work by future investigators will probably do so.

One other curious possibility for the uniform patch matches concerns the comparison of those matches with the pattern of convergence for each glass Glaven (see the end of the “Matches made for the multicolored Voronoi background” subsection of the “Results” section and the “Supplemental Material” section). There we found that the uniform patch matches do not correspond with the point of convergence in general. However, this was assuming that the colors of transparent objects can be appropriately represented in the MB-DKL color space. Since the colors of uniform patches and the colors of transparent objects certainly lie in distinct perceptual spaces, then it could also be the case that the sizes of JNDs and the transformations required to reach perceptual uniformity in both spaces differ considerably. In other words, a small shift in the convergence point for a transparent object could correspond to a large shift in color, which might then map to a large shift in the color of the uniform patch, leading to the discrepancies that we saw when comparing the uniform patch to the pattern of convergence. This is also an area for further work.

If we return our attention to the flat filter matching element, then we find that the mean color of the matched filter does not correspond to the mean color of the glass Glaven. This is initially interesting because there is reduced discrepancy between modes of perception for the curved, glass Glaven and a flat transparent filter. In other words, to match a flat piece of glass to a curved glass object is probably easier than to match a piece of paper to a curved glass object. Upon further inspection, we have found that it is the RMC that is most likely what observers are matching, and it is the best predictor to date of the matches that observers make. Considering this, it would be worthwhile to take the approach of Faul (2017) and build a perceptually uniform color space of transparent colors with the RMC as the foundation. However, it is important that such a space account well for both flat filters and curved transparent objects, so further work will need to be done to determine any other factors at play, aside from the RMC. It is important to also point out that the RMC inherently implements a discounting operation, by comparing “background” colors to “foreground” colors (i.e., colors in a region of interest), which helps it be the best predictor across different background and illuminant scenarios. The relationship between color constancy and ratios of cone absorptions, computed in a fashion similar to the RMC, is covered elsewhere (Zaidi, 2001), but since transparent filters share some characteristics with spotlight illuminants (Khang & Zaidi, 2004; Dojat et al., 2006; Knoblauch & Dojat, 2003), it is sensible that the visual system would reuse strategies. However, as stated in the “Matches made for the white walls scene” section, whether observers place more focus on discounting the effects of the background colors or the effects of the illuminant is still an open question for curved, transparent objects. Regardless, it may not be too much of a stretch to say that transparent objects are like “embodied light” (Knoblauch & Dojat, 2003). Although, it is necessary to remember that transparent objects still have a number of their own individual quirks that distinguish them from spotlight illuminants, and there may be more than the RMC at play for curved, transparent objects.

**Figure 27.**
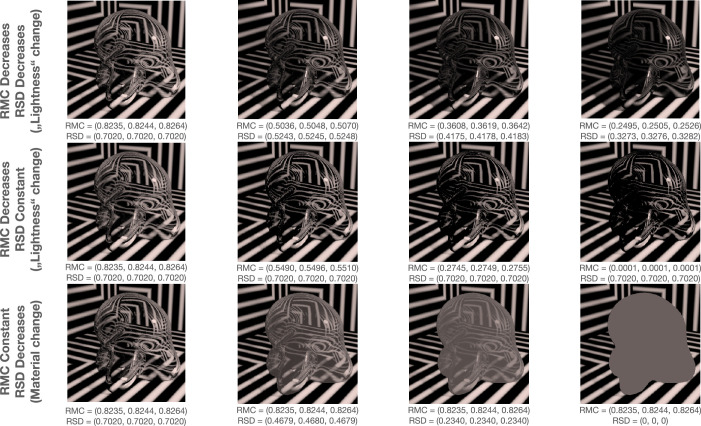
Result of independently manipulating the RMC and RSD via image manipulations, in comparison with changing the transmission distribution in a physically based manner within the Mitsuba rendering system. Each row shows the result of a different manipulation. All rows start with the same image on the left: a maximally transmissive, clear glass Glaven in a scene with a sinusoidal grating texture on the walls and floor. This achromatic scene was chosen to provide more variability in the types of scenes we investigated in this article. Row 1 is for the physically based manipulation, row 2 is for the result of forcing the RMC to decrease via image manipulations while keeping RSD constant, and row 3 is for the result of forcing the RSD to decrease via image manipulations while keeping RMC constant. It is apparent that manipulations of RMC correspond to changes of “lightness,” whereas manipulations of RSD correspond to changes in the quality of the material (e.g., how “cloudy” the glass is) or the material type (i.e., transparent vs. translucent vs. opaque).

But, does this now mean that the RSD is to be discarded and considered irrelevant? Not at all. Rather, we have only shown that it is not what observers are using to assign a color to glass. We can gain a better notion of the role that the RSD plays by manipulating an image of a Glaven, so that we independently modulate RMC or RSD and compare that to what happens when the actual physical transmission distribution of the glass Glaven is changed in the Mitsuba rendering system. Consider Figure 27. In this image, it is apparent that manipulations of RMC (row 2) cause the glass Glaven to look darker in almost the exact same way that reducing the transmission factor in a physically based manner does (row 1). In contrast, manipulations of RSD cause more of a change in material quality, while having little, if any, effect on the “color” (i.e., it does not appear to become darker, at least nowhere near the degree that is seen in rows 1 and 2). Considering this, we now believe that instead of being used for the color of the glass, the RSD may be used by the visual system to evaluate the translucency or “cloudiness” or “clarity” of the glass. It may also be relevant for detecting a transparent object and to then classify it as “transparent,” “translucent,” or “opaque.” In other words, we do think that the RSD is relevant for the perception of transparent objects, just not in the manner that it was originally intended. More work is also needed here, and this is another path of investigation open for future researchers.

Another benefit of the RMC versus the RSD and the robust ratio model is that it is more resilient against accidental inclusion of specular highlights or shadows. For instance, if one computes these statistics for the full Glavens in our 16 original test images, shown in Figure 2, and compares that to the result of computing them with a mask that excludes specular highlights, then each statistic changes by the following percentages on average (averaged across the 16 scenes and across cone classes):

**Table:** 

	RMC	RSD	Robust ratio
Exclude highlights	4.8% ± 1.3%	15.7% ± 6.7%	10.5% ± 1.2 %

This shows that the RMC estimate changes less if the visual system accidentally includes or excludes a few colors that “should” or “should not” be part of the computation, which is something that could happen if objects, the illuminant, or the observer is in motion, or if the observer is momentarily distracted while scanning the scene with eye movements. It means that if the visual system were to use the RSD or robust ratio, then the chance is greater that the color of an object could change or modulate in such circumstances, which is undesirable. Although such “accidental” changes in color could be something that actually happens (it remains to be tested), we think that it is unlikely, considering the stability of perception that we all depend on under normal and sober viewing circumstances. In addition, this extra stability for an RMC estimate means that an observer could ignore or include, at will, some image features that contribute to the complexity that the original convergence model is not able to capture. As we mentioned in the “Results” for the convergence model, this extra complexity could be irrelevant for the color that an observer assigns to an object and they merely ignore it, allowing them to reasonably use the same mechanisms for flat filters and curved glass. It of course needs to be tested if observers ignore this complexity, but the RMC's resilience to the inclusion/exclusion of specular highlights also helps it generalize better to the case of a curved transparent object.

On a different note, Richards et al. (2009) have claimed that if one restricts themselves to the Metelli model of flat transparent filters, then the model can state that certain filter and background combinations can lead to a filter with a color that is not physically realizable in the standard CIE1931 xyY color space. They call these “imaginary colors.” Considering this, it may be the case that our stimuli do not push the limits of transparent colors, where observers might not be able to make a satisfactory match with a uniform patch. However, the CIE1931 xyY color space is not exactly the best space for representing “transparent colors,” especially since it is a color-matching space for flat uniform patches viewed under very restrictive conditions. In other words, it is not a color appearance space (Zaidi, 1992). A core aspect of the approach presented in Richards et al. (2009) is that the region of the transparent filter can be scissioned into two components: a background Lambertian surface with an associated RGB value and the overlying transparent filter with a similarly associated RGB value. However, there is no reason to assume that the visual system represents the colors of transparent filters in the same perceptual color space as Lambertian surfaces (i.e., as raw RGB values). Stated differently, is the “red” of a transparent filter the same as the “red” of a piece of paper? All of this starts to bring out the complexities of comparing colors that are in different modes of appearance (Katz, 1911; Beck, 1972). Consider that the flat filter is essentially a “uniform patch” for transparent objects: It has none of the specular highlights, shadows, or caustics that appear with the glass Glaven, much like the uniform patch has no shadows and none of the curvature that a typical Lambertian object would have. Yet, to actually perceive the filter as transparent, it needs to be placed over a variegated background, like the achromatic Voronoi background that we used, so that the color of the filter is tied in some way to the pattern that it covers. Otherwise, it will look no different from a uniform patch (unless the filter is tilted on one edge, for example, but that also introduces spatial variations in color). If we instead place the filter over a multicolored background, then we quickly notice that we essentially return to our original problem: What is the specific color of a transparent flat filter (Wittgenstein, 1978)? How does the brain derive it from a distribution of cone excitations? Essentially, the color of a glass object depends on extended spatial relationships, which are not encoded by a single point in the CIE1931 xyY color space and so the color of the flat Metelli filter should not be represented as a single RGB value or as a single point in the xyY color space. In other words, if an observer can match a uniform patch (an RGB value) to a flat filter or a glass Glaven, this does not mean that we have found the “specific color” that corresponds to the glass Glaven (or even that of the flat filter!; as we mentioned in the “Method” section, when we discussed the reason for using the uniform patch match element), although the uniform patch match will certainly be informative about what information is decisive for the color of glass. Stated another way, the colors of different materials do exist in their own individual classes, and to specify their color explicitly requires representing them in those specific spaces, much as Beck (1972) and Katz (1911) suggested. Interestingly, despite this, observers can make mappings between the different classes, as we have shown with the uniform patch settings in this study (see also Giesel & Gegenfurtner, 2010; Xiao & Brainard, 2008), even though the “RGB” of the uniform patch is not the same thing as the “RGB” of the transparent filter. The point of this is to emphasize that the colors of transparent objects are not “imaginary” as Richards et al. (2009) suggest. They can be physically realized but must simply be represented in another space, such as an RMC space.

Our results stand in contrast with earlier work on the color of Lambertian and glossy objects, such as that by Giesel and Gegenfurtner (2010), Toscani et al. (2013a, 2013b), and Granzier et al. (2014). This body of work found that when provided with a uniform patch-matching element, observers match to the most luminant region on the body of a Lambertian object and to the most luminant region (excluding the highlights) on the body of a glossy object. Xiao and Brainard (2008) came to similar conclusions: They found that observers compensate for the specular highlights on glossy objects, when they are asked to change the color of a matte sphere to match that of a glossy test sphere. However, in these cases, the results are more readily interpretable. The modes of appearance for a uniform patch and a Lambertian object are similar in the same way that the flat filter is similar to the glass Glaven, so the conclusion that the color of a Lambertian object is determined by its most luminant region makes sense and is a good strategy, since the visual system will have a strong signal to work with from the most luminant region. In the case of glossy objects, that observers are matching to the most luminant region, not including the highlights, indicates that they realize that the highlight color is not directly informative about the color of the glossy object and that they can compensate for the effects of specular reflections. We suspect that the different results that we find for curved glass objects and the flat filter are probably due to transparent objects being an example of volume colors (Katz, 1911; Committee on Colorimetry of the Optical Society of America, 1953; Beck, 1972).

Based on our results, we feel that the following topics are worthwhile avenues of investigation:

(1) The influence of the color of specular highlights and caustics
(2) The role of Gestalt principles and their interplay with the RMC
(3) The potential role of RSD in determining whether an object is transparent or opaque
(4) Building a perceptually uniform transparent color space that is based on the RMC and any other relevant factors
(5) Comparing shifts in a perceptually uniform transparent color space to the corresponding uniform patch matches
(6) Manipulating the thickness of glass and the transmittance factor to determine how well observers can disentangle these two factors, since they counterbalance each other in terms of optical effects
(7) The potential role of the RMC and/or the RSD in detecting a transparent object
(8) How observers use eye movements to sample the scene to come to an estimate of the RMC and/or the point and magnitude of convergence
(9) The influence of object shape
(10) The transition point at which an object absorbs so much light that it looks glossy, rather than transparent/translucent, and to see how well the RMC predicts the color across this range of material variation
(11) What exactly is happening when observers make a match with the uniform patch

There are certainly other items worth investigating for transparent objects, but based on our results, these seem to be worthwhile low-hanging fruit.

## Conclusion

Observers readily answer the question, “What is the color of this transparent object?” regardless of whether the matching element is a uniform patch or a flat transparent filter. In both cases, their responses differ from the mean chromaticity of the Glaven, as well as its most saturated color and its most frequent color, while the White Point almost captures the uniform patch match but falls short of explaining the lightness of their settings. At least for a flat filter matching element, observer responses are the result of a color constancy-esque discounting operation. More accurately, we can say that the ratios of the mean cone excitations (RMC) between the filtered and unfiltered regions are most likely what observers are matching, and it could be what they use to extract the point and magnitude of the convergence induced by a transparent object. However, other sources of information could be at play, which might correlate with or trade off with the RMC, and more work will be needed to determine their effects, as well as any Gestalt principles at work and how eye movements are used to sample the relevant information. At the least, though, our results support the conclusion that the RMC has a substantial effect on the color of a transparent object.

## Supplementary Material

Supplement 1

## Footnotes

1 If thinking about the “dye” that was used to tint the color of a piece of glass is posing difficulty, then consider dripping watercolor paints on paper or adding a green energy drink to water or spilling tomato sauce on white clothes. All of these count as “dyeing” and have the same final effect: The object takes on the color of the dye. All observers in the experiment did not require such an explanation and performed the task without issue.
