# Supplementary material for: The color appearance of curved transparent objects
Source: J Vis. 2021 May 19;21(5):20. doi: 10.1167/jov.21.5.20 (PMC8142700; doi:10.1167/jov.21.5.20)

The color appearance of curved transparent objects - Supplemental Material

Robert Ennis
Justus-Liebig-Universitaet Giessen
Department of General Psychology, Giessen, Germany
http://www.uni-giessen.de/fbz/fb06/psychologie/abt/allgemeine-psychologie/bapl
Robert.Ennis@psychol.uni-giessen.de

Katja Doerschner
Justus-Liebig-Universitaet Giessen
Department of General Psychology, Giessen, Germany
http://www.uni-giessen.de/fbz/fb06/psychologie/abt/allgemeine-psychologie/bapl
Katja.Doerschner@psychol.uni-giessen.de

Here we show plots comparing the uniform patch matches with the pattern of convergence for 14 Glavens from Fig. 2 in the main text. The other 2 Glavens are analysed in the main text of the manuscript, under the "Matches made for the multi-colored Voronoi background" sub-section of the "Results" section. Please see the main text for the reason for this analysis and for an explanation of the plotting conventions used.

Figure 1: Low transmission blue Glaven under the blue illuminant.

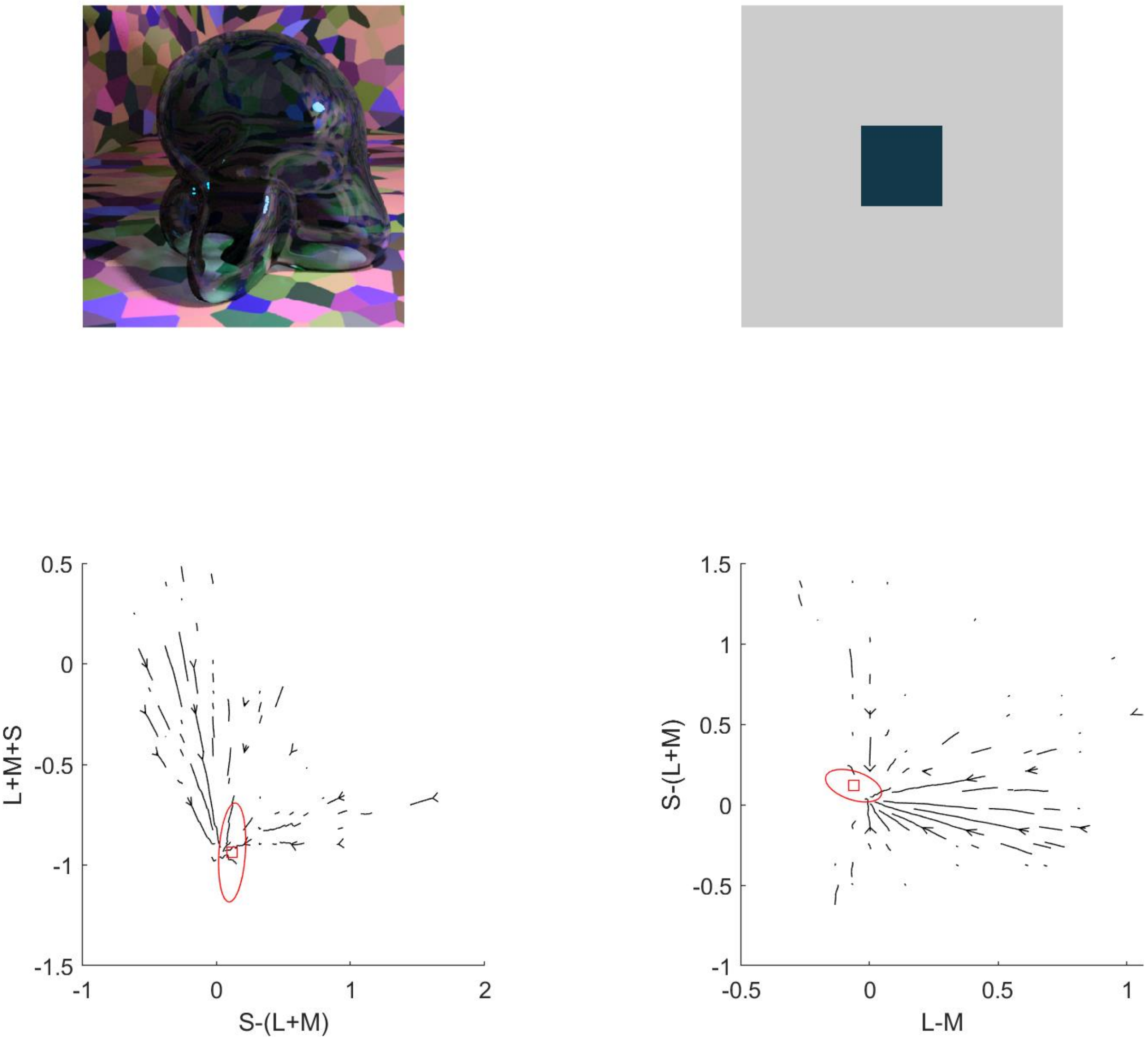

Figure 2: Low transmission green Glaven under the blue illuminant.

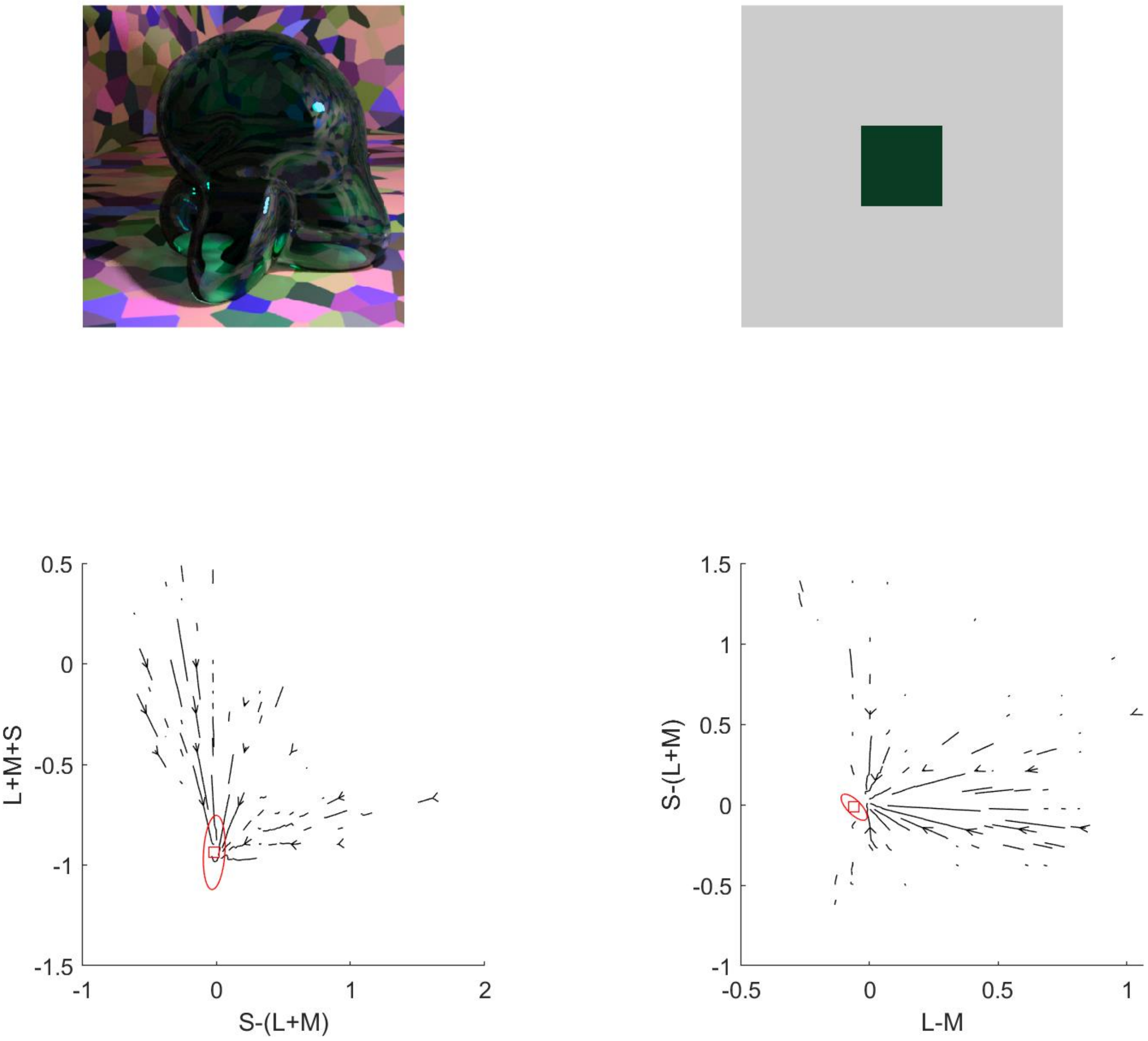

Figure 3: Low transmission red Glaven under the blue illuminant.

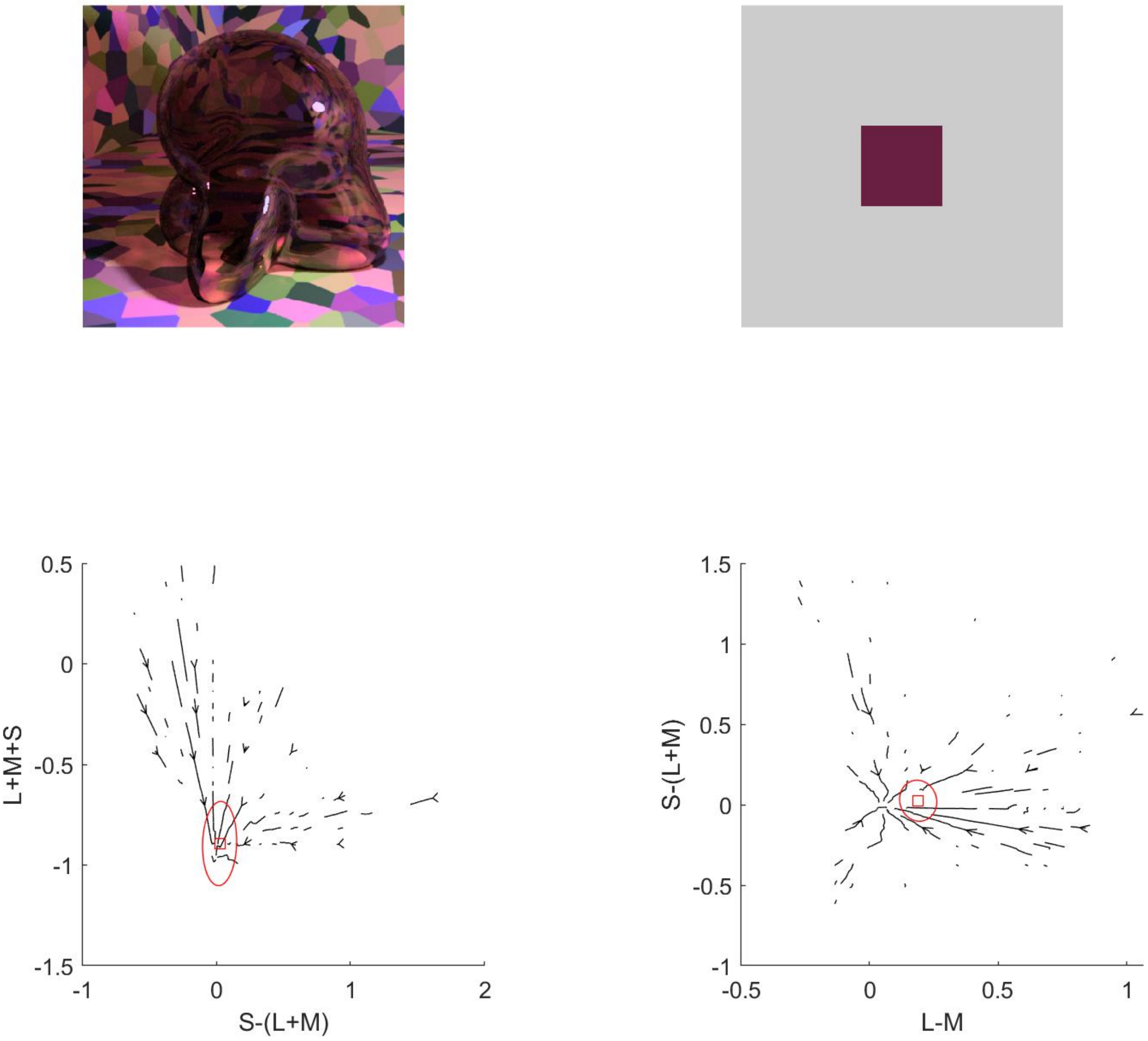

Figure 4: Low transmission yellow Glaven under the blue illuminant.

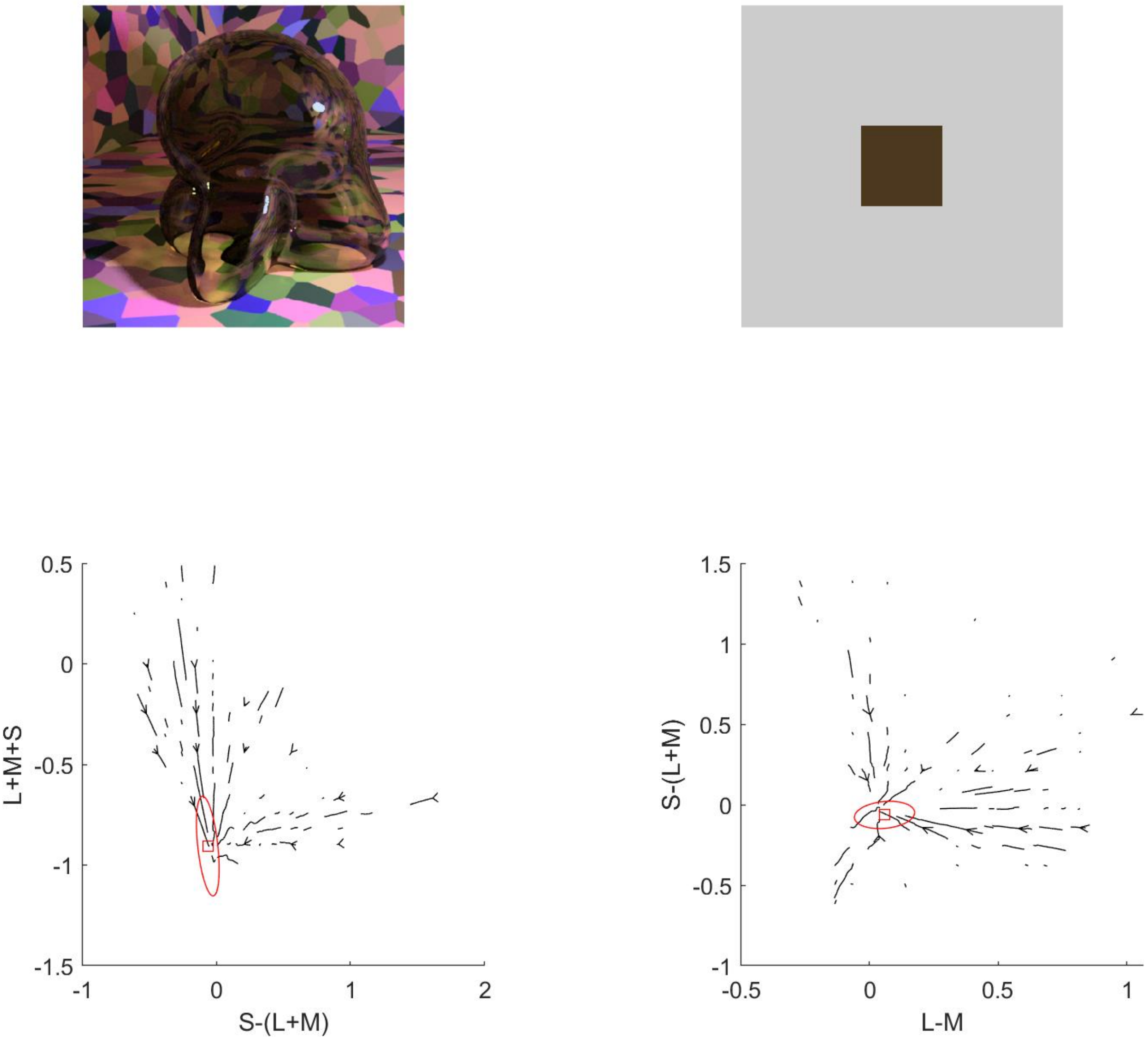

Figure 5: Low transmission blue Glaven under the white illuminant.

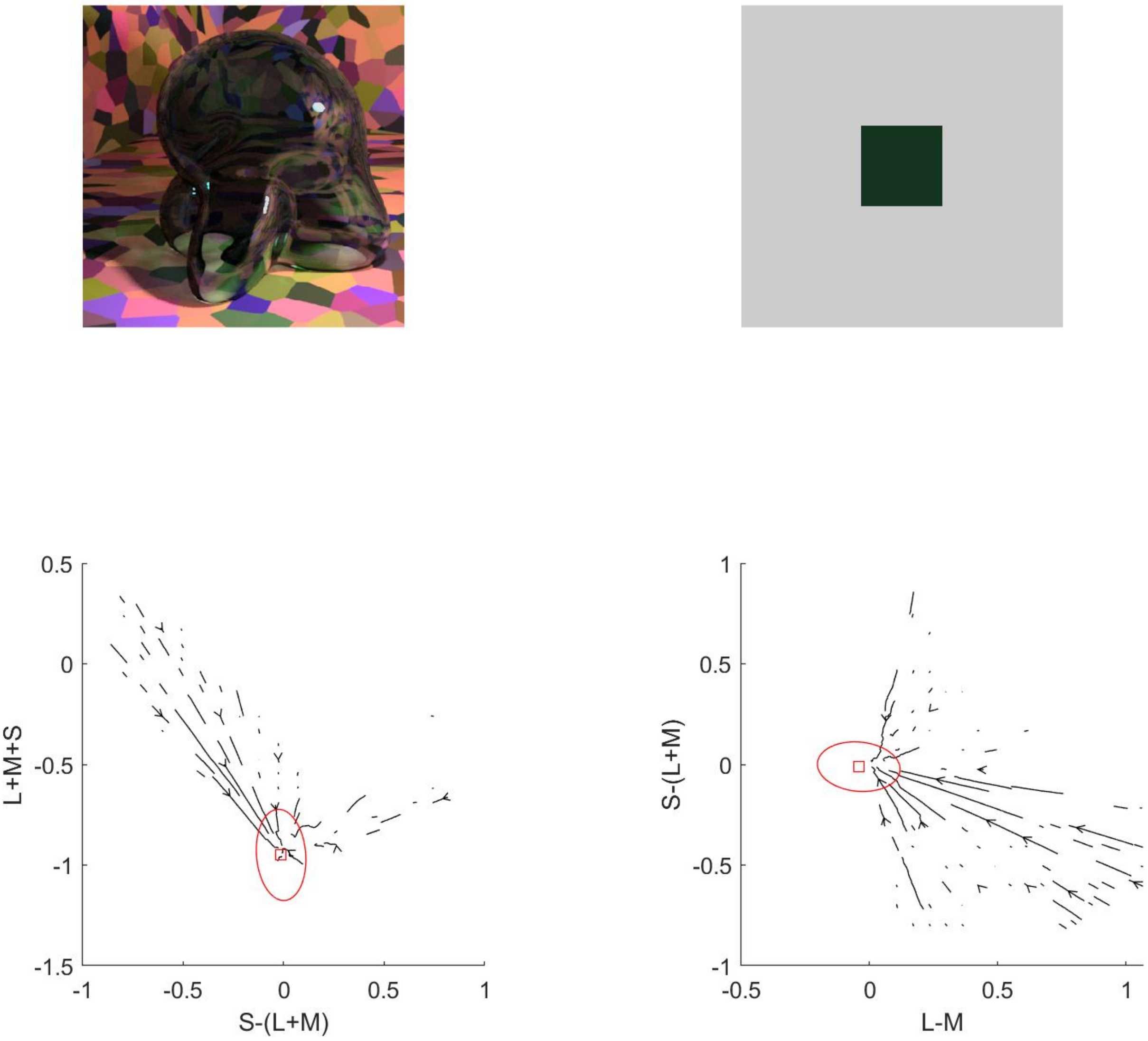

Figure 6: Low transmission green Glaven under the white illuminant.

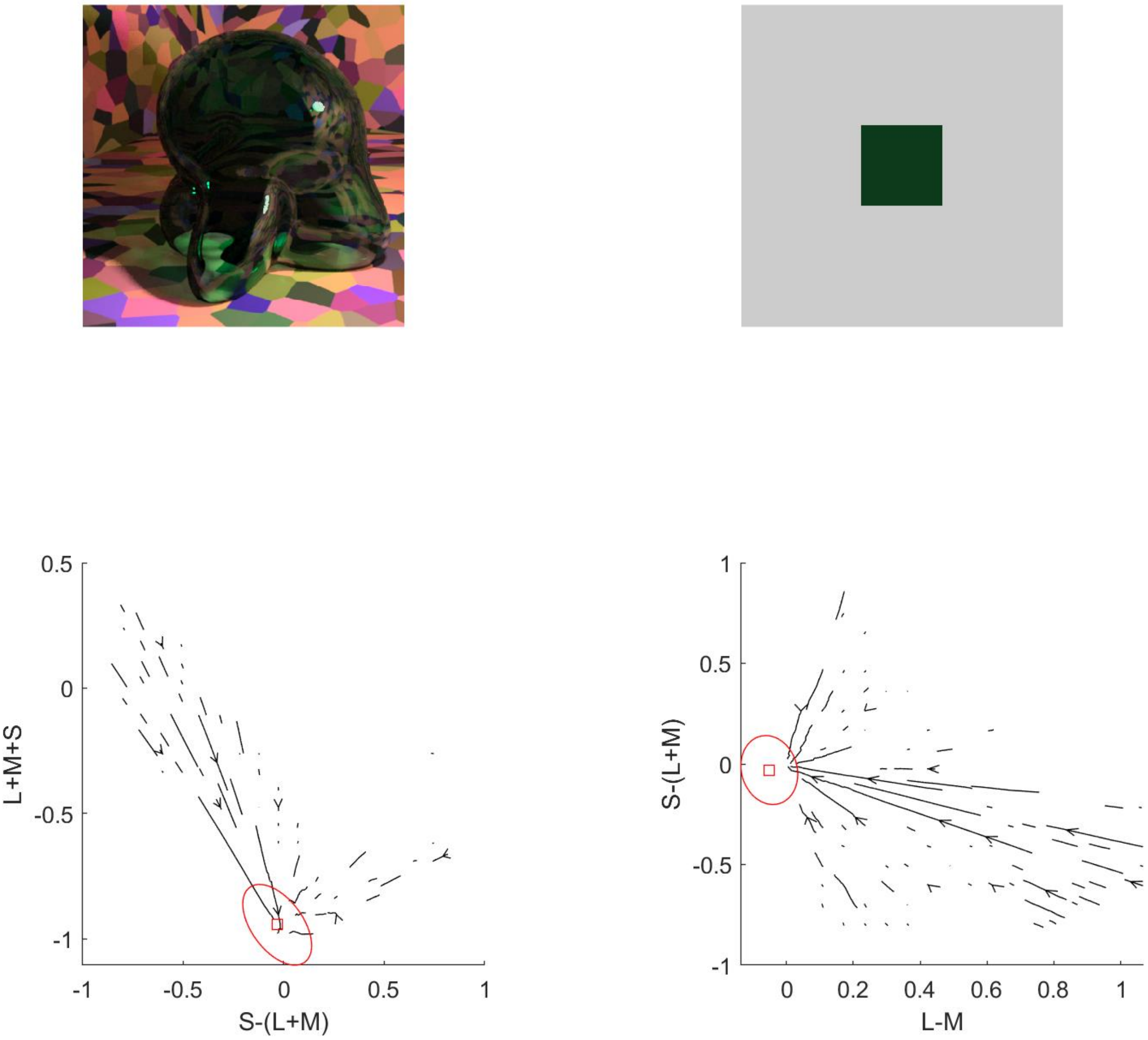

Figure 7: Low transmission red Glaven under the white illuminant.

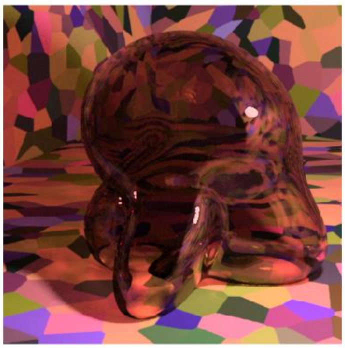

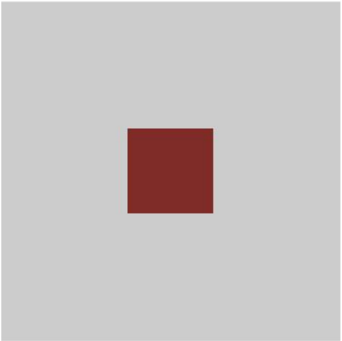

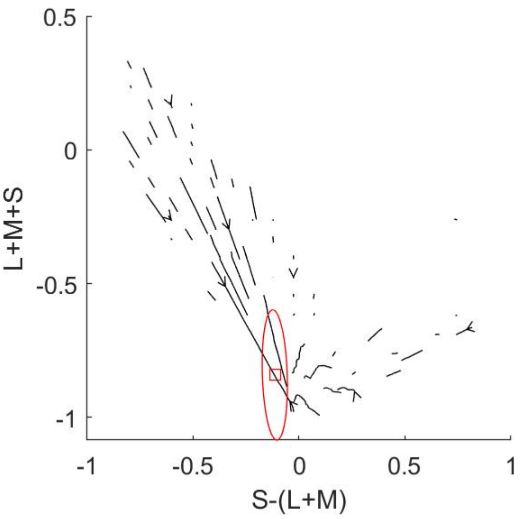

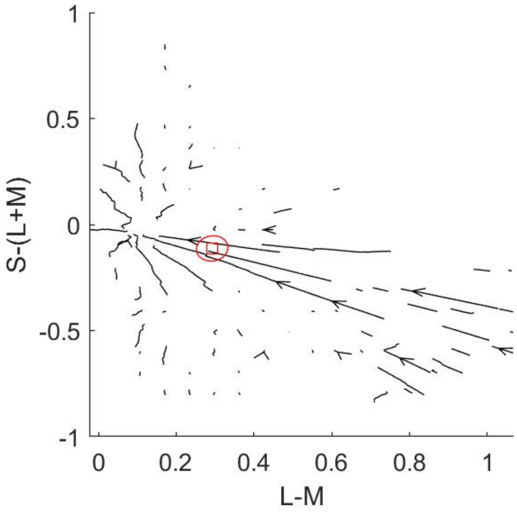

Figure 8: High transmission blue Glaven under the blue illuminant.

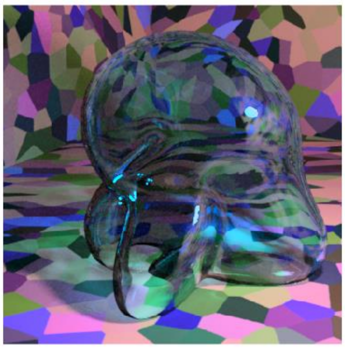

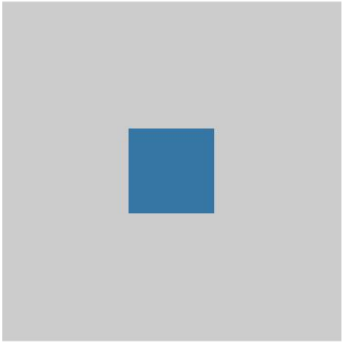

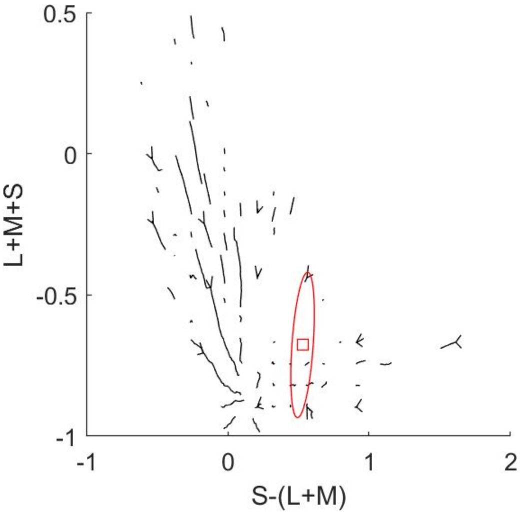

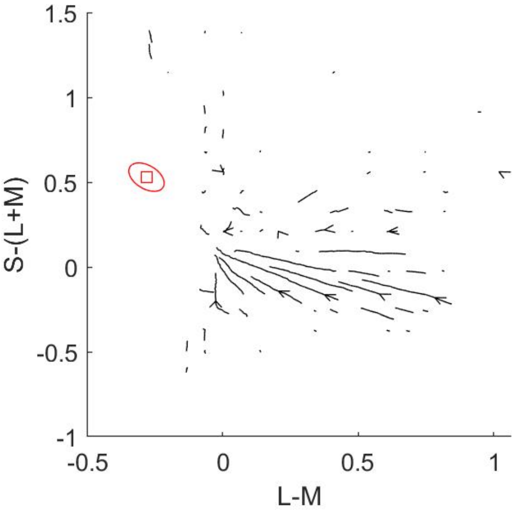

Figure 9: High transmission green Glaven under the blue illuminant.

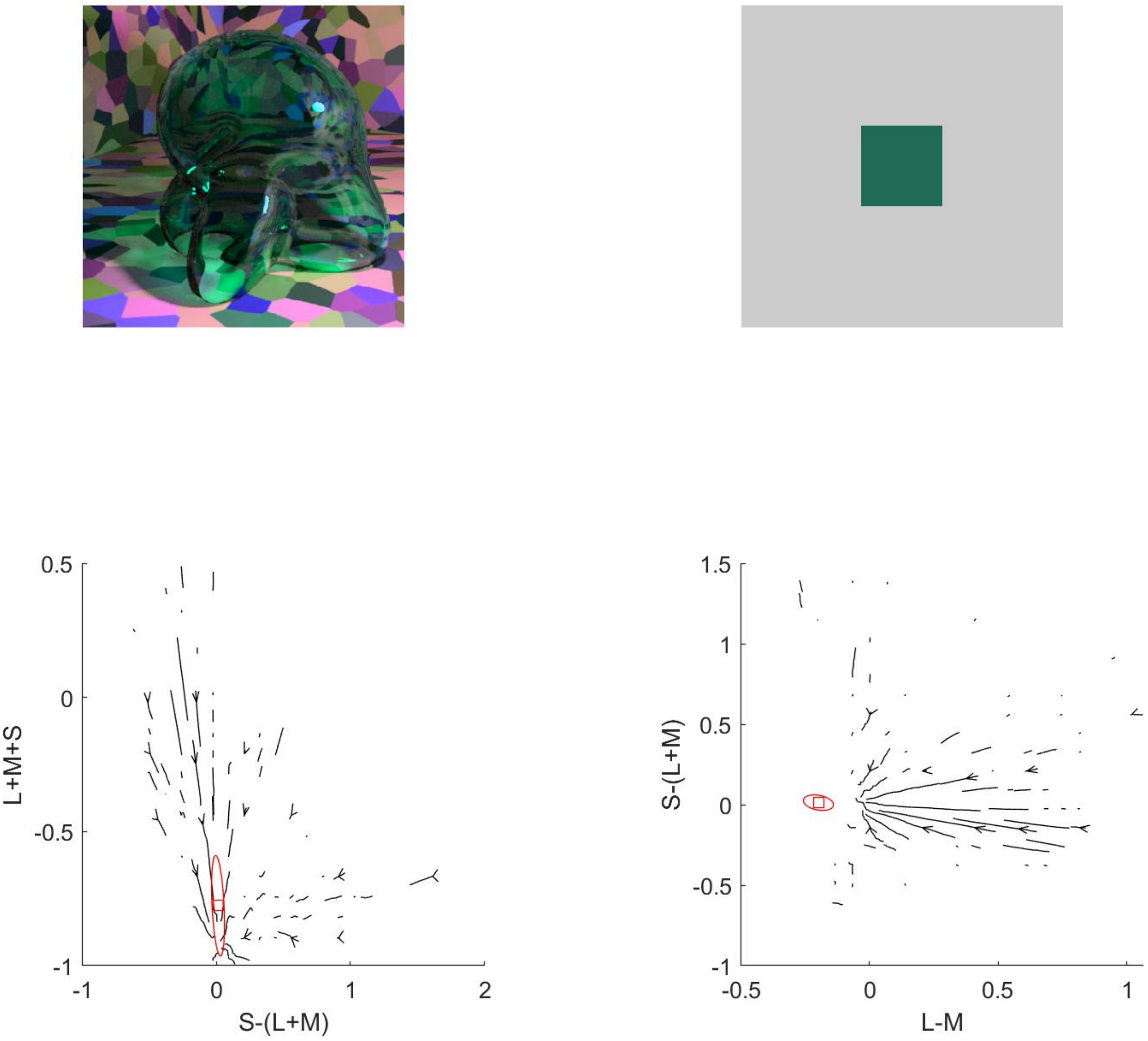

Figure 10: High transmission red Glaven under the blue illuminant.

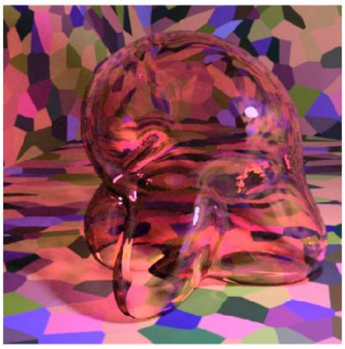

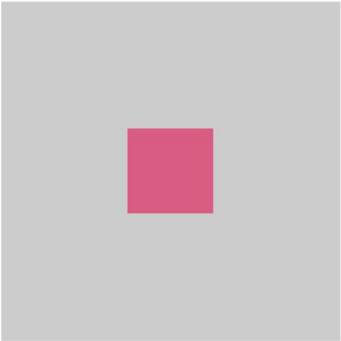

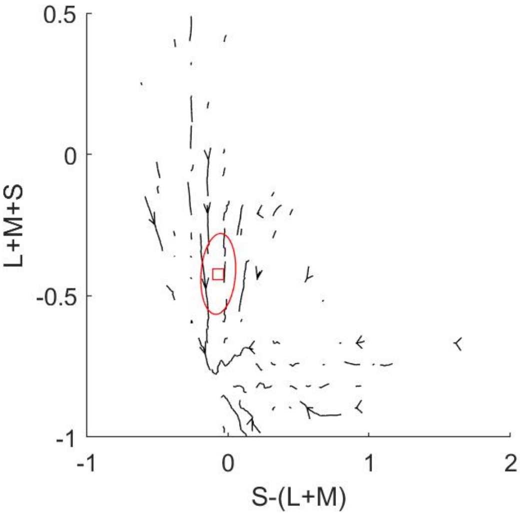

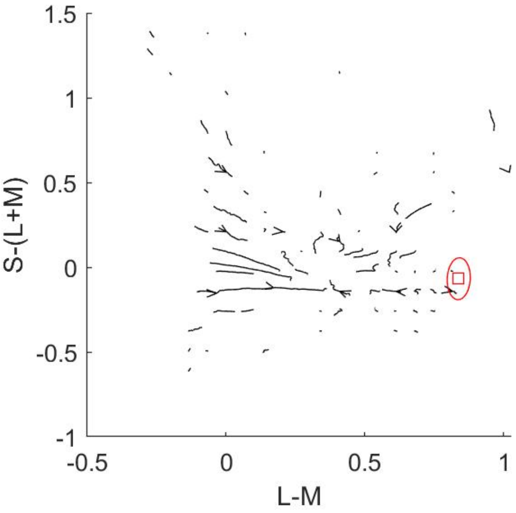

Figure 11: High transmission yellow Glaven under the blue illuminant.

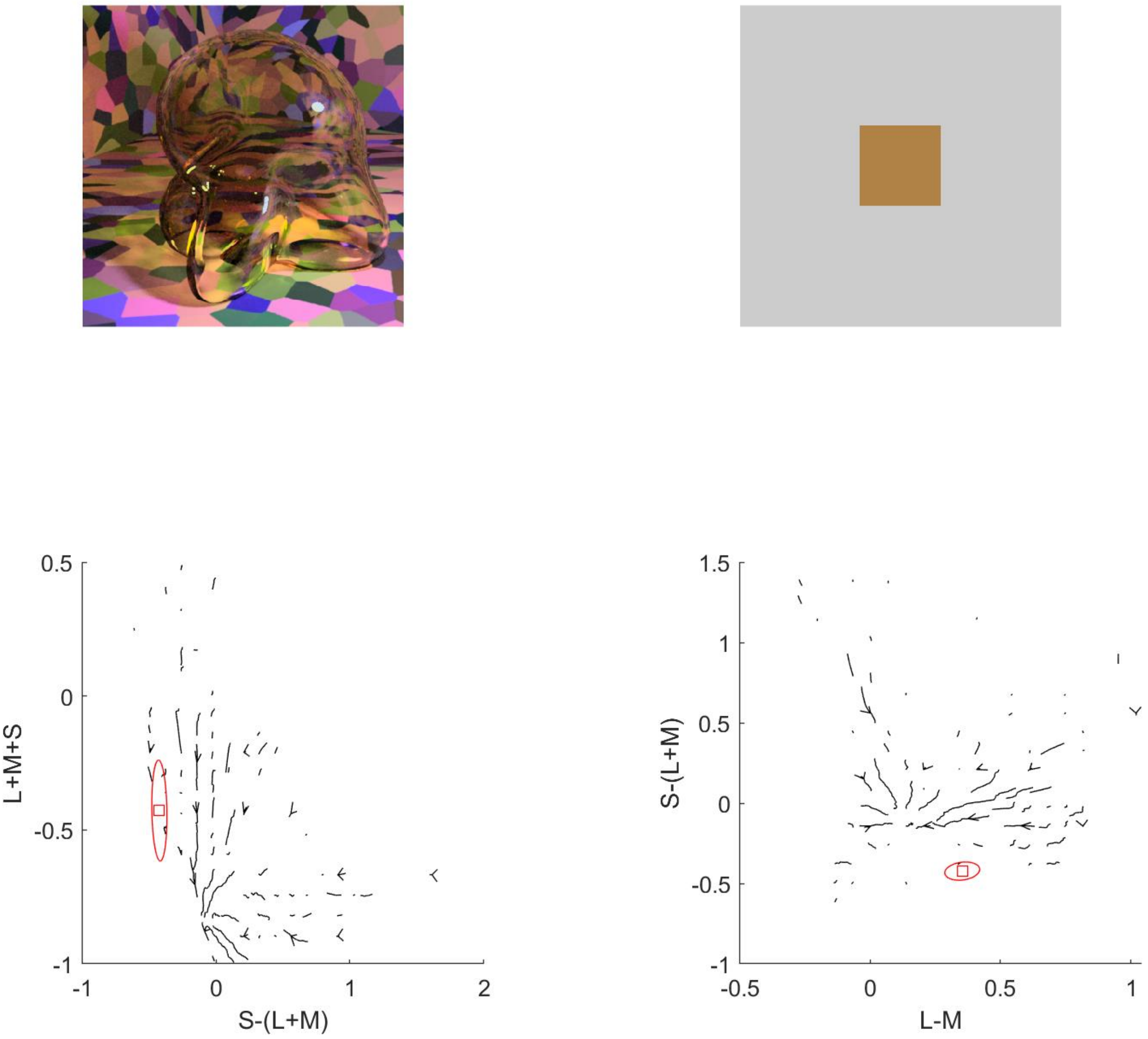

Figure 12: High transmission blue Glaven under the white illuminant.

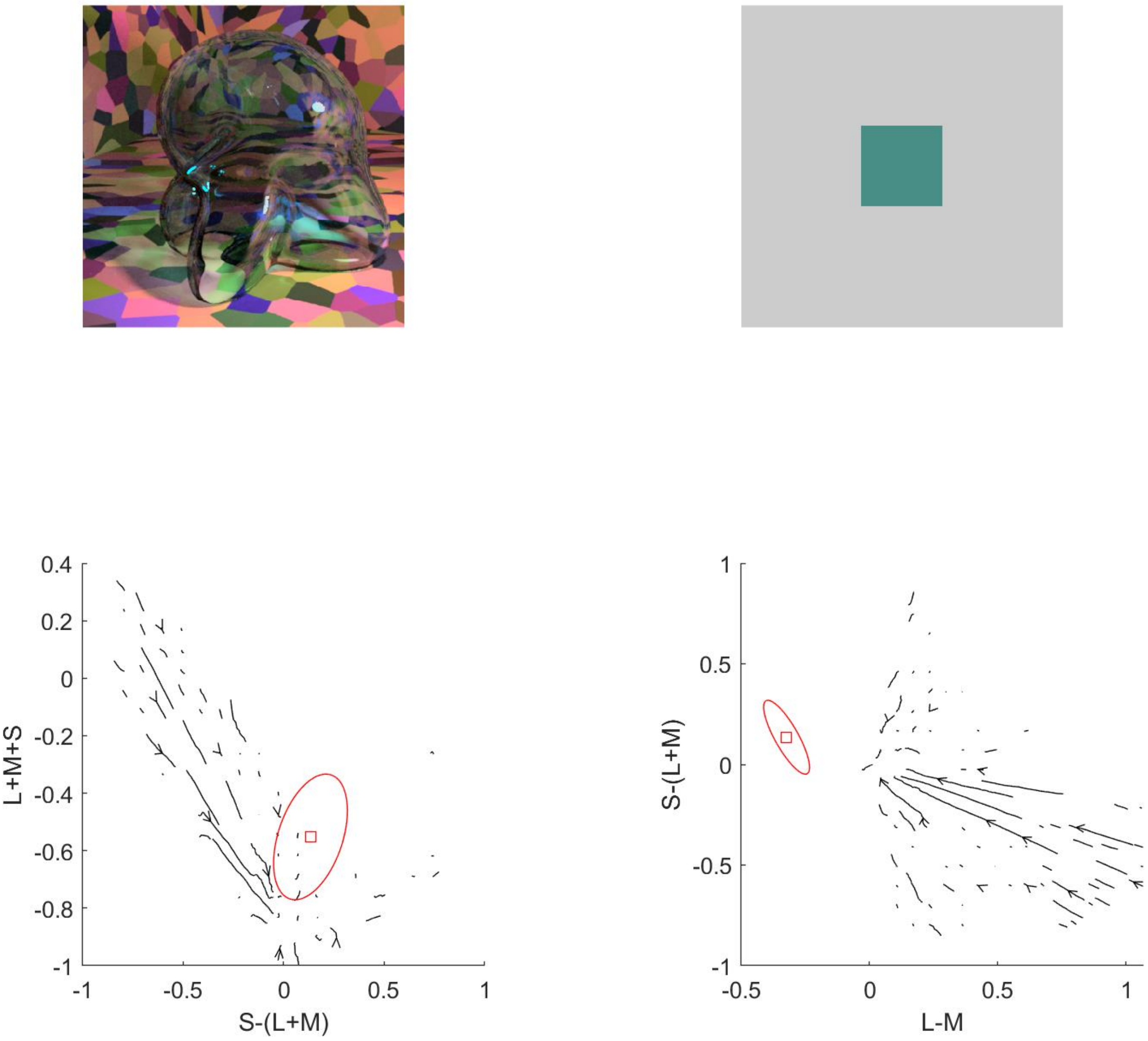

Figure 13: High transmission green Glaven under the white illuminant.

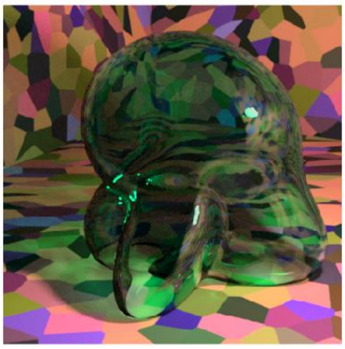

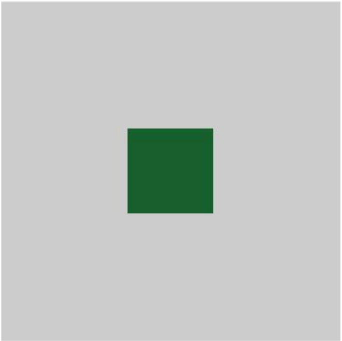

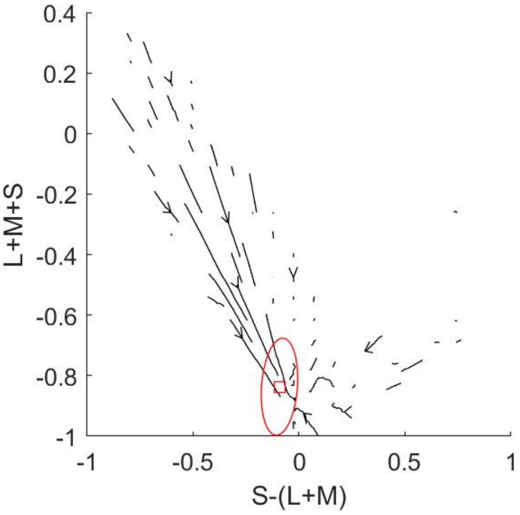

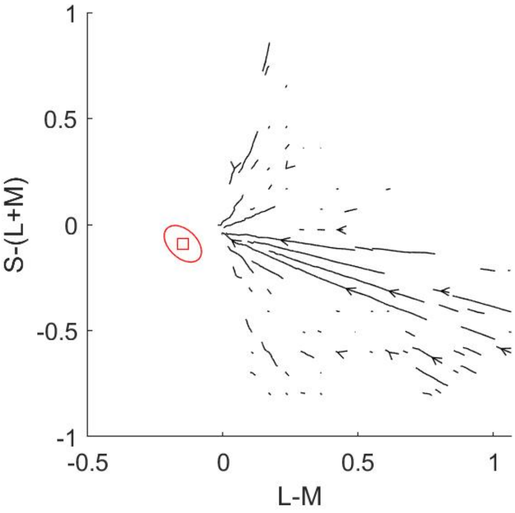

Figure 14: High transmission red Glaven under the white illuminant.

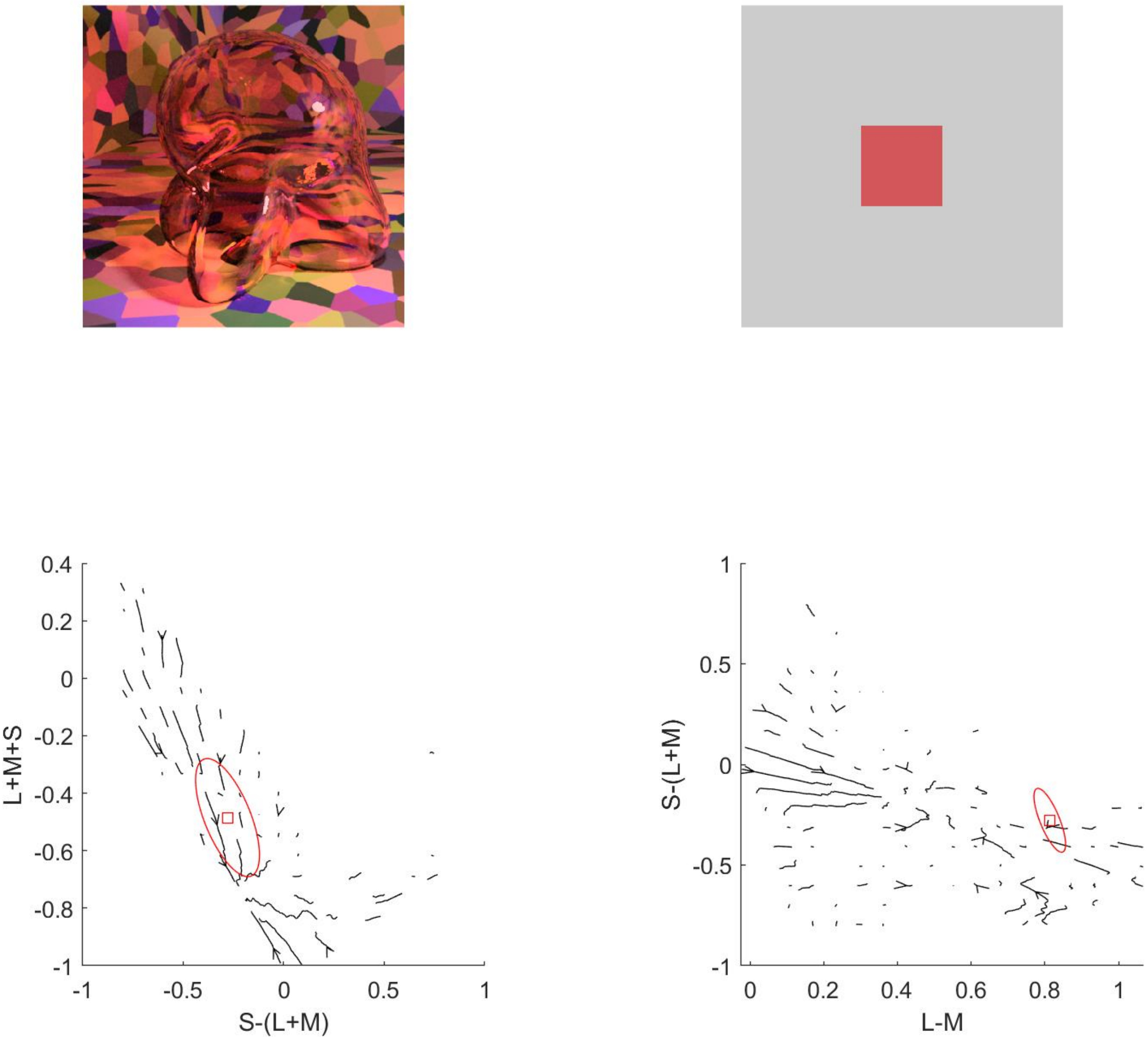

Supplement: Supplement 1 [file jovi-21-5-20_s001.pdf]
